# Genome-Wide Analyses Revealed Remarkable Heterogeneity in Pathogenicity Determinants, Antimicrobial Compounds, and CRISPR-Cas Systems of Complex Phytopathogenic Genus *Pectobacterium*

**DOI:** 10.3390/pathogens8040247

**Published:** 2019-11-20

**Authors:** Dario Arizala, Mohammad Arif

**Affiliations:** Department of Plant and Environmental Protection Sciences, University of Hawaii at Manoa, Honolulu, HI 96822, USA; arizala@hawaii.edu

**Keywords:** *Pectobacterium*, comparative genomics, pathogenicity determinants, antimicrobial compounds, CRISPR-Cas, horizontal gene transfer, dynamic evolution

## Abstract

The *Pectobacterium* genus comprises pectolytic enterobacteria defined as the causal agents of soft rot, blackleg, and aerial stem rot diseases of potato and economically important crops. In this study, we undertook extensive genome-wide comparative analyses of twelve species that conform the *Pectobacterium* genus. Bioinformatics approaches outlined a low nucleotide identity of *P. parmentieri* and *P. wasabiae* with other species, while *P. carotovorum* subsp. *odoriferum* was shown to harbor numerous pseudogenes, which suggests low coding capacity and genomic degradation. The genome atlases allowed for distinguishing distinct DNA structures and highlighted suspicious high transcription zones. The analyses unveiled a noteworthy heterogeneity in the pathogenicity determinants. Specifically, phytotoxins, polysaccharides, iron uptake systems, and the type secretion systems III–V were observed in just some species. Likewise, a comparison of gene clusters encoding antimicrobial compounds put in evidence for high conservation of carotovoricin, whereas a few species possessed the phenazine, carbapenem, and carocins. Moreover, three clustered regularly interspaced short palindromic repeats-Cas (CRISPR-Cas) systems: I-E, I-F, and III-A were identified. Surrounding some CRISPR-Cas regions, different toxin and antitoxin systems were found, which suggests bacterial suicide in the case of an immune system failure. Multiple whole-genome alignments shed light on to the presence of a novel cellobiose phosphotransferase system (PTS) exclusive to *P. parmenteri*, and an unreported T5SS conserved in almost all species. Several regions that were associated with virulence, microbe antagonism, and adaptive immune systems were predicted within genomic islands, which underscored the essential role that horizontal gene transfer has imparted in the dynamic evolution and speciation of *Pectobacterium* species. Overall, the results decipher the different strategies that each species has developed to infect their hosts, outcompete for food resources, and defend against bacteriophages. Our investigation provides novel genetic insights that will assist in understanding the pathogenic lifestyle of *Pectobacterium*, a genus that jeopardizes the agriculture sustainability of important crops worldwide.

## 1. Introduction

Members of *Pectobacterium* genus are defined as plant pathogenic enterobacteria that are responsible for the most devastating diseases, including common soft rot, blackleg, wilt, and aerial stem rot [1,2]. Strains of this genus cause severe losses worldwide in field crops, diverse vegetables, fruits, and ornamental plants [3,4]. *Pectobacterium* ranks among the top 10 of the most hazardous plant pathogenic bacteria due to its destructiveness impact that is exerted in agriculture fields as well as during storage and transportation [5], especially on potato, the third main staple food crop [6] and the major host of several *Pectobacterium* species [4,7]. Species of this genus have been isolated from infected tissues, soil, ground water, and can be associated with fruit flies, which aids in their rapid dissemination [8,9]. 

In regards to its taxonomy, the *Pectobacterium* genus has been restructured and subjected to constant revisions. First characterized at the beginning of the 20th century, *Pectobacterium* was once considered to be part of the *Erwinia* genus. Subsequent analyses that were based upon 16S rDNA revealed three major phylogenetic clusters; as a result, *Brenneria* and *Pectobacterium* were proposed and conceived as new genera independent from *Erwinia* [10]. At that time, *Pectobacterium* harbored eight species: *P. carotovorum* subsp. *atrosepticum*, *P. c*. subsp. *betavasculorum*, *P. c.* subsp. *carotovorum*, *P. c.* subsp. *odoriferum*, *P. c.* subsp. *wasabiae*, *P. cacticidum*, *P. chrysanthemi*, and *P. cypripedii* [10]. In 2003, three subspecies were elevated to the species level giving rise to *P. atrosepticum*, *P. betavasculorum*, and *P. wasabiae* [11]. Concurrently, highly virulent Brazilian strains were isolated from potato and named *P. c*. subsp. *brasiliense* [12]. Later, *P. chrysanthemi* was reclassified as *Dickeya chrysanthemi* [13], whereas *P. cypripedii* was transferred to the genus *Pantoea* [14]. Years later, the causal agent of canker in *Actinidia chinensis* was isolated and named *P. c.* subsp. *actinidiae* [15]. Furthermore, some isolates with preference for monocotyledonous hosts were designated as *P. aroidearum* [16]. In a similar way, *P. wasabiae* was split and *P. parmentieri* was established [17]. Recently, strains that were previously confirmed as *P. carotovorum* were re-classified and established as *P. polaris* [18], *P. peruviense* [19], and *Candidatus* P. maceratum [20]. At the time of writing, *Pectobacterium* is comprised of thirteen species, although there was not even a draft genome of *P. cacticidum* in any databases. 

The host range of these thirteen species is quite divergent. *P. betavasculorum*, *P. cacticidum*, *P. wasabiae*, and *P. c.* subsp. *actinidiae* exhibit a narrow host range, as they are restricted to sugar beet, cacti, horseradish, and kiwifruit, respectively [11,15,17]. *P. aroidearum* shows a preference for aroid hosts [16]. *P. atrosepticum* and *P. parmentieri* seem to be limited to potato [17,21]. Likewise, *P. peruviense*, *P. polaris*, and *C.* P. maceratum so far have only been found in potato. In contrast, *P. c.* subsp. *carotovorum* and *P. c.* subsp. *brasiliense* have been isolated from a great variety of vegetables (tomato, pepper, cucumber, eggplant, carrot, lettuce, onion, and Chinese cabbage), as well as other solanaceous hosts, including potato and tobacco [22,23,24]. *P. c.* subsp. *odoriferum* was thorough to affect solely chicory, but posterior studies confirmed this pathogen in different vegetables [25]. 

Each *Pectobacterium* species exerts their characteristic symptoms, such as slimy, wilting, black rot, and gummosis through the deployment of pathogenicity determinants, which suppress the plant defense mechanisms. The secretion systems, proteases, the plant cell wall degrading enzymes (PCWDE), effector molecules, flagella-based motility, iron acquisition systems, polysaccharides, cell attachment proteins, the type IV pilus, phytotoxins, 3-hydroxy-2-butanone pathway (3H2B), and other virulent elements that are encoded by individual genes comprise these virulence-associated promoters [2,26,27]. *Pectobacterium* sp. produce specific compounds that display antimicrobial activity, either with a broad antibiosis spectrum, like carbapenem and phenazine, or with a narrow effect, including carotovoricin and carocins [21,28,29,30,31,32]. The transcription of pathogenicity factors and antimicrobial components is controlled by regulators that operate jointly as a complex network.

High throughput sequencing has unveiled a set of genomic data to conduct bacterial comparative genomic analyses. *P. atrosepticum* SCRI1043 was the first soft rot bacterium being sequenced [21]. Later, the complete genomes of *P. c*. subsp. *carotovorum* PCC21 [33], *P. parmentieri* SCC3193 [26], *P. wasabiae* CFBP 33304 [17], *P. carotovorum* SCC1 [34], *P. polaris* [18], and *P. c.* subsp. *brasiliense* BZA12 [24] were analyzed. At the time of writing this article (September 2018), 18 complete and 90 high quality draft genomes of the *Pectobacterium* species have been published in the GenBank genome database. The easy access of several published genomes illuminates new strategies for exploring the genetic datasets, giving rise to comparative genomics [35,36]. Bacterial genome comparison is a powerful tool for studying evolutionary biology, taxonomy, phylogeny, and it can provide insights to distinct niches adaptation [35,37]. Furthermore, comparing genomes has allowed for identifying the core repertoire of virulence genes shared by related organisms and, likewise, to locate gene clusters or genome islands exclusive of species or even unique of a strain [38]. Putative genes that promote specific interactions between bacteria and their cognate plant hosts have also been uncovered by using comparative genomics approaches [35]. To date, few comparison studies have been conducted regarding *Pectobacterium* [8,24,27,39]. However, none of them have compared the virulence mechanisms, antimicrobial gene clusters, and the diversity of the immune system clustered regularly interspaced short palindromic repeats-Cas (CRISPR-Cas) across all species that currently comprise the *Pectobacterium* genus in detail.

When considering this background, the primary purpose of the present investigation was to develop an extensive genome-wide comparative analysis among the 12 species of the genus *Pectobacterium*. First, we evaluated the main genomic features and DNA structural properties of genome atlases in all species. Second, we assessed the current taxonomy within the *Pectobacterium* genus based upon the average nucleotide identity (ANI), the DNA-DNA hybridization (dDDH), phylogenetic tree, proteome comparison, codon and amino acid usage profiles, and core-genome dendrogram. Third, we compared the sequence organization and arrangements of gene clusters defined as pathogenicity determinants and those clusters that are implicated in the synthesis of antimicrobial compounds. Fourth, we in silico identified the distinct CRISPR-Cas systems harbored in 34 *Pectobacterium* genomes. Our data aim to shed light on novel genetic regions and elucidate remarkable divergences that are involved in pathogenesis, antimicrobial components, and immune adaptative system (CRISPR-Cas), which will contribute to understanding the evolutionary dynamics and virulent lifestyle of *Pectobacterium*, a complex and heterogeneous genus that is responsible for disastrous economic losses in important agricultural crops. 

## 2. Materials and Methods

### 2.1. Strains Information, Genome Sequencing, Assembly and Annotation

All of the genome assemblies used in this study were retrieved from NCBI GenBank genome database, and Table S1 provides details. The strains that were used for genome sequencing originated worldwide, including Scotland, China, Belarus, South Korea, Denmark, Peru, Finland, Canada, France, Israel, Pakistan, Russia, Japan, Norway, the Netherlands, and the United States of America (Table S1); the strains were isolated from *Solanum tuberosum*, *Brassica rapa*, *Cucumis sativus*, *Apium graveolens*, *Cichorium intybus*, *Actinidia deliciosa*, *Ornithogalum dubium*, *Eutrema wasabi*, *Beta vulgaris*, and *Brassica oleracea* (Table S1). The genomes were sequenced while using different sequencing platforms from Illumina MiSeq (Illumina Inc., San Diego, CA, USA) to PacBio (Pacific Biosciences of California, Inc., Menlo Park, CA, USA); PHRAP, SOAPdenovo, SPAdes, Newbler, Velvet and HGAP (Hierarchical Genome Assembly Process) were used to assemble the genomes (Table S1). The genomes of two neighbor species, *Dickeya zeae* and *D. solani*, were also included for phylogenomic and pairwise nucleotide identity. Geneious 10.2.4 was used to visualize and analyse the genome sequences and feature annotations. The Reference Sequence (RefSeq) numbers for either the International Nucleotide Sequence Database Collaboration (INSDC) or Whole-genome-sequence (WGS) projects were used to download the genomes.

### 2.2. Phylogenomic Analyses

With the aim of assessing the level of discrepancy among the *Pectobacterium* species, the Average Nucleotide Identity (ANI) based on the Nucleotide MUMmer algorithm (ANIm) was computed and then determined in the JSpecies Web Server with default parameters [40]. Additionally, the in silico DNA-DNA hybridization (dDDH) or genome-to-genome distance between the genomes were calculated while using the Genome-to-Genome Distance Calculator (GGDC) version 2.1 with the recommended formula 2 and the BLAST + alignment criteria [41,42]. ANI and dDDH data were both compiled in a single matrix and visualized as a color-coded heatmap while using MORPHEUS (https://software.broadinstitute.org/morpheus). The cut-off values of 95% and 70% were assigned as a species delineation framework for ANI and dDDH, respectively.

A comprehensive phylogenetic relationship across the genus *Pectobacterium* was established by aligning ~18 kb of genome region comprising nucleotide sequences from 12 housekeeping genes, namely chromosomal replication initiator protein DnaA (*dnaA*—1382 bp), DNA polymerase III subunit beta (*dnaN*—1101 bp), DNA polymerase III subunit gamma/tau (*dnaX*—1101 bp), elongation factor G (*fusA*—2102 bp), glyceraldehyde-3-phosphate dehydrogenase A (*gapA*—996 bp), DNA gyrase subunit A (*gyrA*—2633 bp), DNA gyrase subunit B (*gyrB*—2411 bp), DNA recombination and repair protein RecA (*recA*—1074 bp), DNA repair protein RecN (*recN*—1661 bp), DNA-directed RNA polymerase subunit alpha (*rpoA*—990 bp), RNA polymerase sigma factor RpoD (*rpoD*—1826 bp), and RNA polymerase sigma factor RpoS (*rpoS*—990 bp). The alignment was individually performed for each gene while using the MUSCLE algorithm [43]. Afterward, all of the individual alignments from 12 genes were concatenated using Geneious. Lastly, the phylogenetic analysis based on the concatenated sequences was performed while using MEGA X [44]; evolutionary history along the *Pectobacterium* genus was inferred using the Neighbor-Joining clustering algorithm [45]. The Maximum Composite Likelihood method was applied for determining the evolutionary distances among the isolates [46]. The bootstrap test based on 1000 replicates was computed to estimate reliability [47], and the associated taxa clustered were expressed as percentage units next to the branches of outputted tree. In addition, two closest taxon *D. solani* IPO2222, as well as *D. zeae* EC1, were included in this study as an outgroup for obtaining a comprehensive taxonomic position within the *Pectobacterium* species. 

### 2.3. Basic Statistics and Genomic Profile Features

The basic parameters for each genome, such as GC content, number of coding sequence regions (CDS), tRNA genes, pseudogenes, and others features (Table 1), were fetched from the data that were available in both the NCBI GenBank database and the Bioinformatic Resource Center PATRIC [48,49]. Additionally, genome atlases were only constructed for complete genomes to illustrate the different structural components of each genome sequence, such as GC skew, stacking energy, intrinsic curvature, position preference, global direct, and indirect repeats. The previous parameters were visualized and drawn as a circle plot by using the script atlas_createConfig while using CMG-Biotools [50].

### 2.4. General Genomic and Proteome Analyses

A comparative genomic ring plot was executed using BLAST Ring Image Generator (BRIG) [51] to illustrate the degree of homology among the twelve *Pectobacterium* species. *P. atrosepticum* SCRI1043 genome was taken as reference for the nucleotide BLAST genome comparison. In proteomics analyses, an overall pairwise proteome comparison that was based on BLAST [52] was performed to construct a BLAST matrix, which is a visual graphic showing the total number of proteins common within and between a given set of proteomes [53]. The entire analyses were performed while using the CMG-Biotool package. The script matrix_createConfig was used to create a BLAST matrix set-up file in an XML format; this file was used as an input data file to plot the BLAST matrix using the program ‘matrix’. Finally, the generated matrix plot was visualized as a color scale heatmap that represented the percentage wise numerical homology values across all compared proteomes. BLAST hits with at least 50% of identical matches and 50% of coverage were considered to be significant. 

### 2.5. Codon and Amino Acid Usage and Pan-Core Genome Analyses

The comparative analyses that were based on the amino acid and codon usage between the *Pectobacterium* species were determined using BioPerl modules and implemented with the script stats_usage using CMG-Biotools package. Two heatmaps depicted the different clustering patterns across the *Pectobacterium* genomes, in terms of amino acid and codon usage, were generated in R: The R Project for Statistical Computing (https://www.r-project.org/). The number of Pan and Core genes across the *Pectobacterium* species were analyzed and calculated while using the BLAST algorithm [52] with 50% of cutoff values for either cover or identity percentage parameters. The output plot was constructed using CMG-Biotools pipeline package and then implemented in the program ‘pancoreplot’. Afterward, a pan-genome phylogenetic tree that was based upon those gene families shared between all *Pectobacterium* species was created and plotted using the script pancoreplot_tree [50]. 

### 2.6. Genomic Islands (GI) Prediction

The Integrative Conjugative Elements (ICE), such as Horizontal Acquired Islands (HAI), were studied and predicted using the IslandViewer 4 webserver [54], which allowed for an interactive visualization of genomic islands (GI) as either in circular or linear plots by integrating four different and accurate predictor tools IslandPath-DIMOB [55], SIGI-HMM, IslandPick [56], and Islander [57].

### 2.7. Analyses of Pathogenicity, Virulence, Antimicrobial Gene Clusters and CRISPR-Cas

The clusters that were involved in diverse virulence and pathogenicity events, including type secretion systems (TSS), phytotoxins, iron uptake, polysaccharides biosynthesis, flagella encoding genes, cell attachment, and agglutination were screened and compared across all the *Pectobacterium* species. The clusters comparison was performed while using Proteome Comparison tool of the Pathosystems Resource Integration Center (PATRIC) web server [49]. Two separate genome comparison tables were generated for *P. atrosepticum* SCRI1043 and *P. parmentieri* SCC3193, since both species were used as reference genomes. On the other hand, the biosynthetic gene clusters encoding secondary metabolites related to the synthesis of phytotoxins, antibiotics and antimicrobial compounds were predicted while using antiSMASH 4.0 [58]. In addition, BAGEL4 was applied as a genome mining tool for identifying putative gene clusters involved in bacteriocin biosynthesis [59]. Finally, the synteny and different rearrangements between the genomic clusters were visualized as linear arrow comparison plots, generated while using Easyfig v2.2.3 [60]. Circa was used to illustrate a summary of different clusters involved in different functions of *Pectobacterium* species, their shared homology, and connections (http://omgenomics.com/circa). The Clustered Regularly Interspaced Short Palindromic Repeats (CRISPRs) arrays, as well as the type of CRISPR associated proteins (Cas) systems, were predicted while using CRISPRCasFinder [61]. CRISPRs loci features, such as total size, number of spacers and direct repeats, length, and consensus sequence of direct repeats were also analyzed and corroborated by using CRISPRDetect [62] and the NCBI GenBank database. The different CRISPR-Cas systems that were analyzed in this study were illustrated and manually drawn. 

## 3. Results

### 3.1. General Genomic Features and DNA Structure Properties

Overall genomic features, such as genome size, GC (guanine-cytosine) content, conserved coding sequences, number of RNA genes, pseudo-genes, proteins with assigned function in metabolic pathways, as well as virulence factors and antibiotic resistance genes, were retrieved from either NCBI GenBank database or PATRIC [49]. The corresponding data set of the twelve species: *Pectobacterium atrosepticum* SCRI1043, *P. carotovorum* subsp. *carotovorum* PCC21, *P. carotovorum* subsp. *brasiliense* BC1, *P. carotovorum* subsp. *odoriferum* BC S7, *P. carotovorum* subsp. *actinidiae* KKH3, *P. arodiearum* PC1, *P. parmentieri* SCC3193, *P. wasabiae* CFBP 3304, *P. betavasculorum* NCPPB 2795, *P. polaris* NIBIO 1392, *P. peruviense* IFB5232, and *Candidatus* Pectobacterium maceratum PB69, was compiled in a single table (Table 1). The length of the genomes ranged between 5.16 Mb to 4.68 Mb, with *P. parmentieri* and *P. betavasculorum*, possessing the largest and smallest genomes, respectively. The GC content percent was almost same among *Pectobacterium* species (around 51%); *P. c.* subsp. *carotovorum* showed maximum GC content of 52.2%. The highest numbers of conserved coding sequence (CDS) were observed in *P. pamentieri*, with 4,449 confirmed gene sequences, whereas the lowest number of CDS were found in *P. c.* subsp. *odoriferum* with 3,855 confirmed gene sequences. However, *P. c.* subsp. *odoriferum* was found to maintain the highest number of pseudogenes (385) among the *Pectobacterium* species (Table 1). The rRNA and tRNA genes were similar in all species, except for *P. peruviense*, which expressed the lowest number of 5S RNA, 16S RNA, and 23S RNA genes (2, 1, 2, respectively). *P. parmentieri* and *P. peruviense* showed the highest number of proteins with an assigned metabolic function (835 CDS), followed by *P. atrosepticum* (829 CDS). The highest known virulence and predicted antibiotic resistance CDSs were found in *P. parmentieri* and *P. atrosepticum*, each possessing 53 and 60 genes, respectively. The recently identified species C. P. *maceratum* possesses the highest number of 35 CDS for drug targets. 

Genome atlases were generated for eight complete genomes to contrast relevant characteristics based upon structural DNA properties (Figure S1). Atlases were not created for *P. c.* subsp. *actinidiae*, P. *betavasculorum*, *P. peruviense*, and *C*. P. maceratum due to the unavailability of the complete genomes. GC skew pointed out the start and the end point of replication and displayed as blue and pink for G and C content, respectively; generally, Gs represent the leading strand. Among the atlases, *P. atrosepticum* harbored the most global direct and indirect repeats (24 blue and 18 red lines), whereas the atlas of *P. aroidearum* only presented 13 direct and seven invert global repeats. The position reference parameter is a value that associates DNA regions of greater rigidity (0.15 and featured in pink in the atlas) or flexibility (0.14 and featured with a green line in the atlas), according to the position preference of the DNA within the nucleosome [50]. The DNA atlas of *P. aroidearum* extends over 59 regions with high positional preference, as indicated by characteristic rigidity that appears pink on the atlas.

The stacking energy, on the other hand, is a -3.82 kcal/mol to 14.59 kcal/mol based parameter, which allows for distinguishing between the regions that were susceptible to de-stacking (less negative values or closer to 0) and regions of greater stability (more negative values) [50]. The intrinsic curvature is a parameter that quantifies the degree of DNA wrapping around a histone core and it ranges between the values of 0 and 1: *P. c*. subsp. *carotovorum* and *P. parmentieri* exhibited the strongest and weakest wrapping intensity, respectively (as indicated with the prevalence of blue lines). Pink, red, and dark blue colors in the atlases highlight specific regions with high positions preference, stacking energy, and high intrinsic curvature characteristics.

### 3.2. Phylogenetic Relationships within Pectobacterium Genus

**Figure 1:**
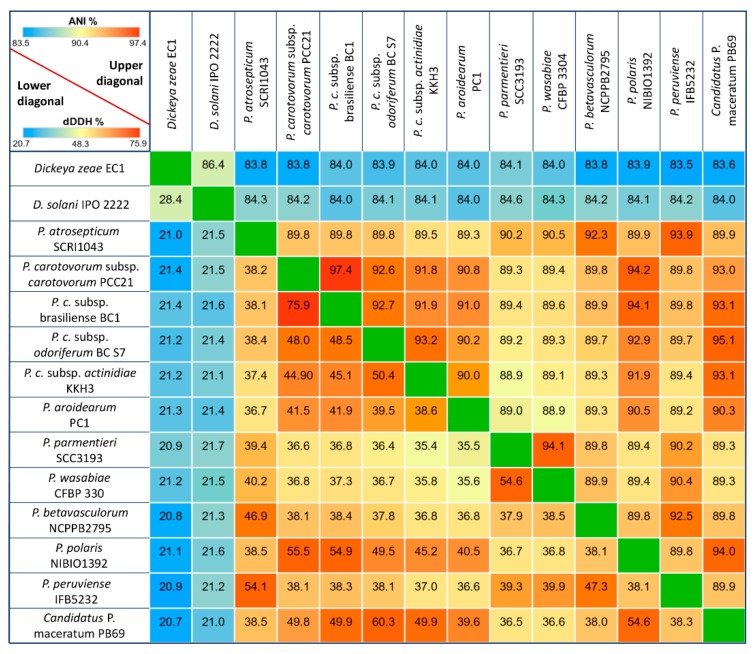
Pairwise heatmap of *Pectobacterium* species based on average nucleotide identity (ANI) and digital DNA-DNA hybridization (dDDH). Both ANI and dDDH are represented as percentage values among the twelve *Pectobacterium* species and two *Dickeya* species (*D. zeae* EC1 and *D. solani* IPO 2222). The upper diagonal displays ANI data, whereas the lower diagonal depicts the *in silico* dDDH data. The cut-off values for species delineation are 95 and 70% for ANI and dDDH, respectively.

The genome homology between the *Pectobacterium* and *Dickeya* taxa oscillated from 83.5 to 97.4% based on ANI analysis effectuated with the MUMmer algorithm (Figure 1) in the JSpecies software. The highest degree of genome similarity was observed between *P. carotovorum* subsp. *carotovorum* PCC21 and *P. carotovorum* subsp. *brasiliense* with an ANI value of 97.4%, and related species *P. parmentieri* SCC3193 and *P. wasabiae* CFBP 3304 exhibited a high homology of 94.1%. On the other hand, *P. parmentieri* presented the highest divergence with subspecies *P. c* subsp. *actinidiae* KKH3, showing a low ANI value of 88.9%. 

The digital DNA-DNA hybridization (dDDH) analysis allows for inferences to be drawn between the sequenced genomes relative to genome distances and provides analogues data within the range of 20.7 (with *Dickeya* species) to 75.9%, a value that is consistent with the ANI results (Figure 1). In general, all of the dDDH values were below 70%. Additionally, the lowest dDDH values were observed by comparing the genome distances between the *Dickeya* and *Pectobacterium* isolates. Likewise, a 60.3% genome-to-genome distance was estimated between *C.* P. maceratum and *P. c* susbp. *odoriferum*. A divergence trend was also observed for *P. parmentieri* and *P. wasabie* with *P. c*. subsp. *actinidiae* and *P. aroidearum*, respectively. 

**Figure 2:**
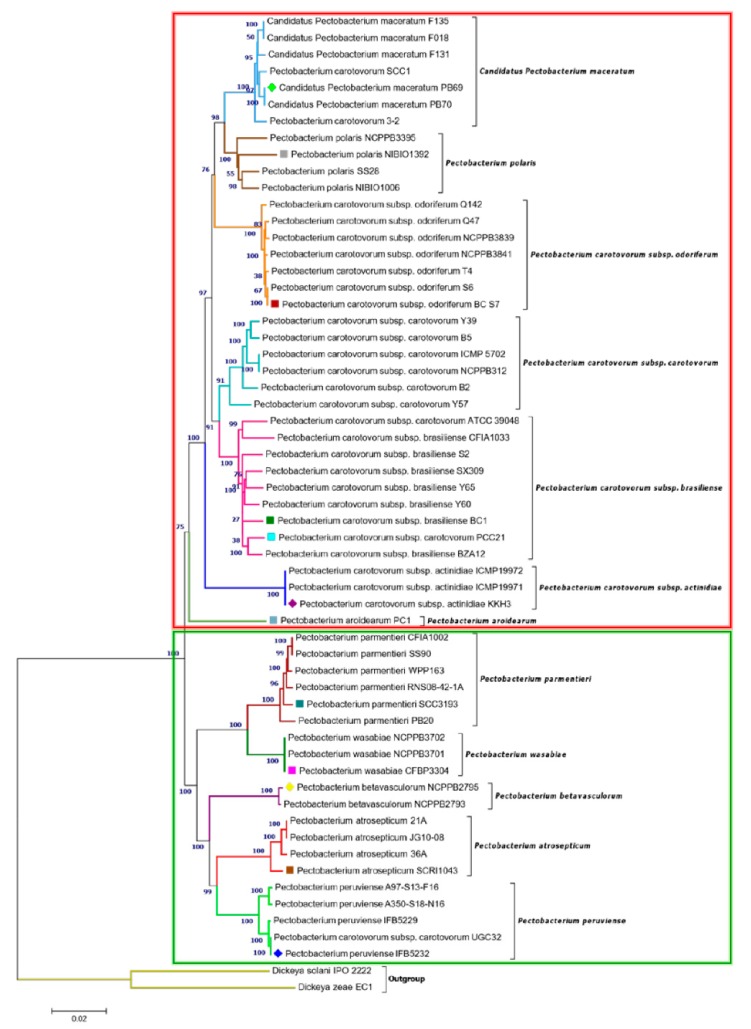
Concatenated phylogenetic tree illustrating the relationships among all *Pectobacterium* species. A total of 59 strains from all known *Pectobacterium* species were selected to generate Neighbor-Joining tree based on 12 housekeeping genes: *dnaA*, *dnaN*, *dnaX*, *fusA*, *gapA*, *gyrA*, *gyrB*, *recA*, *recN*, *rpoA*, *rpoD*, and *rpoS*. The alignment of individual genes was performed using MUSCLE, and a concatenated tree was subsequently generated after aligning all 12 genes (total length ~ 18,269 bp). The Maximum Composite Likelihood method was applied to determine the evolutionary distances, with each node being supported by a bootstrap of 1,000 replicates to assess reliability. Bootstrap values are displayed next to the branches. The branches were colored to highlight the distinct strains clustered in each species. The dendrogram was drawn to scale, and all positions containing gaps and missing data were eliminated. DNA sequences of *D. zeae* EC1 and *D. solani* IPO 2222 were included as out-groups to root the tree. The evolutionary analysis was developed in MEGA X. Colored square and diamond shapes indicate the complete and draft genomes included in the study, respectively. Two main clades were identified and marked with dark red and green rectangles.

In addition, a phylogenetic tree that was based on concatenated sequences of twelve housekeeping genes (*dnaA*, *dnaN*, *dnaX*, *fusA*, *gapA*, *gyrA*, *gyrB*, *recA*, *recN*, *rpoA*, *rpoD*, and *rpoS*) was constructed, as in Figure 1. with MEGA X using the Neighbor-Joining method (Figure 2) to evaluate the current evolutionary relationships among all *Pectobacterium* species described to date. The 12 gene sequences of 57 *Pectobacterium* strains and 2 *Dickeya* species (used as out-group) were extracted from the respective species genomes, retrieved from NCBI GenBank genome database (Table S1), and aligned using MUSCLE algorithm. The generated dendogram revealed two main clades: the first clade (red dashed square) featured an aggregate of the *P. carotovorum* subspecies as well as *C*. P. maceratum, *P. polaris*, and the aroids plant pathogen, *P. aroidearum*; the second clade (green dashed square) featured the species *P. parmentieri*, *P. wasabiae*, *P. betavasculorum*, *P atrosepticum*, and *P. peruviense* (Figure 2). In clade I, a major cluster was formed from two sub-clusters, while *P. aroidearum* PC1 was isolated. The first sub-cluster allocated the genomespcies *C*. P. maceratum as a close relative of *P. polaris*. In the phylogenetic tree below, the node of the *P. polaris* and *C*. P. maceratum strains lineage with *P. c*. subsp. *odoriferum*; it can be concluded that the three species evolved from a common ancestor.

**Figure 3:**
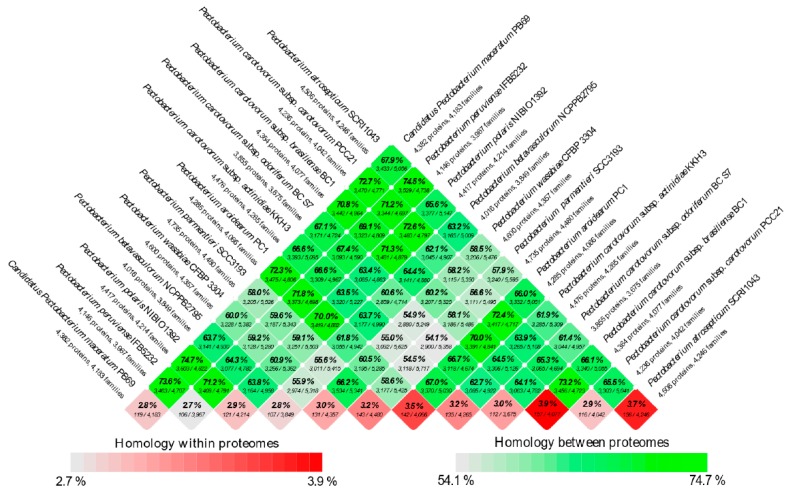
BLAST matrix between and within total proteomes of *Pectobacterium* genus. Pairwise protein comparison was performed using BLAST and protein coding sequences were compared among the genomes. A BLAST hit was considered significant when 50% of the alignment revealed identical matches and covered at least 50% Alignment. If two protein sequences were similar based on cut-off value, they were grouped within the same protein family. The color scale saturation changes from dark green to light green indicated the degree of homology between the proteomes, whereas the color scale from dark red to light red showed the homologous hits within the proteome itself (internal paralogs at the bottom row of the matrix). Twelve genomes of different *Pectobacterium* species were compared in the analysis.

The second sub-cluster of the clade I grouped *P. c*. subsp. *carotovorum* and *P. c*. subsp. *brasiliense*. Interestingly, two strains, PCC21 and ATCC 39048, available in the NCBI as *P. c*. subsp. *carotovorum*, clustered within the group of *P. c*. subsp. *brasiliense* strains, suggesting a misidentification of these both strains. The three isolates of kiwi *P. c*. subsp. *actinidiae* clustered in a separate node of the two sub-clusters mentioned above. Likewise, two distinct clusters can be observed in clade II. The first cluster comprised *P. parmentieri* and *P. wasabiae*, as expected. The second cluster included the strains from *P. betavasculorum*, *P. atrosepticum* and *P. peruviense*. In this cluster, the newly categorized species, *P. peruviense* formed a sub-cluster along with *P. atrosepticum*, whereas *P. betavasculorum* split into a different lineage. This outcome is concordant with that of ANI and dDDH. Based on analyses, it can be concluded that *P. peruviense* and *P. atrosepticum* both co-evolved, but eventually diverged into two separate species. The pairwise comparison of total protein coding genes among *Pectobacterium* species demonstrated a range of 54.1% to 74.7% shared proteins, with the lowest value being represented between *P. c*. subsp. *odoriferum* and *P. parmentieri*, and the highest between *P. polaris* and *C.* P. maceratum (Figure 3). Likewise, the proteins (3,855) and protein families (3,675) that were used for the comparison were the lowest for *P. c*. subsp. *odoriferum* (Figure 3). In agreement with ANI, dDDH, and phylogenetic analysis outcomes, *P. peruviense* shared 74.5% proteins in common with *P. atrosepticum*. The sugar beet pathogen, *P. betavasculorum*, showed the least similarity (55.6% to 64.4%) when compared to the rest of the species. 

### 3.3. Genomic Evolution of Pectobacterium Species—Analysis of Codon and Amino Acid Usage

**Figure 4:**
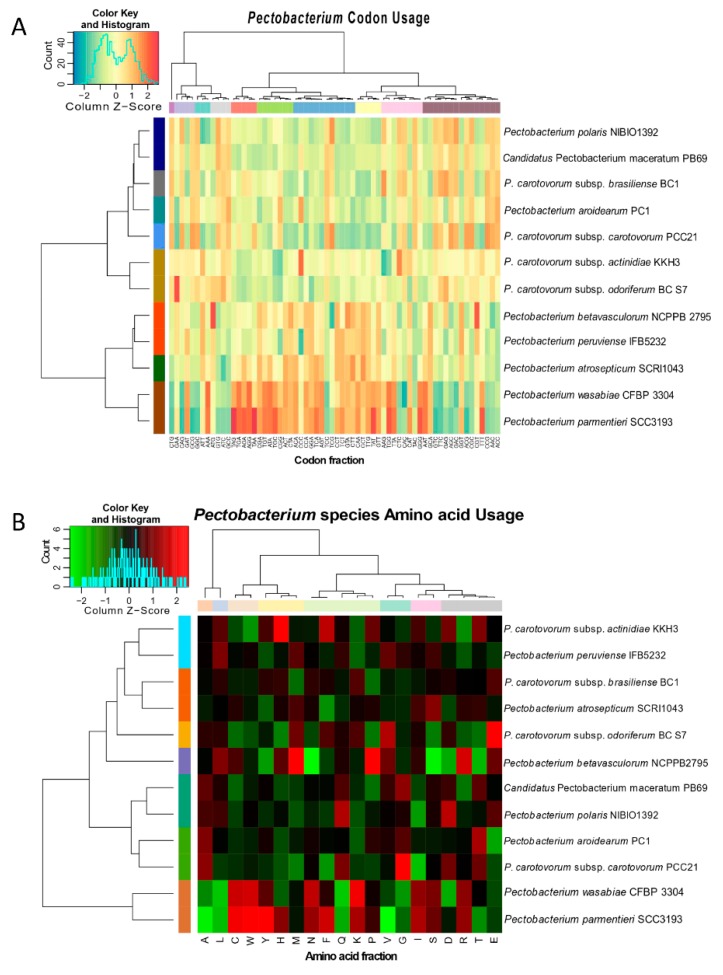
Heat maps featuring the relationships across the twelve *Pectobacterium* species based on (**A**) codon usage and (**B**) amino acid usage. The identified gene data was plotted and used to construct heat maps in R with heatmap.2 function. The species were reorganized according to the profiles of codon and amino acid usage. Dendrograms at the top and the left side of both heat maps were drawn to illustrate the species clustering pattern. The row and column colors next to both dendrograms highlighted the different clusters observed after analysis. Histograms indicated the scale colors based upon the percentages of codon usage and amino acid frequencies.

The preference of codon usage varies between organisms and even between species. Therefore, the codon usage pattern constitutes an exclusive property for each species and genome [63]. Studying the divergences of codon usage bias contributes to understanding the basis of pathogen evolution and their adaptation to specific niche [63]. The bias score was evaluated at +1 if 100% in the third position corresponds to G or C, or −1 if the bias score was 100% of A or T in the third position. Overall, all *Pectobacterium* sp. genomes exhibited bias towards either G or C nucleotides (Figure S2); however, the value ranges were low (0.1133 to 0.1894). The AT content for all species was low, and it ranged from 46.50 % (*P. c.* subsp. *carotovorum*) to 48.34% (*P. parmentieri*). *P. c.* subsp. *carotovorum*, *P. polaris*, and *P. c.* subsp. *brasiliense* showed a slightly higher bias score towards G usage when compared with the other bacteria (0.1894, 0.1827, and 0.1809, respectively). The calculated percentages of the codon and amino acid profiles, among the *Pectobacterium* sp., were compiled and visualized in two dimensional heatmaps that were created in R (Figure 4). Besides, rose plots on amino acid and codon usage of each genome were also created (Figures S3 and S4). 

The CTG, GAA, and CAG were frequently used by all species, while the CCG, AAC, and ACC codons exhibited the least usage (Figure S3). Furthermore, amino acid composition revealed that the Leucine (L) and Alanine (A) were used in higher frequencies in all the *Pectobacterium* species, while the amino acids Tryptophan (W) and Cytosine (C) displayed the minimum values (Figure S4). The heat map that was generated based on codon compositions (Figure 4A) demonstrated concordant clustering, as revealed by phylogenetic analysis (Figure 2). Thus, for instance, *P. betavasculorum*, *P. peruviense*, *P. atrosepticum*, *P. wasbiae*, and *P. parmentieri* grouped in the same clade, while the remaining species formed a distinct lineage. In amino acid usage heat map (Figure 4B), two main clades were formed; *P. wasabiae* and *P. parmentieri* grouped separately. In disagreement with the previous phylogenic analysis, the amino acid tree displayed a close relationship between *P. c.* subsp. *actinidiae* and *P. peruviense.*

### 3.4. Analysis of the Pangenome and Coregenome

**Figure 5:**
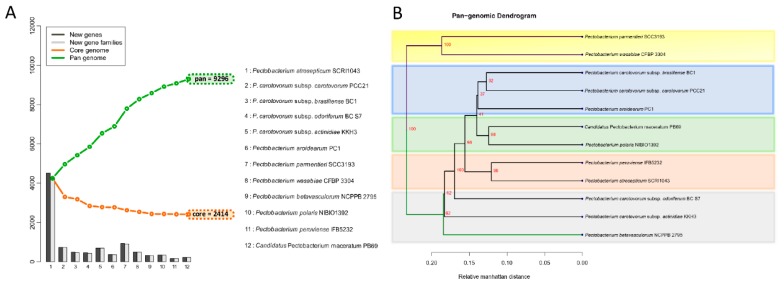
Pan and core genome analysis among the *Pectobacterium* species. (**A**) Pan-Core genome plot. The pan and core genome were calculated using BLAST with a cutoff of 50% identity and 50% coverage. The total number of genes, conformed for pan and core genomes, are represented with green and orange squares, respectively. The bars in the plot represent the number of new genes (dark grey bars) or new gene families (light grey bars) found after the addition of each new genome. Therefore, the size of the pan-genome increased with each new genome. While the number of core genome decrease with the addition of each new genome. (**B**) Pan-genome tree. The dendrogram illustrates the grouping among the *Pectobacterium* species based upon shared gene families (core genome) defined in the pan and core genome analysis. Two main clades were defined, and the main branches of each clade are highlighted in green and violet colors. Shaded color rectangles were added manually to emphasize how the species were clustered according to the core genome.

The proteomes of the twelve species were extracted, and a BLASTp was conducted against each genome. The genes were assigned as conserved between the species only if they fulfilled the criteria of at least 50% of sequence similarity over no less than 50% of the length of the alignment. The 12 *Pectobacterium* species showed an accumulative number of 9296 gene families (Figure 5A). 

It is important to clarify that either the new gene(s) or new gene families were determined based on the BLASTp hit against the previously included genomes in the list (x-axis in the plot). Hence, the order in which each species was added influenced the addition of novel gene families, as depicted in the bar graphs. However, the total number of genes in core and pan genomes were always the same, regardless of the species order. In contrast with the pan-genome, after the addition of each genome in the analysis, the total number of genes decreased—12 *Pectobacterium* genomes depicted a total of 2414 core genes (Figure 5A); this number corresponds to the genes conserved or orthologous across all the genomes. The size of the pan-genome was more than three times when compared to the core genome, indicating great genetic heterogeneity and evolutionary dynamics among the *Pecotbacterium* species. In addition, a pan-genome phylogenetic tree (Figure 5B) based on the reported 2414 common genes was constructed. Two main clades were found, the first cluster included *P. parmentieri* and *P. wasabiae*, with a 100% bootstrap value (Figure 5B). *P. c.* subsp. *carotovorum* (Pcc), *P. c*. subsp. *brasiliense* (Pcb), and *P. aroidearum* formed a group together, while *C.* P. maceratum with *P. polaris* were closer to each other. *P. peruviense* with *P. atrosepticum* formed their group. The remaining species did not constitute a defined cluster. 

### 3.5. Versatile Pathogenicity Mechanisms have been Acquired Through Integration of Genomic Islands (GIs) Across the Pectobacterium Species

**Figure 6:**
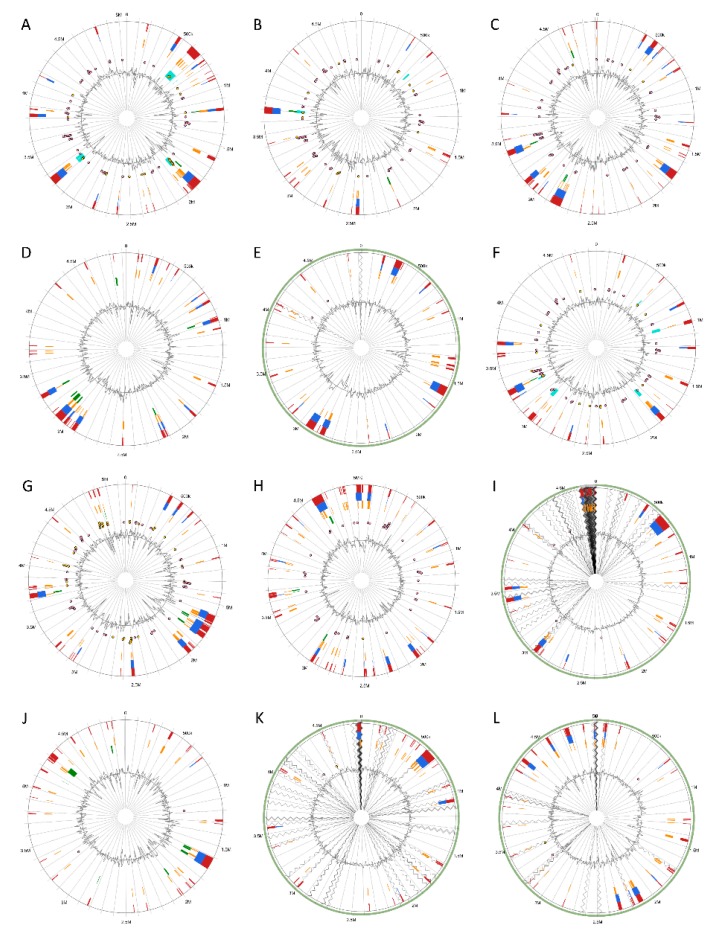
Circular visualization of predicted Genomic Islands (GIs) among the 12 *Pectobacterium* species. Starting from the top left row to the bottom right—illustrated the circular GIs plots for the species: (**A**) *P. atrosepticum* SCRI1043, (**B**) *P. carotovorum* subsp. *carotovorum* PCC21, (**C**) *P. carotovorum* subsp. *brasiliense* BC1 (**D**) *P. carotovorum* subsp. *odoriferum* BC S7, (**E**) *P. carotovorum* subsp. *actinidiae* KKH3, (**F**) *P. aroidearum* PC1, (**G**) *P. parmentieri* SCC3193, (**H**) *P. wasabiae* CFBP 3304, (**I**) *P. betavasculorum* NCPPB 2795, (**J**) *P. polaris* NIBIO1392, (**K**) *P. peruviense* IFB5232 and (**L**) *C.* Pectobacterium maceratum PB69. The interactive visualization of distinct islands across the genomes are shown with colored blocks according to the description provided in the predictor tool: IslandPick, based on genome comparison (green); IslandPath-DIMOB, based on associated GIs features such as tRNAs, transposon elements, integrases and sequence bias (blue); SIGI-HMM, based on the codon usage bias with a Hiddden Markov model criterion (orange); Islander, based on the mapping of GIs in regards with the recurrent use of tRNA and tmRNA genes (turquoise); and, the integrated results of four tools (dark red). Antimicrobial resistance genes as well as pathogen-associated genes are also displayed as pink and yellow circular glyphs, respectively. Species with incomplete genomes, including *P. c.* subsp. *actinidiae*, *P. betavasculorum*, *P. peruviense*, and *C*. P. maceratum, the contigs were ordered against the closest relative reference genome (light green outer circle). The references were selected based upon the ANI and dDDH analysis; consequently, *P. atrosepticum* was used as the reference for *P. betavasculorum* and *P. peruviense*, *P. c*. subsp. *brasiliense* as the reference for *P. c*. subsp. *actinidiae*, and *P. polaris* as the reference for *C*. P. maceratum. Gray areas represented unaligned contigs.

Genomic Islands were predicted and visualized the genomic islands across the 12 *Pectobacterium* species; different circle plots depicting different GIs in each of the species (Figure 6). A total of 22 GIs was found for *P. atrosepticum* (Table S2). However, no method, which integrates IslandViewer 4, could identify HAI6, HAI10, HAI11, HAI14, and HAI15 documented by Bell et al. [21]. For the rest of the species, the following number of GIs were found: *P. c.* subsp. *carotovorum*—25 GIs (Table S3), *P. c.* subsp. *brasiliense*—33 GIs (Table S4), *P. c.* subsp. *odoriferum*—29 GIs (Table S5), *P. c.* subsp. *actinidiae*—23 GIs (Table S6), *P. aroidearum*—24 GIs (Table S7), *P. parmentieri*—40 GIs (Table S8), *P. wasabiae*—45 GIs (Table S9), *P. betavasculorum*—32 GIs (Table S10), *P. polaris*—30 GIs (Table S11), *P. peruviense*—20 GIs (Table S12), and *C.* P. maceratum—28 GIs (Table S13). In *P. c.* subsp. *carotovorum*, GIs ranging from 4.11 kb with 6 CDS (GI-23) to 50.30 kb with 36 CDS (GI-24) were predicted (Table S3). In *P. c.* subsp. *brasiliense*, the GI-18 with a length of 94.09 kb harboring 75 CDS was found as being the largest, while the shortest was the GI-23 of 4.12 kb in size with just three CDS (Table S4). The GIs of *P. c*. subsp. *odoriferum* (Table S5) ranged between 4.13 kb with 3 CDS (GI-17) and 72.62 with 39 CDS (GI-21). The GIs of the kiwi phytopathogen *P. c.* subsp. *actinidiae* (Table S6) ranged from 70.58 kb with 57 CDS (GI-16) to 4.23 kb with six CDS (GI-12). *P. aroidearum* exhibited the GIs with the smallest sizes among the species (Table S7), being the largest GI-20 with 43 CDS and 46.07 kb, whereas the shortest was GI-7 of 4.01 kb with just three CDS. The genome of *P. parmentieri* was predicted to harbor GIs from 4.03 kb (GI-28 with six CDS) to 82.93 kb (GI-15 with 81 CDS) (Table S8). *P. wasabiae*, with the highest number of predicted GIs, has the largest GI of 91.27 kb with 84 CDS (GI-39) and the smallest GI-6 with five CDS of 4.22 kb in size (Table S9). The sugar beet bacterial pathogen, *P. betavasculorum*, displayed a large GI of 100.94 kb with 41 CDS, and the shortest GI of 4.18 kb with only eight CDS (Table S10). In the case of *P. polaris*, the shortest GI found was the GI-23 with six CDS with 4 kb length, whereas the GI-9 was identified as the largest with 94 CDS with a size of 103.55 kb (Table S11). The largest GI of 100.96 kb (GI-6) containing 91 CDS and the shortest GI with only two CDS with a length of 4.23 kb (GI-17) were identified for *P. peruviense* (Table S12). *C.* P. maceratum showed GIs that vary in sizes, shortest GI-17 of 4.244 kb with four CDS, and the largest GI-13 of 53.43 kb with 42 CDS (Table S13).

**Figure 7:**
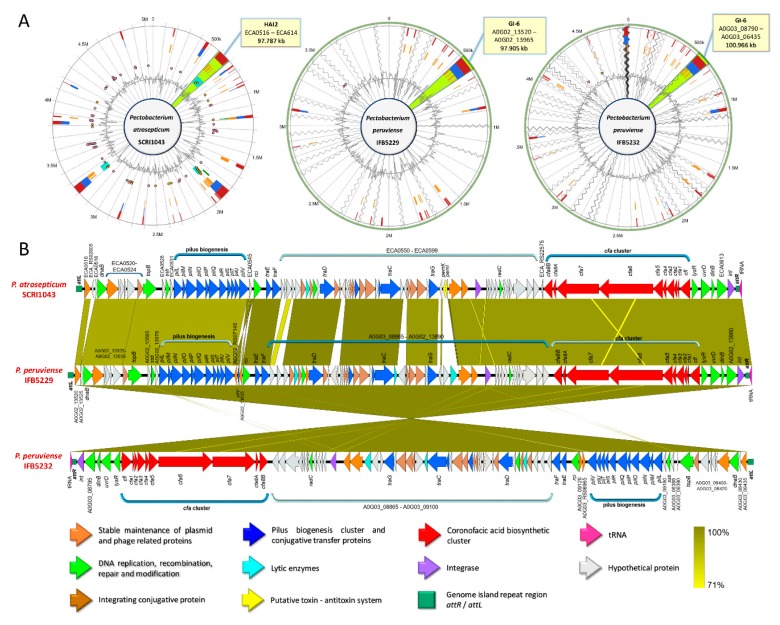
Comparison between the horizontal acquired island 2 (HAI2) of *Pectobacterium atrosepticum* SCRI043 with the genomic island 6 (GI-6) of the newly reclassified species *P. peruviense*. (**A**) Genomic Islands (GIs) plots illustrating the location of HAI2 in *P. atrosepticum* and *P. peruviense*. The plots displayed the predicted GIs based on five approaches (from the inner to the outermost): Islander (turquoise), IslandPick (green), SIGI-HMM (orange), IslandPath-DIMOB (blue), and the integrated methods of the four tools (red). Pink and yellow dots indicated antimicrobial resistance and pathogen-associated genes. The olive green color in each island was manually added to highlight the location of the HAI2 among the three genomes. Moreover, the callout lines in each genome point out the approximate length size of HAI2. (**B**) Linear genomic representation of HAI2 in the three genomes. The arrows depict the genome organization within each island. In addition, the orientation of the arrows indicated forward or reversed the position of each gene. Arrows Color highlighted different genetic functions within the island as well as gene clusters. Dark yellow shaded regions represent areas with high identity between the analyzed genomes with cut-off values of 100% and 71%.

By comparing the GIs across the *Pectobacterium* species, high synteny between the HAI2 of *P. atrosepitucm* and the GI-6 of *P. peruviense* was observed (Figure 7). The HAI2 in *P. peruviense* was delineated, like *P. atrosepticum*, by the direct repeats *attR* and *attL* (green squares in Figure 7B). The organization between both GIs was also highly conserved (Figure 7B). The pilus biogenesis cluster (blue arrows in the figure), as well as the coronofacic acid (*cfa*) cluster (red arrows in the figure), exhibited high homology between both species. Other similarities that were observed in both islands were: genes related to the stable maintenance of plasmids and phage proteins (orange arrows in Figure 7B), a tRNA gene (pink arrow in Figure 7B) located next to *attR*, an integrase (violet arrow), genes that are involved in conjugal integration (brown arrow) and coding regions related to DNA replication, repair, recombination, and modification (green arrows). The *pemK* and *pemI* genes (yellow arrows in the graphic), which encode for a toxin and an antitoxin protein, were only found in *P. atrosepticum*. Additionally, we distinguished some genes encoding lytic enzymes (light blue arrows), as well as some hypothetical proteins, were absent in *P. atrosepticum.* The entire GI-6 of *P. peruviense* IFB5232 was arranged in reverse orientation. We extended the comparison of HAI2 of *P. atrosepticum* to the rest of *P. peruviense* strains available in NCBI GenBank database—a highly similar HAI2 was found in *P. peruviense* strain IFB5929, which displayed a positive orientation contrast to strain IFB5232—since our previous analysis (ANI, dDDH, phylogenetic, codon usage) indicated that *P. atrospeticum* and *P. peruviense* are closely related species (Figure 7B). However, the other two *P. peruviense* strains A97-S13-F16 and A350-S18-N16, isolated from the Durance river stream in France [64], lacked the entire HAI2.

### 3.6. Genome-Wide Comparison of Gene Clusters Associated with Pathogenesis

Several pathogenicity determinants have been described in some species of *Pectobacterium*, including the six type secretion systems (TSS), the plant cell wall degrading enzymes (PCWDE), synthesis of polysaccarides (enterobacterial common antigen, capsular polysaccharide, lipopolysaccharides, exopolysaccharides, and O-antigen), iron acquisition systems, secretion of phytotoxins (coronofacic acid and syringomycin), bacterial attachment operons (type IV pili or fimbriae), secretion of effector proteins (DspE, HrpK, Hrp, Vgr), a necrosis/inducing protein (Nip), an avirulence protein (Svx) similar to *Xanthomonas*, and the 3-hydroxy-2-butanone pathway (3H2B) [2,26,27,65]. We have compared the similarities, divergences, or absence of the virulent determinants among all *Pectobacterium* species. 

**Figure 8:**
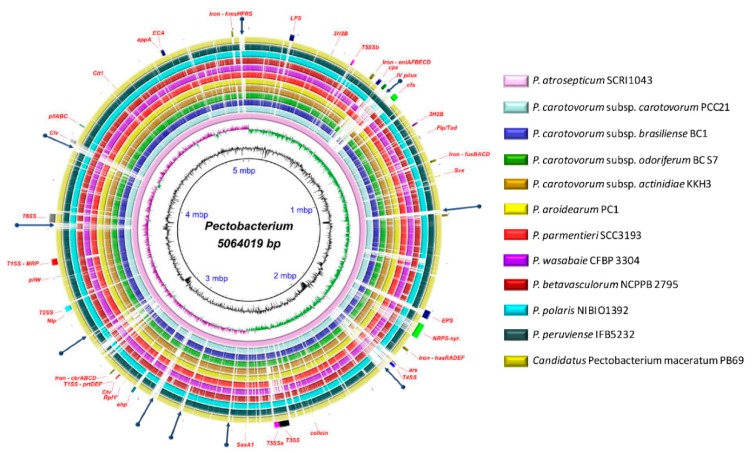
Genomes comparison across the *Pectobacterium* genus. The BLAST ring image generated using BRIGS displayed the genome comparison of 12 genomes within genus *Pectobacterium*. From the innermost origin of each lane, the image features were characterized: the axis with the size of genome (kbp), the GC content (black line), the GC skew (purple color indicates G’s), and afterward each color ring depicting each *Pectobacterium* species genome, namely, *P. atrosepticum* SCRI1043 (NC_004547), *P. carotovorum* subsp. *carotovorum* PCC21 (NC_018525), *P. c*. subs. *brasiliense* BC1 (NZ_CP009769), *P. c.* subsp. *odoriferum* BC S7 (CP009678), *P. c.* subsp. *actinidiae* KKH3 (NZ_JRMH00000000), *P. aroidearum* PC1 (NC_012917), *P. parmentieri* SCC3193 (NC_017845), *P. wasabiae* CFBP 3304 (NZ_CP015750), *P. betavasculorum* NCPPB2795 (NZ_JQHM00000000), *P. polaris* NIBIO1392 (NZ_CP017482), *P. peruviense* IFB5232 (NZ_LXFV00000000), and *Candidatus* Pectobacterium maceratum (NZ_PDVY00000000). The outermost ring identified the different loci of most relevant pathogenicity determinants and antimicrobial biosynthetic clusters. The complete genome of *P. atrospeticum* served as a template for the generation of the BRIG image based upon the nucleotide BLAST analysis. Blue arrows highlighted genomic regions that exhibited divergences when compared to the reference genome. Key acronyms (red legends) used to name the clusters or single virulence genes in the outermost ring are detailed: T1SS-MRP (Type I secretion system—multi-repeat protein), T1SS-*prtDEF* (Type I secretion system—protein transporters PrtDEF), T2SS (Type II secretion system), T3SS (Type III secretion system), T4SS (Type IV secretion system), T5SSa (Type V secretion system-*hecAB*), T5SSb (Unreported type V secretion system—described in this study), LPS (lipopolysaccharide), *cps* (capsular polysaccharide), ECA (Enterobacteria Common Antigen), EPS (Exopolysaccharide O-antigen), Iron-*hmsHFRS* (hemin storage), Iron- *entAFECD* (enterobactin), Iron-*fusBACD* (ferredoxin uptake), Iron-*hasRADEF* (heme acquisition), Iron-*cbrABCD* (achromobactin), 3H2B (3-hydroxy-2-butanone pathway), Flp/Tad (Fimbrial low-molecular-weight protein/Tight adherence protein), IV pilus (Type IV pilus), *pilW* (Pilus assembly protein PilW), *pilABC* (pilus production proteins PilABC), *cfa* (coronofacic acid), NRPS-*syr* (Non-Ribosomal Peptide-Synthetase like syringomycin), *ars* (arsenic resistance), Ctv (carotovoricin), *ehp* (phenazine antibiotic biosynthesis), colicin (colicin-like bacteriocin), Svx (virulence protein homologue to *Xanthomonas campestris*), SaxA1 (aliphatic isothiocyanate resistance protein SaxA), RplY (ribosomal protein RplY), Cit1 (citrate transporter), and Nip (Necrosis-inducing protein), and *appA* (phytase).

The antimicrobial compounds, such as carotovoricin [31,66,67], carbapenem [28], phenazine [21], and colicins-like bacteriocins [29,30,68,69], which were identified in some *Pectobacterium* species to out-compete and attack the other microbes for food and specific niche, were also analyzed. A BLAST ring image (Figure 8) displayed nucleotide comparison among 12 species—the outer arrows highlight the locus of the above-described virulent determinants and the antimicrobial components. *P. atrosepticum* SCRI1043 was used as a reference.

### 3.7. Type Secretion Systems

The type I secretion system (T1SS) secrets hydrolases, toxins, nodulation factors, adhesins, and hemophores from the cytoplasm to the extracellular media, and it consists of three elemental proteins, including a specific outer membrane protein (OMP) or TolC, an ATP-binding protein, and a membrane fusion protein (MFP) [70]. In *Pectobacterium*, three different T1SS have been described [65]. The first one is formed by the operon prtD, prtE, and prtF, and it allows the secretion of metalloproteases (prtW) [27,65]. In our analysis, the prtDEF operon shown to be highly conserved in all the species (Table S14). Likewise, the metalloprotease prtW was also observed in all genomes (Table S16). The second T1SS, involved in secretion of hemophore HasA homologue to T1SS-HasA of *Serratia marcescens* [21,65], was absent in *P. betavasculorum* and closely related *P. parmentieri* and *P. wasabiae* (Table S30). In the analysis, the entire cluster of the third T1SS, involved in secretion of a proteinaceous multi-repeat adhesin protein (MRP) [71], was observed among all species and exhibited high similarity (Table S14 and Figure S5). However, the gene encoding the multi-repeat adhesin protein (MRP) displayed a huge divergence in size among the *Pectobacterium* species (Figure S5). For instance, the lengths of the MRP in *P. parmentieri* and *P. peruviense* were 4,173- and 3,829-bp, respectively; however, the largest MRP-encoding gene was observed in *P. polaris* (15,240 bp) and *P. c.* subsp. *carotovorum* (14,613 bp) (Table S14). Moreover, *P. c.* subsp. *odoriferum* harbor MRP and LapG proteins as pseudogenes—indicating that this species might lack the functionality of MRP/T1SS. 

The type II secretion system (T2SS) or Out system constitutes a two-step process secretion machinery, which secretes the proteins from cytoplasm to extracellular medium, composed with a set of 12 to 16 genes denominated as the *out* gene cluster [72,73]. The *out* operon was highly conserved across all *Pectobacterium* species (Table S15 and Figure S6). However, the *outN* gene was missing in *P. parmentieri* and *P. wasabiae* (Figure S5). Likewise, neither *P. parmentieri* nor *P. wasabiae* harbored a pectate lyase located close to the T2SS cluster (Figure S6). Based on the literature [8,21,23,26], we have predicted that all of the Plant cell wall degrading enzymes (PCWDE) present in *Pectobacterium* species (Table S16). In the category of pectate lyases, 11 enzymes were found; among these, the HrpK protein with a pectate lyase domain was absent in both *P. parmentieri* and *P. wasabiae*. Moreover, the periplasmic pectate lyase was not found in *P. betavasculorum*. In regards to pectin lyase (*pnl*), pectin acetylesterases (*paeX* and *paeY*) and pectin methylesterases (*pemA* and *pemB*) were present in all species. In contrast, a putative polygalacturonase, annotated as “glycoside hydrolase family 28”, was only present in *P. aroidearum*. The polygalacturonase PehK was not observed in *P. parmentieri* and *P. wasabiae*, while the rest of polygalacturonases (*pehA*, *pehN*, and *pehX*) were present in all 12 species. In the case of cellulases, the glycoside hydrolase (*celB*) was not present in *P. c.* subsp. *brasiliense* and *P. polaris*, whereas the *celV* and *bcsZ* genes were highly conserved across the *Pectobacterium* species. The oligogalacturonide liase (*ogl*) and Rhamnogalacturonate lyase (*rhiE*) were also present in all *Pectobacterium* species. Proteases are fundamental pieces in bacterial virulence and they are secreted through the *prtDEF* operon; these group of proteins exhibited the highest difference when compared with PCWDE (Table S16). While some proteases, such as metalloproteases PrtW and Prt1, peptidases M3, M20, and S58, deacetylase, D-aminopeptidase, tripeptide aminopeptidase, and zinc dependent protease, were common among the 12 species, the other proteases, such as peptidase M50 and peptidase S9 prolyl oligopeptidase, were solely restricted to *P. aroidearum*. Similarly, Omptin family outer membrane protease 7 and serine protease were only found in *P. parmentieri* and *P. wasabiae*. Other proteins, including a peptidase S53, a putative zinc protease (*pqqL*), and an acetylornithine deacetylase were only absent in *P. betavasculorum*. The staphylolytic protease, LasA, was not present in *P. parmentieri* and *P. wasabiae*. 

**Figure 9:**
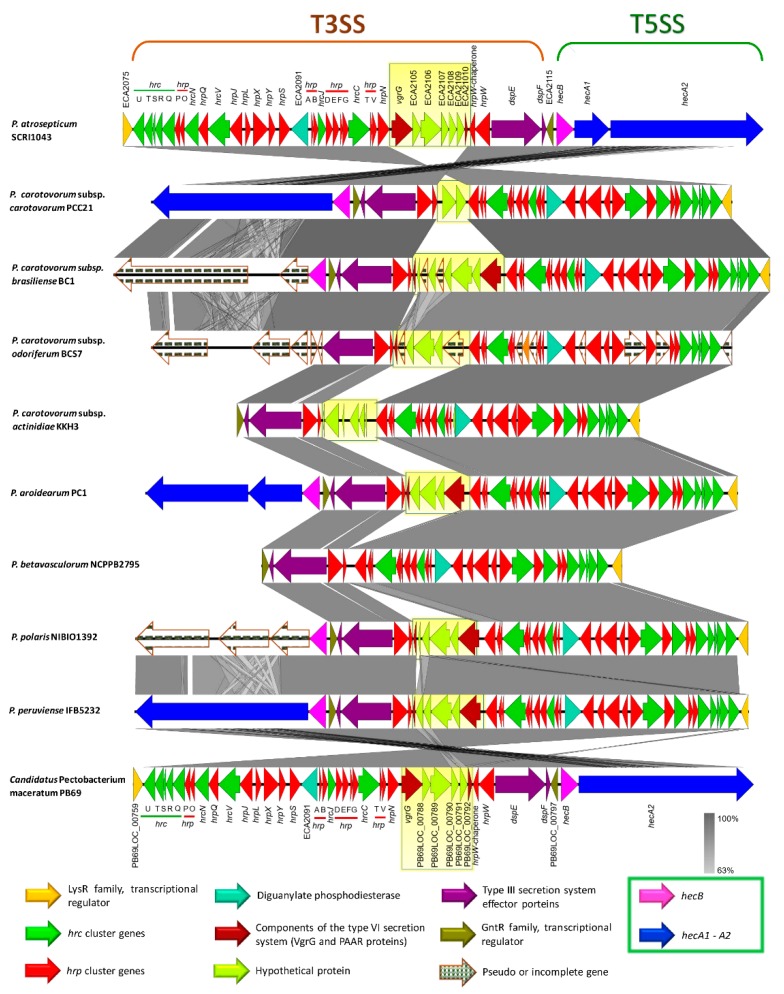
Comparison of the genetic organization of type III secretion system (T3SS) among the *Pectobacterium* species. The arrow position represented forward/reverse gene orientation. Arrow color signified specific gene composition within the T3SS. Gene names were provided at the top and bottom of the linear graph; the locus-tag was used for hypothetical or genes with no names. A pairwise alignment between the linear sequences was rendered based upon BLAST algorithm with cut-off values from 64% to 100%. Regions with higher nucleotide identity were displayed with a shaded grey. A light-yellow blur square highlighted the variable region among the genomes that interrupted the type III secretion gene cluster. Expanded legend entry acronyms are provided: *hrc* (hypersensitive response and pathogenicity conserved genes), *hrp* (hypersensitive response and pathogenicity or hairpin proteins), VgrG (valine-glycine repeat protein G), PAAR (proline-alanine-alanine-arginine repeat protein), *dspE* (disease-specific effector protein E), *dspF* (disease-specific chaperone protein F), *hecB* (hemolysin activation protein HecB), and *hecA* (hemolysin/hemagglutinin-like protein HecA).

The type III secretion system (T3SS) is the most extensively studied bacterial pathogen system. This system forms injection machinery that allows close interaction of bacteria with the eukaryotic hosts [2,27]. It is reported that the T3SS cluster is present in *P. atrosepticum* SCRI1043 and harbored within the genomic island HAI7. Within the T3SS, a genetic region between the *hrpN* and *hrpW* genes (Table S17, marked in red bold) displayed a high discrepancy among the species (yellow blur rectangle in Figure 9), we named this section as a variable region. *P. betavasculorum*, for instance, did not have this region. This variable region harbored two components of type VI secretion system: a valine glycine repeats G protein (VgrG) and a proline-alanine-alanine-arginine repeat protein (PAAR), as presented in some species. No remnant of T3SS was found in *P. parmentieri* and *P. wasabiae*. 

Contrary to the other secretion systems, the type IV secretion system (T4SS) is described as the most versatile delivery nanomachine that can translocate diverse macromolecules, such as DNA, monomeric substrates, and multimeric proteins, to either bacterial or eukaryotic target cells, usually through a direct cell-to-cell contact [74,75]. The virB-T4SS homologs to *Agrobacterium tumefaciens* were found in *P. c.* subsp. *brasiliense* BC1, *P. c.* subsp. *actinidiae* KKH3, and *P. betavasculorum* NCPPB2795 (Table S18 and Figure S7). Intriguingly, we found two virB-T4SS clusters in *P. c.* subsp. *brasiliense* BC1, and the genetic features of both the clusters were highly similar (Table S18). We investigated the other two strains of *P. brasiliense*: SX309 and BZA12, since we found two T4SS clusters in *P. c.* subsp. *brasiliense*; however, two copies of this cluster were not detected. 

The type V secretion system (T5SS) is defined as the simplest bacterial secretion apparatus and classified into five categories, from Va to Ve [65,76]. Soft rot enterobacteria harbor the type Vb, known as two-partner secretion system or Tps, encoded by the *hecA* and *hecB* genes [65]. We found *hecAB* operon in *P. c.* subsp. *carotovorum*, *P. c.* subsp. *brasiliense*, *P. c.* subsp. *actinidiae*, *P. aroidearum*, *P. polaris*, *P. peruviense*, and *C.* P. maceratum (Table S19). Additionally, the two-partner secretion (Tps) system cluster in the species mentioned above lies adjacent to T3SS, except for *P. c.* subsp. *actinidiae* (Figure 9). This cluster was not found in *P. parmentieri*, *P. wasabiae*, and *P. betavasculorum*, whereas the *hecA1* gene was not present in *P. c.* subsp. *carotovorum*, *P. c.* subsp. *actinidiae*, *P. peruviense*, and *C.* P. maceratum (Table S19 and Figure 9). Moreover, the entire system was conformed by pseudogenes in *P. c*. subsp. *odoriferum*. In *P. c.* subsp. *brasiliense* and *P. polaris*, the hemagglutinin-coding loci (HecA) were found as pseudogenes. An additional cluster was also found, seems to be homologous to *hecAB*. However, the genetic content of this cluster was different; it contained a toxin-activating lysine-acyltransferase locus instead of two *hecA* genes (Table S19 and Figure S8). The species *P. wasabiae* and *P. peruviense* lack this operon. Likewise, the locus encoded for toxin-activating lysine-acyltransferase was not present in *P. atrosepticum* and *P. parmentieri*, while in *P. c*. subsp. *odoriferum*, this gene and gene encoded a filamentous hemagglutinin were present as pseudogenes. The Tps cluster, like *hecAB*, which was found in some species resembled a CDI system of *P. parmentieri* SCC3193 within the GI_5 [26] (Table S19).

The type VI secretion system (T6SS), described as an injectosome like weapon structure that resembles to a bacteriophage tail [77], was found in all 12 *Pectobacterium* species (Table S20 and Figure S9); however, we observed genomic discrepancy; for instance, *P. c.* susbp. *odoriferum* harbor an extra set of genes, predicted hypothetical proteins, between *hcp* and *vasL* (virulence-associated protein L) genes (Table S20). Furthermore, a part of the T6SS cluster reported in *P. atrosepticum* (ECA3420–ECA3428) was completely absent in sugar beet pathogen, *P. betavasculorum* NCPPB2795 (Figure S9). In *P. parmentieri* and *P. wasabiae*, the previously mentioned loci, ECA3420–ECA3428, was located to a different place in the chromosomes (Table S20). Besides, ECA3420 locus and Rhs protein were not present in *P. c.* subsp. *carotovorum* PCC21. Likewise, the locus ECA3425 was not identified in *P. c.* susbp. *odoriferum*, *P. c.* subsp. *actinidiae*, *P. aroidearum*, *P. polaris*, *P. peruviense*, and *C.* P. maceratum. Moreover, the second T6SS cluster identified by Nykyri et al. [26] within a genomic island in *P. parmentieri* (GI_30) and *P. wasabiae* was not found in any other species included in this study. The haemolysin-coregulated proteins (Hcp) and valine-glycine repeat proteins (VgrG) have been reported as the major effector proteins injected from this system to the target cells [78,79]. PAAR proteins were later attributed as key components of the T6SS of *S. marcescens* [80]. Mattinen et al. [81] described the presence of three copies of *hcp* gene and four copies of the *vgrG* gene in *P. atrosepticum* that were shown to be similar to the genes present in main T6SS cluster. In the case of *P. parmentieri* SCC3193, 26 copies of either *hcp* or *vrgG* genes were detected [26]. In the current analysis, we also found several copies of haemolysin-coregulated proteins (Hcp) and valine-glycine repeat proteins (VgrG), the major effector proteins, harbored in genomic islands (GIs), but scattered throughout the genomes (Tables S2–S13). Furthermore, we have described that the T3SS cluster possesses a variable region contained two components of T6SS: VgrG and PAAR proteins, which were shared in some species (Table S17 and Figure 9).

### 3.8. Pilin Clusters and Attachment Mechanisms

Pili or fimbriae are appendages and flexible filaments that extend through the Gram-negative bacterial surface and linked to main virulence functions [82]. We identified identical type IV pili clusters in *P. peruviense* IFB5232, while the rest of the species lacked this operon (Table S21 and Figure S10). Furthermore, like *P. atrosepticum* and *P. parmentieri*, the *pil* operon in *P. peruviense* was harbored in a genomic island (GI-6). However, the function of this cluster to adhesion has not been identified in *Pectobacterium.* Additionally, we also found a *pilW* gene, encoded for a “type IV pilus biogenesis and stability protein,” was highly conserved across the *Pectobacterium* species (Table S21). Aside from the locus *pilW*, another operon consisted of three genes *ppdD*, *hofB*, and *hofC* was predicted in all species (Table S21). The functionality of both *pilW* and the gene cluster *pilABC* is still to be deciphered in *Pectobacterium*. The Flp/Tad (fimbrial low-molecular-weight protein–tight adherence protein) cluster and the two-component regulators (response regulator–sensor kinase), located upstream of the *flp* locus, were identified in all 12 species (Table S22 and Figure S11). 

### 3.9. Production of Phytotoxins

The secretion of phytotoxins constitutes another pathogenicity determinant, and they are maintained within the arsenal of pathogenic bacteria to invade, disrupt, and colonize plant tissue [27]. We found that *P. peruviense* IFB5232 possesses a *cfa* cluster, a gene that is responsible for the biosynthesis of coronofacic acid—resembling the one reported in *P. atroseptium*. Moreover, this polyketide phytotoxin cluster was also observed in *P. c.* subsp. *actinidiae* and *P. betavasculorum* (Table S23 and Figure S12). Like *P. atrosepticum* SCRI1043 [21], the *cfa* operon in *P. c.* subsp. *actinidiae*, *P. betavasculorum*, and *P. peruviense* appeared to be acquired through horizontal gene transfer, since this cluster was identified within the predicted genomic islands GI-6, GI-5 and GI-14, respectively. On the other hand, among all species, only *P. c.* subsp. *actinidiae* exhibited a similar NRPS system, like syringomycin, which was predicted to be the part of GI-9. In addition, only three genes (KKH3_14020, KKH3_14030, and KKH3_14040) were identified as the main core of NRPS system in *P. actinidiae*, while other genes, reported in the HAI6 of *P. atrosepticum*, were absent (Table S24). The locus KKH3_14040 predicted to encode a siderophore biosynthesis NRPS, displayed the highest differences with a nucleotide identity of 78.5% and query cover of 85.8% when compared with NRPS syringomycin of *P. atrosepticum* (Table S24). So far, NRPS-like syringomycin function and role in virulence have not yet been demonstrated. Therefore, whether this NRPS system in *P. actinidiae* contributes to the development of canker disease in kiwi fruits, remains uncertain.

### 3.10. Arsenic Resistance is an Intrinsic Feature of Few Species

Arsenic clusters (*ars*) confer bacteria the ability to resist against the concentrations of inorganic arsenic present in an environment [83]. We observed that *P. c.* subsp. *brasiliense*, *P. peruviense*, and *C.* P. maceratum harbored orthologues to *arsC*, *arsB*, and *arsR* genes, which displayed more similarity to *P. atrosepticum*, by comparing the *ars* cluster of *P. parmentieri* and *P. atrosepticum* with other species (Table S25 and Figure S13). *P. c.* subsp. *carotovorum* only contained one gene (*arsC*, PCC21_022310). Another copy of *arsC* in *P. parmentieri* (W5S_3181), was observed to be present in all of the species (Table S25). Additionally, we found another gene (ECA2265) in *P. atrosepticum*—annotated as arsenic resistance protein (*arsH*). The mentioned locus showed high similarity among all species (Table S25).

### 3.11. Polysaccharide Clusters

Most of the bacteria produce cell surface polysaccharides, reported to promote bacterial attachment inside the host, protect bacterial cells from adverse environmental conditions and plant toxins, and enhance colonization and virulence [27]. In our analysis, we observed that the capsular polysaccharide cluster (*cps*) was highly conserved in eight species (Table S26 and Figure S14). However, the entire cluster was not found in *P. c.* subsp. *brasiliense*, *P. c.* subsp. *actinidiae*, *P. aroidearum*, and *P. betavasculorum*. The enterobacterial common antigen (ECA) that was composed of 11 genes (*wec* and *rff*) was present in all species (Table S27 and Figure S15). The EPS and O-antigen cluster (*wza* and *rfb*), as documented previously in the HAI5 of *P. atrosepticum* [21], was only conserved partially across the species (Table S28 and Figure S16). Only the first 4 and last 3 loci were observed to be present in all species. The lipo-oligo/polysaccharide cluster (LOS/LPS) displayed the highest differences among species—with different rearrangements, only five genes found in all species (*rfaD*, *waaF*, *waaC*, *waaA*, and *coaD*) (Table S29 and Figure S17).

### 3.12. Different Iron Piracy Strategies

Iron is a key micronutrient in the bacterial life, as it participates in the cellular signaling, and is a cofactor of many enzymes [27,84]. The siderophore achromobactin, which is composed of the operon *cbrABCD*, was highly conserved across all species (Table S30). In contrast, the operon *entAFBECD*, forms the enterobactin siderophore, was not complete in all species. For instance, the locus ECA0476, encodes for a putative iron transporter, and the genes *entF* and *entB* were not present in *P. wasabiae* and *P. parmentieri*. In addition, both species presented a locus, annotated as a major facilitator superfamily (MFS), was not found in other species (Table S30). The ferric citrate uptake system (*fecIRABCDE*) was not carried by *P. c.* subsp. *odoriferum*, *P. wasabiae*, *P. parmentieri*, and *P. betavasculorum*. Moreover, *P. aroidearum* PC1 only exhibited a part of this cluster with missing genes of *fecA*, *fecR*, and *fecI*. The cluster *hasRADEF*, resembled to hemophore of *Serratia marcesens* [21], was not observed in *P. betavasculorum*, whereas only *hasR* and the locus—encodes for an iron siderophore sensor protein—were found in *P. parmentieri* and *P. wasabiae*. The operon *hmsHFRS*, which is involved in the hemin storage similar to *Yersinia pestis*, was harbored by five species: *P. atrosepticum*, *P. aroidearum*, *P. parmentieri*, *P. wasabiae*, and *P. peruviense.* Except for *P. betavasculorum*, all of the species appeared to harbor the *fusABCD* operon involved in the plant-ferredoxin uptake system (Table S30). Lastly, the aerobactin siderophore cluster (*iuc*), as reported by Nikiry et al. [26] in *P. parmentieri* SCC3193 and previously found in *P. c.* subsp. *carotovorum* W3C105 [85], was not present in all species. Besides, as Nikiry et al. [26] described that aerobactin cluster was present in *P. parmentieri* WPP163, we compared this cluster among other *P. parmentieri* strains (RNS 08-42-1A, PB20, SS90, CFIA1002)—the cluster was highly conserved across all *P. parmentieri* strains (Table S31).

### 3.13. Other Crucial Virulence Determinants

Other single virulence genes were also compared among the *Pectobacterium* species (Table S32). The *svx* gene homologous to the *avrXca* of *Xanthomonas campestris* and a putative virulence factor in *P. atrosepticum* SCRI1043 [86] were highly conserved across all 12 species. The necrosis inducing virulence protein (Nip), where virulence was demonstrated in *P. parmentieri* SCC3193 and *P. atrosepticum* SCRI1043 [87], was also present in the rest of the 10 species. The citrate transporter encoded by the *cit1* gene, which was observed to reduce the citrate levels and enhanced the growth of *P. atrosepticum* on potato [88], was also identified in all species. The phytase gene *appA* formerly identified in *P. wasabiae* [89]—possible role in pathogenicity has not been revealed yet—appeared to be encoded by all species, except *P. c.* subsp. *odoriferum*. Welte et al. [90] found SaxA enzyme involved in isothiocyanates breakdown, was already observed in *P. c.* subsp. *carotovorum*, *P. c.* subsp. *brasiliense*, *P. c.* subsp. *odoriferum*, and *P. atrosepticum.* In our analysis, we found the *sax* gene in *P. polaris*, *P. peruviense*, and *C.* P. maceratum. Furthermore, all of the species were predicted to maintain a copy of the *rply* gene, which encodes for the ribosomal protein RplY, a verified determinant for full virulence in *P. carotovorum* PccS1, the expression of which is induced by plant extract [91]. Among all species, the *Erwinia* virulence factor (evf), involved in enhancing interactions of certain *P. carotovorum* strains with *Drosohila melanogaster* [9], was only present in *C.* P. maceratum PB69 (PB69LOC_02095). The *bud* operon, encoded for acetoin or 3-hydroxy-2-butanone pathway (3H2B) [92], was conserved among all species; however, the *budC* gene was located far away from *budRAB* operon (Table S33 and Figure S14).

The 50 flagella genes were conserved across all species: *P. atrosepticum* SCRI1043 (ECA1685–ECA1740), *P. parmentieri* (W5S_1760–W5S_1810), *P. c.* subsp. *carotovorum* PCC21 (PCC21_026650–PCC21_027210), *P. c.* subsp. *brasiliense* BC1 (NC16_13535–NC16_13255), *P. c.* subsp. *odoriferum* BC S7 (BCS7_13110–BCS7_12835), *P. c.* subsp. *actinidiae* KKH3 (KKH3_25780–KKH3_25130), *P. aroidearum* PC1 (PC1_2615–PC1_2560), *P. wasabiae* CFBP 3304 (A7983_22275–A7083_22035), *P. betavasculorum* NCPPB2795 (KP22_07745–KP22_07480), *P. polaris* NIBIO1392 (BJK05_08375–BJK05_08100), *P. peruviense* IFB5232 (A0G03_04140–A0G03_03865), and *C.* P. maceratum PB69 (PB69LOC_00365–PB69LOC_00421). In all bacterial species, the flagellar genes were located within genomic islands (Tables S2–S13), which indicate that the common ancestor of all *Pectobacterium* species acquired the set of flagellar genes through horizontal gene transfer. 

### 3.14. Virulence Mechanism in Pectobacterium Species Appears to be Regulated by Several Factors

The study of regulatory mechanisms in *Pectobacterium* has revealed that specific regulators that modulate transcription positively or negatively mediate the function of pathogenicity determinants; at the same time, these regulators are activated or suppressed by the other molecules, thus constituting an extensive complex network of regulators [27,65]. Overall, all of the proteins involved in QS (quorum sensisng: ExpI, ExpR1, VirR/ExpR2, CarR/ExpR3, LuxS), global regulators (RsmA, KdgR, ExpM, Hor, and HexY), two-component regulators (PehR-PehS, PmrA-PmrB, and ExpS-ExpA), phosphorelay system (RcsC, RcsD, and RcsB), and other regulatory compounds (HexA and RexZ) were almost conserved in all 12 *Pectobacterium* species (Table S34), with a few exceptions, including the presence of CarR (ExpR3) in only those carbapenem-producing strains [93], the lack of VirS in *P. parmentieri* SCC3193, and the absence of PmrA-PmrB, and *rexZ* in *P. betavasculorum*. The response virulence regulator (*evr*), formerly described in *P. atrosepticum* [8], was found only in *P. c.* subsp. *carotorum* PCC21. Furthermore, the Fur regulator, which controls ferric uptake [27], was conserved across all species. All of the species carried SirB1, which is necessary for virulence in *P. parmentieri* SCC3193 [26], in their genomes. Finally, the Cytidine repressor (CytR) of *P. aroidearum* PC1, which controls biofilm formation [94], was predicted in all 12 *Pectobacterium* species. 

### 3.15. Diverse Antimicrobial Compounds Secreted by Pectobacterium Species

The mining of *Pectobacterium* species genomes has revealed several genes that are involved in the synthesis of secondary metabolites with antimicrobial function. Based on this information, our objective was to analyze all *Pectobacterium* species for the presence or absence of the previouly mentioned antimicrobial compounds. 

Carotovoricin synthesis—Carotovoricin (Ctv) has been defined as a phage tail bacteriocin of high molecular weight [31,67]. All *Pectobacterium* species possess the Ctv cluster (Table S35) adjacent to the T1SS (Figure S19). However, the genes *fibA* and *fibB*, encoded for a bacteriophage tail fiber, were missing in *P. c.* subsp. *actinidiae*, and *P. betavasculorum*. Likewise, the ferredoxin (2Fe-2S) protein was not identified in *P. atrosepticum*, *P. c.* subsp. *brasiliense*, *P. parmentieri* and *P. wasabiae*. Moreover, in *P. c.* subsp. *carotovorum*, *P. c.* subsp. *odoriferum* and *P. polaris*, the *fibB* gene was not located within the *ctv* operon but present at another region (Table S35). Additionally, the organization of the *ctv* gene cluster in *P. atrosepticum* and *P. betavasculorum* showed divergence as compared with other species. Specifically, 14 genes in *P. atrosepticum* and 17 genes in *P. betavasculorum* located far away from first part of the *ctv* cluster (yellow blur rectangle in Figure S19) and in reverse orientation. The *ctv* cluster of *P. betavasculorum* displayed the highest divergence among the species, with three hypothetical genes only found in this bacterium (Figure S19). 

Carbapenem synthesis—First time *P. c.* subsp. *carotovorum* ATTC 39048 was described as a carbapenem producer among *Pectobacterium* species [95]. This cluster is composed of an operon of eight genes, denotated as *carABCDEFGH*, with *carA*, *carB*, *carC*, *carD*, and *CarE*, assigned as crucial encoding enzymes genes, whose function is the biosynthesis of carbapenem, while the *carF* and *carG* genes were observed to encode intrinsic elements related to resistance against the carbapenem and *carH* with an unknown function [28,96]. Only *P. c.* subsp. *actinidiae* and *P. betavasculorum* contained the complete *car* operon (Table S36). We cross checked with other strains of both species, and all of them also maintained identical carbapenem genes, thus showing that carbapenem synthesis seems to be an innate characteristic of kiwi and sugar beet bacterial pathogens (Figure S20). We also included the carbapenem cluster of *P. c.* subsp. *brasiliense* strains 1692 and ICMP 19477, displayed high similarity with other carbapenem producing species and with *P. c.* subsp. *carotovorum* ATCC 3908. Surprisingly, *P. c.* subsp. *brasiliense* BC1, *P. parmentieri*, and *P. wasabiae* only possess partial *car* operon—*carF*, *carG*, and *carH* (Table S36 and Figure S20).

Phenazine synthesis—It has been described that phenazines activity triggers an excessive accumulation of reactive oxygen species, which affect cellular redox and end up inhibiting the cell growth [97]. All the strains of both *P. c.* subsp. *actinidiae* and *P. betavasculorum* carried phenazine biosynthetic cluster, as previously observed in *P. atrosepticum* strains SCRI1043 and ICMP1526, and *P. c.* subsp *brasiliense* ICMP19477 (Table S37 and Figure S21). However, the size of the *ehpG* varied—1218 bp in *P. c.* subsp. *brasiliense* and *P. betavasculorum*, 1410 bp in *P. atrosepticum* SCRI1043 and all *P. c.* subsp. *actinidiae* strains, and 1383 bp in *P. atrosepticum* ICMP 1526. This gene encodes one of the key enzymes involved in the biosynthesis of phenazine.

Colicin-like bacteriocins—Colicin-like bacteriocins exhibited powerful toxic effects against closely related bacteria [98]. We found few uncharacterized bacteriocins in few *Pectobacterium* species (Table S38). For instance, homologous to the Carocin D [69] were observed in *P. c.* susbp. *brasiliense*, *P. c.* subsp. *odoriferum*, and *P. betavasculorum*. The previous characterized pectocin M1 [98], in *P. aroidearum*, was also predicted in *P. carotovorum* subsp. *carotovorum* PCC21. A type of colicin/pyocin-like bacteriocins were also identified in three *P. carotovorum* subspecies (*carotovorum*, *brasiliense*, and *odoriferum*) and *P. wasabiae*. Likewise, a colicin like-bacteriocin protein, and its corresponding immunity protein, were found in *P. atrosepticum* SCRI1043. The rest of the species, *P. parmentieri*, *P. c.* subsp. *actinidiae*, *P. peruviense*, *P. polaris*, and *C.* P. maceratum, apparently did not carry carocin-like bacteriocin operons.

### 3.16. A unique Phosphotransferase System (PTS) Cluster Conserved in P. parmentieri

A 4.5 kb region, which contained five genes, was exclusively present in *P. parmentieri* SCC3193. We compared this region with other *P. parmentieri* strains available in the NCBI GenBank database, and observed that all strains (WPP163, RNS 08-42-1A, PB20, SS90, and CFIA1002) maintained this highly conserved cluster (Table S39). The cluster corresponds to a cellobiose-specific phosphotransferase system (PTS), which contained five genes (Figure S22). The cluster found in *P. parmentieri*, first three genes encoded for three domains EIIA, EIIB, and EIIC, which together conformed substrate specific permease to transport cellobiose from cell membrane to cytoplasm. The gene next to the EIIC, encoded a beta-glucosidase with cellobiose/specific PTS activity, predicted function—conversion of cellobiose to glucose. The LacI family transcriptional regulator adjacent to this gene is most likely responsible for the regulation of the transcription of cellobiose PTS permease subunits EIIA, B, and C, as well as beta-glucosidase. 

### 3.17. Pectobacterium Species Harbor Different CRISPR-Cas Systems

**Figure 10:**
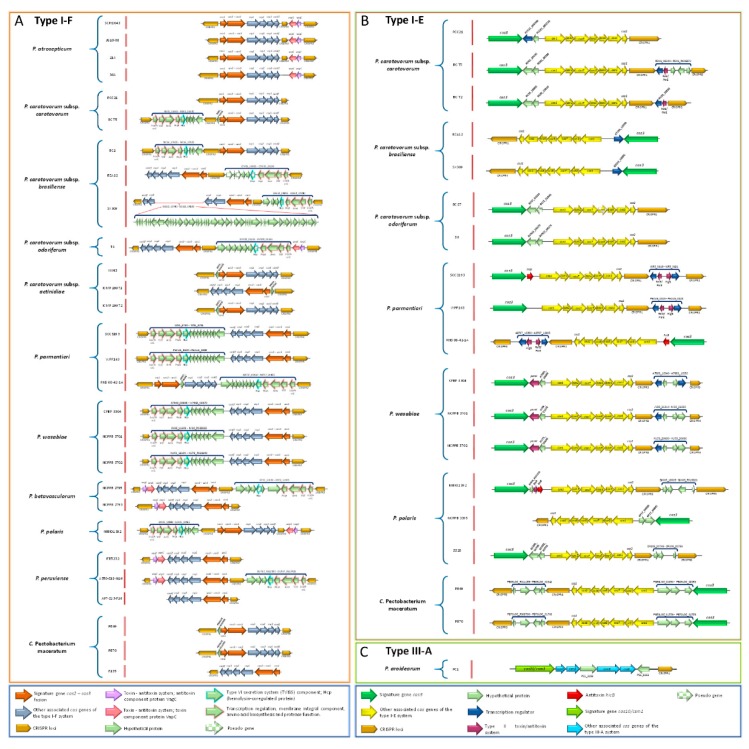
Schematic sequence organization of the type I-F, type I-E, and type III-A clustered regularly interspaced short palindromic repeats-Cas (CRISPR-Cas) across the genomes of the 12 *Pectobacterium* species. (**A**) CRISPR-Cas subtype I-F marked in orange. (**B**) CRISPR-Cas subtype I-E system marked in green. (**C**) CRISPR-Cas type III-A marked in yellow. Brown horizontal pentagons represented the CRISPR loci while the arrows depicted specific gene composition such as the *cas* or *csm* and other surrounding genes with either known or unknown function. The arrow and pentagon positions represented sequence orientation. Descriptions of gene names are provided inside the arrows or pentagons as well as at the top or bottom of each linear graph; genes without a name or hypothetical proteins labeled with locus-tag. The linear graphs were drawn manually and represent the scale of the respective gene lengths. Key acronyms used in the linear sequences: CRISPR (clustered regularly interspaced short palindromic repeats), *cas*, *csy*, and *csm* (genes encoding CRISPR-associated proteins), Lrp/AsnC (leucine responsive regulatory protein/asparagine synthase C transcription regulator family); YitT (membrane protein YitT), Amt (aspartate/tyrosine/aromatic aminotransferase), Pept (M48 family peptidase), Hcp (hemolysin-coregulated protein). VapC (toxin protein), VagC (antitoxin/virulence-associated protein), HigB (mRNA interferase toxin HigB), RelE/ParE (toxin-antitoxin system, mRNA interferase toxin RelE/toxin ParE), HicA (mRNA interferase toxin HicA), HicB (antitoxin HicB), *pemK* (mRNA interferase toxin pemK).

The CRISPR-CAS constitutes the adaptive immune system in bacteria [99]. We found three types of CRISPR-Cas systems across the *Pectobacterium* species: subtype I-F, subtype I-E, and type III-A (Figure 10). All of the strains within species *P. atroseoticum*, *P. c.* subsp. *actinidiae*, *P. betavasculorum*, and *P. peruviense* contained the subtype CRISPR-Cas system I-F (Figure 10A). *P. aroidearum* was the only species harbored type III-A CRISPR-Cas system (Figure 10B). The rest of the species exhibited great heterogeneity, with some strains containing both I-F and type I-E CRISPR-Cas systems, while others, such as *P. c.* subsp. *brasiliense* BC1, *P. c.* susbp. *odoriferum*, *P. polaris* strains NCPPB 3395 and SS28, and *C.* P. maceratum F135 merely presented the subtype I-E CRISPR-Cas (Figure 10C). We also found strains with no CRISPR-Cas systems, namely *P. polaris* NIBIO1006 and *P. peruviense* IFB5229. The presence of putative toxin and antitoxin proteins were observed in subtype I-F CRISPR-Cas systems (Figure 10A). For instance, the analyzed strains in *P. atrosepticum* revealed the presence of VapC toxin and VagC antitoxin between CRISPR3 and CRISPR2. Likewise, same toxin/antitoxin components were observed in *P. c.* subsp. *odoriferum* T4, both strains of *P. betavasculorum*, and in *P. peruviense* IFB5232 and A350-S18-N16. Other species and certain strains contained a set of sequences downstream of *cas6f* operon (*P. parmentieri* and *P. wasabiae*), or upstream of *cas1* gene and after the CRISPR loci (*P. c*. subsp. *carotovorum* BC T5, *P. c*. subsp. *brasiliense* all three strains, *P. c*. subsp. *odoriferum* T4, *P. betavasculorum* NCPPB2795, *P. polaris* NIBIO1392, and *P. peruviense* A350-S18-N16). These genes were identified as coding sequences of the Lrp/AsnC transcriptional regulator protein, YitT integral membrane protein, an M18 peptidase, the type VI secretion component Hcp (hemolysin-coregulated protein), and other hypothetical proteins. Interestingly, we found a region of 49 CDS that interrupted the *cas* operon between the genes *csy3* and *csy2* in *P. c.* subsp. *brasiliense* SX309. This region harbored proteins that were related to phage structures, suggesting that the subtype I-F CRISPR-Cas system, in this strain, has a horizontally transferred origin. Unlike the subtype I-F CRISPR-Cas system, the subtype I-E possessed different types of toxin-antitoxin systems localized either downstream of the *cas2* gene or between the *cas3* and c*se1* operons (Figure 10B). Specifically, we found *P. c.* susbp. *carotovorum* T5 and T2 contained RelE/ParE toxin protein that was located between the CRISPR-1 and CRISPR-2, whereas all of the strains within *P. parmentieri* presented the same protein with additional toxin protein HigB. In *P. wasabiae*, a toxin protein, called pemK, was observed downstream of *cas3. P. polaris* NIBIO1392 harbored a hypothetical protein, HicA toxin, and HicB antitoxin next to Cas3 operon. Strains SCC3193 and RNS 08-42-1A of *P. parmentieri* contained the same antitoxin HicA adjacent to the *cas3* operon. Furthermore, other species, such as *P. c.* subsp. *carotovorum*, *P. c.* subsp. *brasiliense*, *P. parmentieri*, and *P. wasabiae*, contained locus predicted to encode a transcriptional regulator (blue arrows in Figure 10B). The type III-A CRISPR-Cas system was exclusively found in *P. arodiearum* PC1 (Figure 10C). This system harbored two hypothetical proteins, one between the genes *cms3* and *cms5* and the other between *cas6* and CRISPR locus.

We performed a BLASTn analysis since the entire CRISPR-Cas system was found within a genomic island (GI-22). The corresponding results showed a 90% nucleotide identity with *Serratia* sp. ATCC39066 with over 99% of query cover. A similar homology percentage was observed with *D. zeae* Ech586, but with lower query cover (97%). These results suggest that *P. aroidearum* acquired the entire type III-A CRISPR-Cas system from *Serratia* sp. through a horizontally transferred mechanism. A set of features was observed in predicted CRISPRs loci. Table S40 provides specific information regarding the main features of each CRISPR, such as position, length of direct repeats, number of spacers, and orientation. Generally, the length of direct repeats within all CRISPRs was 28- to 29-bp except directed repeats of CRISPR located within type III-A CRISPR-Cas system of *P. aroidearum*, whose size was 37 bp. In terms of the total size of the CRISPRs, we observed a plethora of variabilitfrom shortest CRIPSRs of just 208 bp, CRISPR-1 of *P. wasabiae* strains CFBP 3304 and NCPPB 3701, and the largest CRISPRs with a size of 2250 bp—predicted for CRISPR-1 in *P. betavasculorum* NCPPB 2793. A size of 2488 bp found in CRISPR-2 of *C.* P. maceratum F135.

However, the highest number of 41 spacers were detected in CRISPR-2 of *C.* P. maceratum (F135), and *P. c.* subsp. *carotovorum* (PCC21). *P. polaris* NIBIO1392, and *P. betavasculorum* NCPPB 2793 reached the highest number of six CRISPRs loci, while *P. aroidearum* and *P. peruviense* A97-S13-F16 only showed one CRISPR. Finally, we observed the presence of orphan CRISPRs that were distant from the *cas* operon regions—*P. betavasculorum* NCPPB 2793 contained four orphan CRISPRs loci. Other species, such as *P. c.* subsp. *odoriferum* strains (BC S7 and S6), and *C.* P. maceratum (F135) harbored two orphan CRSIPRs, whereas *P. c.* subsp. *carotovorum* BC T2 and *P. polaris* (NCPPB 3395 and SS28) possessed one orphan CRISPR. 

**Figure 11:**
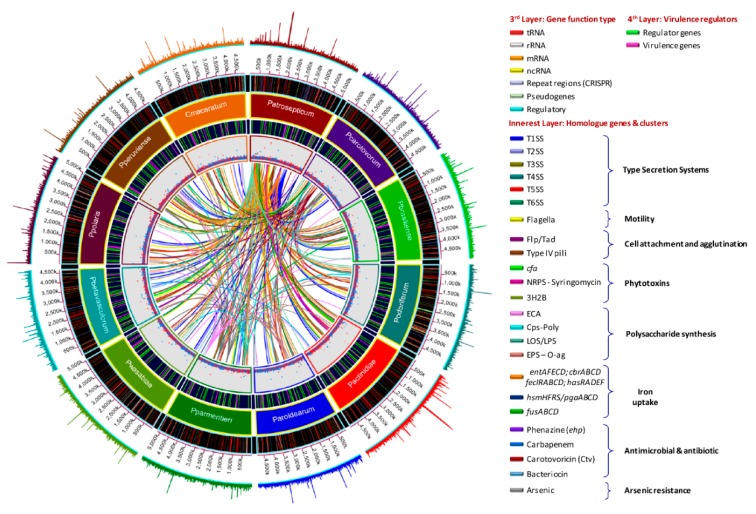
Circos plot description of main genetic features, relevant virulence, and antibiotic biosynthesis clusters among all *Pectobacterium* species. Seven circular layers have been rendered to show unique features based upon the comparative genomic analyses. From the outermost layer to the innermost layer: the area of each genome; sizes of each genome (axis in kb); gene function types across the species, name of each species indicated in 3rd layer—acronyms used in the plot starting with *Pectobacterium atrospecticum* (Patrosepticum), *P. carotovorum* subsp. *carotovorum* (Pcarotovorum), *P. c.* subsp. *brasiliense* (Pbrasiliense), *P. c.* subsp. *odoriferum* (Podoriferum), *P. c.* subsp. *actinidiae* (Pactinidiae), *P. aroidearum* (Paroidearum), *P. parmentieri* (Pparmentieri), *P. wasabiae* (Pwasabiae), *P. betavasculorum* (Pbetavasculorum), *P. polaris* (Ppolaris), *P. peruviense* (Pperuviense), and *Candidatus* Pectobacterium maceratum (Cmaceratum); virulence genes (pink lane) and key gene regulators (green lane), as indicated in the legend (5th layer); scattered dots of all genes, where the red and blue dots depict negative and positive gene orientation, respectively; and the inner layer showing the connection among homologues genes of all gene clusters covered in this study. The colors of the connector lanes of each cluster are labeled on the right side of the figure. Expanded legend entry acronyms are provided: T1SS (Type I secretion system), T2SS (Type II secretion system), T3SS (Type III secretion system), T4SS (Type IV secretion system), T5SS (Type V secretion system), T6SS (Type VI secretion system), Flp/Tad (Fimbrial low-molecular-weight protein/Tight adherence protein), *cfa* (coronofacic acid), NRPS (Non-Ribosomal Peptide-Synthetase) like syringomycin, 3H2B (3-hydroxy-2-butanone pathway), ECA (Enterobacteria Common Antigen), Cps-Poly (capsular polysaccharide), LOS/LPS (lipopolysaccharide/lipooligosaccharide), EPS—O-ag (Exopolysaccharide O-antigen), *entAFECD* (enterobactin), *cbrABCD* (achromobactin), *fecIRABCD* (ferric citrate uptake), *hasRADEF* (heme acquisition), *hsmHFRS/pgaABCD* (hemin storage), and *fusABCD* (ferredoxin uptake).

In summary, Figure 11 illustrates a circos plot that describes primary genetic features as well as all homolog genes that are associated with pathogenicity determinants and the synthesis of antimicrobial compounds mentioned in this study.

## 4. Discussion

In this study, we performed genome-wide comparative analyses of 12 *Pectobacterium* species. The genetic relationships among the *Pectobacterium* species were evaluated using different approaches: ANI, dDDH, phylogenetics, codon and amino acid usage, and pan- and core-genome. In addition, different virulence determinants were analyzed. A highly conserved novel cluster, composed of five genes that were involved in phosphotransferase system (PTS) of cellobiose, was identified exclusively in *P. parmentieri*. 

Among the 12 *Pectobacterium* species, *P. betavasculorum* exhibited the shortest genome size and the lowest amount of coding sequences (CDS) (Table 1). *P. c.* subsp. *odoriferum* possessed the highest number of pseudogenes, which were observed within important clusters, including type secretion systems I, III, V, and VI, some PCWDE and polysaccharides clusters—indicating a genomic degeneration in the coding ability of this species. Similar features were observed in the comparative genomics of five *Clavibacter michiganensis* subspecies, where *C. michiganensis* subsp. *sepedonicus* contained the fewest number of coding sequences, which was suggested to be the result of disposable genes to adapt into a new niche [100]. *P. c.* susbp. *odoriferum* could possibly invade the plant tissues and cause soft rot in potato as well as chicory [25] with fewer number of genes. Contrary to *P. c.* subsp. *odoriferum*, *P. parmentieri* presented the largest genome and highest number of coding genes. These features may be the consequence of horizontal gene transfer. Nykyri et al. [26] reported a total of 56 genomic islands in this species. By analyzing the genomic islands throughout the genus *Pectobacterium* (Tables S2–S13), *P. parmentieri* presented the highest number of GIs among the species.

DNA structural properties, namely intrinsic curvature, stacking energy, and position reference, which were visualized in genome atlases (Figure S1), showed significant differences among the species. Regions with high gene expression are the genomic sequences with very low position preference, and not easy to condense [50]. *P. parmentieri* showed the lowest high position preference areas; thus, it can be concluded that *P. parmentieri* would have more highly expressed regions. *P. c.* subsp. *carotovorum*, followed by *P. aroidearum* and *P. c.* subsp. *odoriferum* led the content of the highest stacking energy and intrinsic curvature areas. Genomic regions with high levels of intrinsic curvature and stacking energy are thought to display high transcriptional levels mediated by nucleosome [50]. In *Pseudomonas putida*, high intrinsic curvature and stacking energy were linked with high recombination levels that lacked synteny [101]. Hence, it could be possible that the genome atlases of aforementioned *Pectobacterium* species indicate relevant hotspots that were involved in recombination and transcriptional events. DNA curvature participates in crucial biological functions, such as replication, transcription, recombination, and nucleosome positioning [102]. The intrinsic DNA curvature might indicate fingerprints that demonstrate how different *Pectobacterium* species have evolved and adapted in a new environment. A reduction of intrinsic DNA curvature has been proven to increase local mutation rates [103]; thus, these regions may be associated with mutation levels and speciation in *Pectobacterium. P. atrosepticum* harbored the highest number of either direct or indirect repeats—an indication of carrying more unstable regions within the genome [104]. 

ANI, dDDH, and proteome comparison (Figure 1 and Figure 3) showed that *P. parmentieri* and *P. wasabiae* shared lowest nucleotide identity with other species of *Pectobacterium*–similar results were obtained with codon and amino acid usage profiles (Figure 4) as well as with pan- and core-genome dendrogram (Figure 5B). This information showed that both bacteria might have evolved differently, and that could be the reason for not harboring T3SS. Phylogenetic analysis distinguished all 12 species clearly (Figure 2); two strains—PCC21 and ATCC 39048, named as *P. c.* subsp. *carotovorum*, but grouped within the cluster of *P. c.* subsp *brasiliense*, indicating that both strains were incorrectly named. ANI and dDDH values showed the highest homology of *C.* P. maceratum with *P. c.* subsp. *odoriferum*, which agreed with previous findings [20]. Nevertheless, the rest of *in-silico* analyses, including the phylogenetic tree, BLAST matrix, codon and amino acid usages, and pan and core genome dendrogram revealed that *C.* P. maceratum clustered close to *P. polaris*. 

The pan-core genome analysis showed a pan and core genome values of 9296 and 2414 genes, respectively (Figure 5A). Glasner et al. [8] compared *P. atrosepticum*, *P. c.* subsp. *carotovorum* and *P. c.* subsp. *brasiliense* and reported pan and core genome sizes of 6065 and 3264, respectively. In a recent study conducted in 2018 [24], compared *P. c.* subsp. *carotovorum*, *P. c.* subsp. *brasiliense* and *P. c.* subsp. *odoriferum* and revealed a pan and core genome sizes of 3107 and 3903, respectively—indicating great similarity between these subspecies. The significant increase and decrease in pan and core genomes, respectively, indicate high heterogeneity. 

Three types of T1SS have been described in *Pectobacterium*: the *prtDEF* operon involved in the secretion of hydrolases, a T1SS that modulates iron acquisition through the secretion of hemophore HasA, and a T1SS that secretes a proteinaceous multi-repeat adhesin protein (MRP) [65]. Only T1SS involved in iron uptake was not present in *P. parmentieri*, *P. wasabiae*, and *P. betavasculorum*, which suggests the inefficiency of these *Pectobacterium* species over the other species in iron-scavenging process. On the other hand, the length of the gene encoding the adhesin protein (MRP) significantly differed among the species (Figure S5). This MRP has been confirmed to mediate bacterial attachment into the plant tissue [71]. We hypothesized that the differences within this gene could be associated with host specificity since *P. atrosepticum*, *P. betavasculorum*, *P. c.* subsp. *actinidiae*, and *P. wasabiae* are restricted to potato, sugar beet, kiwi, and Japanish horseradish, respectively. The other two T1SS and T2SS clusters appeared to be conserved in all species (Tables S14 and S15). Only the *outN* gene was not present in *P. parmentieri* and *P. wasabiae* (Table S15 and Figure S6). However, this gene has not been considered to be an essential element of T2SS [73]. The primary function of T2SS is the translocation of the PCWDE to the extracellular medium [27,72]. Almost all PCWDE contributed to the core genome. However, the HrpK protein with a pectate lyase domain, a pectate lyase located nearby the T2SS cluster (Figure S6), and the polygalacturonase PehK were absent in both *P. parmentieri* and *P. wasabiae*. These results are congruent with the outcomes by Nykyri et al. [26]. Contrary to the PCWDE, the proteases are secreted by T1SS, known as *prtDEF* operon [27,65]. Except some proteases commonly found in all—mainly metalloproteases PrtW and Prt1—others were observed to be unique in *P. aoridearum*, *P. parmentieri*, and *P. wasabiae* (Table S16). These findings suggest that some proteases, unlike the PCWDE, tend to vary and are specific to certain species. 

In the case of T3SS, only *P. parmentieri* and *P. wasabiae* lacked this cluster (Table S17). Kim et al. [105] demonstrated that *P. parmentieri* WPP163 was able to cause disease in plant stems and tubers; thus, confirming that T3SS is not essential in *Pectobacterium* species for disease development. The other 10 species harbor T3SS exhibited variable regions that were composed of unknown proteins and components of T6SS between the loci *hrpN* and *hrpW* (Figure 9). The influence of this variable region on the function of the T3SS has yet to be proved. Additionally, a *hrpK* gene, adjacent to the T3SS cluster and predicted to encode a T3SS dependent effector protein, was found in *P. c*. subsp. *carotovorum*, *P. c*. subsp. *brasiliense*, *P. c.* subsp. *actinidiae*, *P. aroidearum*, *P. polaris*, *P. peruviense*, and *C.* P. maceratum (Table S17). Glasner et al. [8] also found this gene in *P. c.* susbp. *carotovorum* WPP14 and *P. c.* subsp. *brasiliense* 1692. However, the role of this gene during plant infection is still unknown; indeed, the HrpK-like effector was found not to be fundamental in the promotion of cell death in tobacco or translocation of the effector DspE [8,106]. 

Some *Pectobacterium* species harbor the conjugation system virB-T4SS of *Agrobacterium tumefaciens*—this was the case of *P. atrosepticum* [21], *P. c.* subsp. *brasiliense* 1692 [8], *P. parmentieri* SCC3193, and *P. wasabiae* CFBP 3304 [26]. *P. c.* subsp. *carotovorum* WPP14 was described to possess an incomplete *virB* operon [8], while *P. parmentieri* WPP163 and *P. aroidearum* lacked this cluster [26]. In our analyses, we identified a virB-T4SS in *P. c.* subsp. *actinidiae* KKH3 and *P. betavasculorum* NCPPB2795, and surprisingly, *P. c.* subsp. *brasiliense* BC1 harbor two virB-T4SS clusters (Figure S7 and Table S18). However, two other strains of *P. c.* subsp. *brasiliense* (SX309 and BZA12) did not carry two copies of this cluster. The virB-T4SS has been reportedly harbored within a genomic island in *P. atrosepticum* [21]. This indicates that the virB-T4SS is distributed sporadically throughout the species and even strains within *Pectobacterium* genus. 

*Pectobacterium* harbors a T5SS known as the two-partner secretion system (type Vb or Tps) [65]. In *P. atrosepticum*, the T5SS, located next to the T3SS, consisted a *hecB* gene that encodes for a small transporter TpsB, and two *hecA* genes that encode an exoprotein, called hemagglutinin [21]. The same pattern was observed for the remaining species harbored the T3SS, except for *P. c.* subsp. *actinidiae*, where the cluster was found elsewhere in the chromosome. Likewise, the sizes and number of copies of *hecA* gene varied widely among the species. In *D. chrysanthemy* EC16, the hemagglutinin that was encoded by *hecA* was observed to provide attachment, aggregation, and promote cell death in the leaves of *Nicotiana clevelandii* [107]. The fact that the HecA protein is involved in cell host adhesion, like the MRP protein, reinforces our hypothesis that the genetic variability of both proteins lies in the *Pectobacterium* resulted in host specificity. Besides the HecA, TpsB reportedly secretes other cytotoxins and growing inhibitors [76]. In *P. parmentieri* strains WPP163 and SCC3193, for instance, a contact-dependent growth inhibition (CDI) was described [26,108]. Poole et al. [108] reported a novel Tps system, called rearrangement hotspot (Rhs), whose function is involved in toxicity and intercellular competition against other bacteria. Additionally, we identified a novel Tps cluster that resembles the *hecAB* operon that presents a toxin between the *hecB* and *hecA* loci instead of harboring two copies of the *hecA* gene (Table S14 and Figure S8). This cluster was not found in *P. wasabiae* and *P. peruviense*. The different types of T5SS reported in *Pectobacterium*, and the new cluster that was identified in this study, highlighted the possibility that uncharacterized T5SS might play important role in infection and disease development.

Genomic analysis revealed a T6SS in all *Pectobacterium* species (Table S20 and Figure S9). However, a part of this cluster was not present in *P. betavascolorum*, while the T6SS of *P. c.* subsp. *odoriferum* harbor an extra set of genes that encoded for unknown proteins. Nykyri et al. [26] reported that *P. parmentieri* strains SCC3193, WPP163, and *P. wasabiae* CFBP3304 contain two T6SS whose function seems to be redundant. A handful of functions have been associated with T6SS, including interaction with host eukaryotic cells, pathogenicity, antibacterial activity, symbiosis, metal ion acquisition, and biofilm formation [77,80,109]. In *P. atrosepticum*, this secretion system allegedly alters the plant defense mechanisms [110]. However, Mattinen et al. [81] stated that the T6SS function is to secret Hcp proteins, rather than virulence. Haemolysin-coregulated protein (Hcp) and valine-glycine repeat protein (VgrG), the major effector proteins of the T6SS [78,79], have been described to be present in several copies and scattered in the chromosomes of *P. atrosepticum*, *P. parmentieri*, and *P. wasabiae* [26,81]. The same pattern was observed for the other species in the current study. Nykyri et al. [26] indicated that the number of *hcp* and *vgrG* genes differs among species and at the strain level. The distinct genetic organization of this cluster indicates the need for further studies to clarify the functions of T6SS and decipher how the T6SS-effectors act during disease development. 

*P. atrosepticum* and *P. parmentieri* have a type IV pili or fimbriae cluster, predicted to confer adhesion into the cell hosts [111]. We identify a similar *pil* operon in *P. peruviense* within a genomic island (Figure S10). Furthermore, we found a gene termed *pilW* and another operon consisted of three genes—*ppdD*, *hofB*, and *hofC*—that are homologues in all species (Table S21). In *Nisseria meningitis*, *pilW* stabilized the pilus fiber [112] and, in *Pseudomonas aeruginosa*, involved in the insertion and multimerization of PilQ [113]. The *pilABC* operon, homologous to the *ppdD*, *hofB*, and *hofC*, has been associated with bacterial adherence in *P. aeruginosa* [114,115]. In *Pectobacterium*, the functionality of *pilW* and *pilABC* is still to be elucidated. The Flp/Tad pilus [116], another class of fimbriae, was harbored by the 12 species (Table S22 and Figure S11). Nykyri et al. [116] proved the association of *flp*/*tad* operon with maceration of potato tubers, highlighted the importance of this cluster during disease development, which would explain why this was highly conserved in *Pectobacterium*.

The biosynthesis of phytotoxins, such as coronofacic acid (*cfa*) and syringomycin, are described as important pathogenicity determinants in *Pectobacterium* [27]. The *cfa* cluster was first reported in *P. atrosepticum* within a genome island, and its capacity to boost blackleg disease development has already been proven [21]. Panda et al. [117] showed a similar *cfa* operon in the genome islands of *P. atrosepticum* IMCP 1526, *P. c.* subsp. *brasiliense* IMCP19477, and *P. peruviense* IFB5232 (formerly *P. c.* subsp. *carotovorum* UGC32). We identified the same *cfa* cluster in *P. peruviense* IFB5229 (Figure 7) as well as in the kiwi and sugar beet pathogens *P. c.* subsp. *actinidiae* and *P. betavasculorum* (Figure S12), and, like the other species, this phytotoxin operon was contained in GIs, which indicates that this cluster was acquired by other *Pectobacterium* species and strains via horizontal genetic transfer. Consistent with this inference, Panda et al. [117] suggested that *cfa* could be acquired prior speciation of *P. atrosepticum*. The NRPS, like syringomycin, was also predicted into a GI in *P. atrosepticum* [21] and *P. c* subsp. *brasiliense* ICMP 19477. In our analysis, only *P. c.* subsp. *actinidiae* had a similar NRPS-syringomycin cluster (Table S14), which was harbored into a GI. Although the syringomycin toxin was reported to induce electrolyte leakage, leading to necrosis symptoms in *P. syringae* [118], the strict virulence role of this toxin has not been clarified in *Pectobacterium*. Our finding of syringomycin cluster in *P. c.* susbp. *actinidiae* brings new research to decipher whether this phytotoxin contributes to the development of canker disease in kiwi fruits.

The acquisition of arsenic clusters (*ars*) confers the ability to resist arsenic concentration in the environment [83]. These *ars* genes can be part of the chromosome or plasmid or have acquired through horizontal gene transfer [83]. *P. parmentieri* SCC3193 possess an arsenic resistance cluster of 13,686 bp harbored within a GIs [26]. *P. atrosepticum* presents four genes of this cluster, also contained into the HAI7 [21]. We observed that *P. c.* subsp. *brasiliense*, *P. peruviense*, and *C.* P. maceratum have orthologues to the *arsC*, *arsB*, and *arsR* genes of *P. atrosepticum*. However, the complete *ars* operon of *P. parmentieri* SCC3193 was not identified in any of the *Pectobacterium* species (Figure S13) or other *P. parmentieri* strains. Nykyri et al. [26] reported that even *P. parmentieri* WPP163 and its close relative *P. wasabiae* lacked this arsenic cluster. Since *P. parmentieri* SCC3193 was isolated from southern Finland, whose cultivated soils exhibits the highest arsenic concentrations [26], it is logical to assume that this phytobacteria have evolved and acquired a complete *ars* cluster to adapt into this niche. The *arsC* gene of *P. parmentieri* was described to present two additional copies that were located elsewhere in the genome [26]. One locus is shared across all species (Table S25). Likewise, we identified an arsenic resistance protein (*arsH*) that displayed a high identity in all species. The presence of these two genes suggests that they are vital for living and bacterial growth inside ecological niches surrounded by arsenic components.

Members of the *Pectobacterium* genus have been reported to produce cell surface polysaccharides [27]. *P. atrosepticum*, for instance, harbors a capsular polysaccharide cluster (*cps*) [21] that—according to our data—is highly conserved, but only present in few species (Table S26 and Figure S14). Except ECA cluster—shared by all species (Table S27 and Figure S15), the EPS (extracellular polysaccharides), and O-antigen cluster of *P. atrosepticum* [21] and lipo-oligo/polysaccharide cluster (LOS/LPS) of *P. parmentieri* SCC3193 [26] were partially conserved and displayed distinct genetic rearrangements (Tables S28 and S29 and Figures S16 and S17). Evans et al. [119] observed that mutants in the LPS biosynthesis cluster of *P. atrosepticum* SCRI1043 led to a reduction in potato soft rot. We assumed that divergences that were observed in EPS and LPS of *Pectobacterium* species could be the result of specialized adaptive mechanisms that each species has developed to survive. 

Iron sequestration constitutes a crucial step for bacterial pathogens during the infection process [84]. Bell et al. [21] reported five different iron uptake systems in *P. atrosepticum* SCRI1043, including achromobactin, enterobactin, ferric citrate, hemophore, and hemin storage. The achromobactin (*cbrABCD*) was present in all species (Table S30), which suggests that this operon is crucial for iron assimilation, especially in niches with poor iron content. Contrary to *cbrABCD*, the enterobactin (*entAFBECD*), heme operon (*hasRADEF*), and ferric citrate uptake system (*fecIRABCDE*) were either partially present or absent in *P. parmentieri* and *P. wasabiae*. The hemin storage cluster (*hmsHFRS*) was identified in few species: *P. atrosepticum*, *P. aroidearum*, *P. parmentieri*, *P. wasabiae*, and *P. peruviense*—this operon was involved in biofilm matrix formation [120]. The aerobactin cluster (*iuc*), found first in *P. c.* subsp. *carotovorum* W3C105 [85] and later in *P. parmentieri* SCC3193 [26], also found in other *P. parmentieri* strains (Table S31). This finding suggests that aerobactin is a distinctive characteristic of *P. parmentieri*, and might subtitute the absence of ferric citrate and heme acquisition clusters. A recently plant-ferredoxin uptake system (*fusABCD*), characterized in *P. c.* subsp. *carotovorum* PCC21 [121,122], was harbored in almost all species except for *P. betavasculorum*, which also lacked *fecIRABCDE*, *hasRADEF*, and *hmsHFRS* clusters. 

Medium alkalization was described to be crucial for the accurate activity of pectate lyases during potato tubers infection in *P. c.* subsp. *carotovorum* WPP14 [92]. This function was described to be governed by acetoin or the 3-hydroxy-2-butanone pathway (3H2B) that was encoded by the *bud* operon [92]. In our analysis, this operon was highly conserved in all 12 *Pectobacterium* species (Table S33 and Figure S18), which suggests that the 3H2B pathway is a vital component at the early stages of infection not only in potato, but also in other *Pectobacterium* hosts, such as kiwi, sugar beet, cucumber, chicory, and Chinese cabbage. 

Motility is a fundamental virulence factor in motile bacteria [27]. The flagella cluster was highly conserved in the entire *Pectobacteirum* genus. In accordance with Nykyri et al. [26], we also observed that, in all species, the flagella genes were located within predicted GIs (Tables S2–S13). Therefore, it seems that the common ancestor of all *Pectobacterium* species acquired the flagellar apparatus via horizontal DNA transfer in order to move inside the plant cells, rapidly colonize the plant host tissues, or scape from harmful environmental conditions. Previous reports demonstrated that flagellar genes were involved in chemotaxis, and motility is fundamental for pathogenicity in *D. dadantii*, whereas, in *Pectobacterium*, FliC elicited the plant cell defenses [65]. The non-motile mutants of *P. parmentieri* SCC3193 displayed a diminished virulence in tobacco [123]. Furthermore, it observed that the flagella were involved in colicin delivery, a toxin that kills closely related species, suggesting that the flagella operon in *Pectobacterium* can also be used as a weapon against other bacteria [65]. 

The single gene encoded virulence factors carried by all *Pectobacterium* species (Table S32). These loci include: the necrosis inducing virulence protein (Nip)—observed in *P. parmentieri* SCC3193, *P. atrosepticum* SCRI1043, and *P. c*. subsp. *carotovorum* ATTn10 essential for virulence in tuber [87,124]; the *svx* gene, whose inactivation in *P. atrosepticum* SCRI1043 decreased virulence in potato [86]; the citrate transporter encoded by *cit1* gene, which is involved in citrate uptake and tuber maceration [88]; the *rplY* gene, proved to be crucial in pathogenesis in *P. carotovorum* PccS1 [91]. We speculate that these genes constitute fundamental elements for the virulence lifestyle of entire genus. In contrast to these loci, the *sax* operon [90], the phytase gene *appA* [89], and the *evf* [9] were absent in few *Pectobacterium* species (Table S32). The *Erwinia* virulence factor (*evf*), for instance, was proven to allow the interactions of some *P. carotovorum* strains and enhanced bacterial persistence into the gut of *D. melanogaster*, thus contributing the spreading of these bacteria [9]. In our analysis, only *C.* P. maceratum harbored this gene. Similarly, the *evf* gene was also found in *P. carotovorum* SCC1 [34], as proposed by Shirshikov et al. [20], as the type strain of *C.* P. maceratum. 

Signal molecules regulate most of the pathogenicity factors, which act as a complex network system [27,65]. Mutagenesis conducted in some of these regulators significantly affected the virulence and growth rate of *Pectobacterium* sp. [27,65,110]. In our data, nearly all of the virulence regulators were harbored by all *Pectobacterium* species (Table S34), indicating their crucial role in bacterial survival, growth, and disease development.

The secretion of antimicrobial compounds boosts ecological fitness and allows for bacteria to outcompete endophytes or secondary invaders [8]. In *Pectobacterium*, four components have been described: carotovoricin (Ctv) [31,32], carbapenem [28], phenazine [21], and bacteriocins (carocins) [29,30,68]. Only the carotovoricin (*ctv*), which was composed of 21 genes encoding phage structures [67], was found in all *Pectobacterium* species (Table S35) and adjacent to the T1SS (the *prtDEF* operon) (Figure S19). The location of the *ctv* operon close to T1SS shows that this bactericide may have evolved together with *prtDEF* operon. Significant genomic conservation of T1SS+Ctv block was found in 96.7% of *Pectobacterium* genomes, which suggests that T1SS might export Ctv elements through the periplasm [39]. Contrary to the carotovoricin, the carbapenem cluster was observed in few species (Table S36). *P. c.* subsp. *carotovorum* ATTC 39048 was the first species reported as carbapenem producer bacterium [95]. Later, Glasner et al. [8] reported in *P. c.* subsp. *brasiliense* 1692 the same *car* cluster, which inhibited the growth of *P. c.* subsp. *carotovorum* WPP14 and *P. atrosepticum* SCRI1043 [92]. We identified the *car* operon in all *P. c.* subsp. *actinidiae* and *P. betavasculorum* strains (Table S36 and Figure S20). Surprisingly, *P. c.* subsp. *brasiliense* BC1, *P. parmentieri*, and *P. wasabiae* only presented *carF*, *carG*, and *carH* genes (Figure S20). As the *carF* and *carG* genes confer protection against the carbapenem [96], it seems that these three species may be immune against the antibiosis effect of carbapenem. The phenazine (*ehp*), on the other hand, has been found within genome islands of *P. c.* subsp. *brasiliense* ICMP19477 and *P. atrosepticum* strains SCRI1043 and ICMP1526 [21]. In the present study, a similar synteny of phenazine cluster was observed in all *P. c.* subsp. *actinidiae* and *P. betavasculorum* strains (Table S37 and Figure S21). 

Several bacteriocins have already been identified within *Pectobacterium* genus, including Carocin S1 [29], Carocin D produced by *P. c.* subsp. *carotovorum* PCC21 [69], Carocin S2 of *P. c.* subsp. *carotovorum* F-rif-18 [68], two colicin M-like bacteriocins with a ferredoxin domain termed Pectocin M1 and M2 characterized in *P. aroidearum* PC1 and *P. c.* subsp. *brasiliense* 1692, respectively [98]. We found that *P. c.* susbp. *brasiliense* BC1, *P. c.* subsp. *odoriferum* BC S7, and *P. betavasculorum* harbor homologous Carocin D. Further, a type of unreported colicin/pyocin-like bacteriocins were observed in *P. carotovorum* subspecies (*carotovorum*, *brasiliense*, and *odoriferum*) and in *P. wasabiae* (Table S38). *P. c*. subsp. *actinidiae* and *P. betavasculorum* contained the phenazine and carbapenem clusters, which have a broad antibiosis spectrum [125,126], might explain why other *Pectobacterium* species have not been isolated yet from kiwi and sugar beet plants. 

Multiple genome alignment led us to discern a ~4.54 kb novel cellobiose-specific phosphotransferase system (PTS) specific for all *P. parmentieri* strains (Figure S22). In *Klebsiella pneumoniae*, the *celB* gene, encoding component EIIC within the cellobiose-specific phosphotransferase system, was reported to be involved in biofilm formation as well as in cellobiose transport; showed a dual role of this cellobiose-specific PTS and its contribution in virulence [127]. The fructose-specific PTS permease IIA was proven to facilitate the biofilm formation and mediate the stress response in *Listeria monocytogenes* [128]. When considering this previous data, it can be speculated that the uncharacterized cellobiose-PTS found in our study might have involved in virulence functions and, therefore, could be a pathogenic determinant in *P. parmentieri*.

The CRISPR-Cas is defined as the innate and adaptive immune system of bacteria [99]. We articulated that the presence of two CRISPR-Cas systems in some *Pectobacterium* species (Figure 10) might confer a better immune system against invasive elements. *P. aroidearum* PC1 was the only species carried type III-A CRISPR-Cas (Figure 10C); this CRISPR type was found with less frequency among the bacterial species [129]. Interestingly, the entire CRISPR-Cas in PC1 was located within a genomic island (GI-22; Table S7) and exhibited 90% homology with *Serratia* sp. ATCC 39066; thus, indicating a DNA transfer event of this system. Consistent with this outcome, the CRISPR-Cas systems have been suggested to be mobile genetic elements acquired via horizontal gene transfer [130], where plasmids, and even phages, seem to have facilitated the spreading of this immunity system across the bacterial taxa [131,132,133]. We observed a peculiar region of 49 CDS between the *csy*3 and *csy*2 genes in *P. c.* subsp. *brasiliense* SX309 (Figure 10A). This region encoded proteins related to structures of phages, suggesting that the subtype I-F CRISPR-Cas might have a phage origin in SX309. Moreover, the lack of CRISPR-Cas in *P. polaris* NIBIO1006 and *P. peruviense* IFB5229 supports the theory that CRISPR-Cas can be acquired by certain species, but not by all. Different toxin-antitoxin systems were distinguished between the CRISPR loci and the *cas* genes (Figure 10A,B). Kooni and Makarova [134] observed that prokaryotic toxins are tightly associated with CRISPR-Cas and they trigger cell death or dormancy in the case of an immune system failure. Numerous toxin and antitoxin genes were solely found surrounding the type I CRISPR-Cas system [135]—these findings are in concordant to our outcomes, and reinforce the hypothesis that CRISPR-Cas and suicide response mediated by toxin-antitoxin systems. Another aspect of being highlighted was the presence of an Hcp protein, a T6SS component (Figure 10A), within the subtype I-F CRISPR-Cas. Congruently with our information, a ~28 kb GI containing a CRISPR-Cas system and proteins of the T6SS (VgrG, Hcp, and PAAR) were reported in *Vibrio cholerae* S12; showed the importance of GIs as propagators of CRISPR-Cas and T6SS [136]. Different CRISPR arrays, distinct sizes, amounts, and locations were observed within genus *Pectobacterium* (Table S40). We identified strains with just three spacers, while others with 41. Usually, CRISPR loci are next to the *cas* genes [137]. However, CRISPR arrays have also been reported to be located distantly from the *cas* operon in *Listeria monocytogenes*, *Aggregatibacter actinomycetemcomitans*, and *Enterococcus faecalis* [138]. The same pattern was observed with some *Pectobacterium* species (Figure 11). In our analyses, 13 orphans CRISPRs were found throughout the 34 analyzed *Pectobacterium* genomes (Table S40). Zhang et al. [138] reported that some CRISPRs seem to exert their function far away from the *cas* locus, although they might not be functional. Molecular assays are necessary for proving whether the orphan CRISPRs found in some *Pectobacterium* species are functional or just remnants of previous CRISPR-Cas systems. 

## 5. Conclusions

To conclude, the general genomic features showed that *P. c.* subsp. *odoriferum* possessed the highest number of pseudogenes, indicating a coding ability degeneration, while *P. parmentieri* harbored the largest number of CDS. Zones of high expression levels that seem to be controlled by the nucleosome were found in the genome atlases of *P. c.* subsp. *carotovorum*, *P. aroidearum*, and *P. c.* subsp. *odoriferum*, whereas *P. atrosepticum* appeared to encompass more unstable regions. Our comparative analysis unveiled that the T2SS, T6SS, T1SS, some PCWDE and proteases, *bud* operon, the ECA cluster, achromobactin, the flagella genes, single virulence locus, the *pilW* and *pilABC* genes, Flp/Tad, carotovoricin, and the majority of virulence regulators appear to be crucial components for the survival and virulence lifestyle of the entire *Pectobacterium* genus. In contrast, T3SS, T4SS, T5SS, phytotoxins, type IV pilus, capsular polysaccharide, lipopolysaccharides, exopolysaccharides, iron uptake systems, phenazine, carbapenem, and colicin-like bacteriocins were exclusively observed in some species; highlighting that species harboring these elements might have an advantage over the others. Our study found the novel cellobiose phosphotransferase system (PTS) exclusively present in *P. parmentieri*, and an unreported T5SS conserved in almost all *Pectobacterium* species. Three CRISPR-Cas systems: I-E, I-F, and III-A, along with toxin-antitoxin systems and Hcp proteins, were found. Several pathogenicity determinants, antimicrobial clusters, and the type III-A CRISPR-Cas system were located within genomic islands, indicating the influence of horizontal gene transfer in this genus. We believe that our findings reported in this manuscript constitute significant insights regarding how different *Pectobacterium* species are equipped, and have evolved to outcompete with their relatives, and adapted host specificity. Further, the findings of novel clusters and general description of CRISPR-Cas systems provide ample opportunities for future investigations on *Pectobacterium*. 

## Figures and Tables

**Table 1:** General genome characteristics described for *Pectobacterium* species.

Genome Features	Pectobacterium *species*											
	Pa	*P. carotovorum* subspecies				Par	Pp	Pw	Pb ^†^	Ppo	Ppe ^†^	Cpm ^†^
		Pcc	Pcb	Pco	Pca ^†^							
Length (bp)	5,064,019	4,842,771	4,920,350	4,933,575	4,922,167	4,862,913	5,164,411	5,043,228	4,685,210	5,008,416	4,785,880	4,993,011
% GC	51	52.2	51.84	51.75	51.54	51.9	50.4	50.55	51.2	51.99	51.1	51.82
CDS (coding)	4381	4115	4267	3855	4151	4232	4449	4416	4070	4301	4158	4338
Proteins with EC number appointed *	1107	1078	1082	1103	1111	1094	1113	1089	1078	1113	1143	1123
Proteins involved in metabolic pathways *	829	799	806	814	828	813	835	805	802	813	835	813
rRNAs (5S, 16S, 23S)	8, 7, 7	8, 7, 7	8, 7, 7	8, 7, 7	7, 7, 7	8, 7, 7	8, 7, 7	8, 7, 7	7, 8, 1	8, 7, 7	2, 1, 2	8, 8, 3
tRNAs	77	76	77	77	76	78	77	77	71	77	66	70
ncRNAs	10	6	9	1	6	8	7	8	9	5	7	6
Pseudogenes	125	121	97	385	103	53	286	184	200	116	106	107
Virulence factor *	48	46	47	50	50	47	53	48	43	46	52	47
Antibiotic resistance *	60	54	56	53	53	53	54	57	45	56	60	56
Drug target [TTD] *	29	30	30	30	30	30	31	29	29	30	32	35

Pa, Pectobacterium atrosepticum SCRI1043; Pcc, *P. carotovorum* subsp. *carotovorum* PCC21; Pcb, *P. carotovorum* subsp. *brasiliense* BC1; Pco, *P. carotovorum* subsp. *odoriferum* BC S7; Pca, *P. carotovorum* subsp. *actinidiae* KKH3; Par, *P. aroidearum* PC1; Pp, *P. parmentieri* SCC3193; Pw, *P. wasabiae* CFBP 3304; Pb, *P. betavasculorum* NCPPB 2795; Ppo, *P. polaris* NIBIO1392; Ppe, *P. peruviense* IFB5232; Cpm, Candidatus Pectobacterium maceratum PB69; GC, Guanine-Cytosine; CDS, Coding Sequence Regions; EC number, Enzyme Commission Number; TTD, Therapeutic Target Database; * data retrieved from the bioinformatics PATRIC webserver; ^†^ non-complete and/or Draft genomes.

## Supplementary Materials

The following are available online at https://www.mdpi.com/2076-0817/8/4/247/s1.

## References

[1] Czajkowski R., Pérombelon M.C.M., van Veen J.A., van der Wolf J.M. (2011). Control of blackleg and tuber soft rot of potato caused by *Pectobacterium* and *Dickeya* species: A review. Plant Pathol..

[2] Davidsson P.R., Kariola T., Niemi O., Palva T. (2013). Pathogenicity of and plant immunity to soft rot pectobacteria. Front. Plant Sci..

[3] Toth I.K., Bell K.S., Holeva M.C., Birch P.R.J. (2003). Soft rot erwiniae: From genes to genomes. Mol. Plant Pathol..

[4] Ma B., Hibbing M.E., Kim H.-S., Reedy R.M., Yedidia I., Breuer J., Breuer J., Glasner J.D., Perna N.T., Kelman A. (2007). Host range and molecular phylogenies of the soft rot enterobacterial genera *Pectobacterium* and *Dickeya*. Phytopathology.

[5] Mansfield J., Genin S., Magori S., Citovsky V., Sriariyanum M., Ronald P., Dow M., Verdier V., Beer S.V., Machado M.A. (2012). Top 10 plant pathogenic bacteria in molecular plant pathology. Mol. Plant Pathol..

[6] Zhang H., Xu F., Wu Y., Hu H., Dai X. (2017). Progress of potato staple food research and industry development in China. J. Integr. Agric..

[7] Nabhan S., Boer S.H., Maiss E., Wydra K. (2012). Taxonomic relatedness between *Pectobacterium carotovorum* subsp. *carotovorum*, *Pectobacterium carotovorum* subsp. *odoriferum* and *Pectobacterium carotovorum* subsp. *brasiliense* subsp. nov.. J. Appl. Microbiol..

[8] Glasner J.D., Marquez-Villavicencio M., Kim H.-S., Jahn C.E., Ma B., Biehl B.S., Rissman A.I., Mole B., Yi X., Yang C.-H. (2008). Niche-specificity and the variable fraction of the *Pectobacterium* pan-genome. Mol. Plant Microbe Interact..

[9] Basset A., Tzou P., Lemaitre B., Boccard F. (2003). A single gene that promotes interaction of a phytopathogenic bacterium with its insect vector, *Drosophila melanogaster*. EMBO Rep..

[10] Hauben L., Moore E.R.B., Vauterin L., Steenackers M., Mergaert J., Verdonck L., Swings J. (1998). Phylogenetic position of phytopathogens within the *Enterobacteriaceae*. Syst. Appl. Microbiol..

[11] Gardan L., Gouy C., Christen R., Samson R. (2003). Elevation of three subspecies of *Pectobacterium carotovorum* to species level: *Pectobacterium atrosepticum* sp. nov., *Pectobacterium betavasculorum* sp. nov. and *Pectobacterium wasabiae* sp. nov.. Int. J. Syst. Evol. Microbiol..

[12] Duarte V., De Boer S.H., Ward L.J., Oliveira A.M.R. (2004). Characterization of atypical *Erwinia carotovora* strains causing blackleg of potato in Brazil. J. Appl. Microbiol..

[13] Samson R., Legendre J.B., Christen R., Saux M.F.-L., Achouak W., Gardan L. (2005). Transfer of *Pectobacterium chrysanthemi* (Burkholder et al. 1953) Brenner et al. 1973 and *Brenneria paradisiaca* to the genus *Dickeya* gen. nov. as *Dickeya chrysanthemi* comb. nov. and *Dickeya paradisiaca* comb. nov. and delineation of four novel species, *Dickeya dadantii* sp. nov., *Dickeya dianthicola* sp. nov., *Dickeya dieffenbachiae* sp. nov. and *Dickeya zeae* sp. nov.. Int. J. Syst. Evol. Microbiol..

[14] Brady C.L., Cleenwerck I., Venter S.N., Engelbeen K., De Vos P., Coutinho T.A. (2010). Emended description of the genus *Pantoea*, description of four species from human clinical samples, *Pantoea septica* sp. nov., *Pantoea eucrina* sp. nov., *Pantoea brenneri* sp. nov. and *Pantoea conspicua* sp. nov., and transfer of *Pectobacterium cypripedii* (Hori 1911) Brenner et al. 1973 emend. Hauben et al. 1998 to the genus as *Pantoea cypripedii* comb. nov.. Int. J. Syst. Evol. Microbiol..

[15] Koh Y.J., Kim G.H., Lee Y.S., Sohn S.H., Koh H.S., Kwon S., Heu S., Jung J.S. (2012). *Pectobacterium carotovorum* subsp. *actinidiae* subsp. nov., a new bacterial pathogen causing canker-like symptoms in yellow kiwifruit, *Actinidia chinensis*. N. Z. J. Crop Hortic. Sci..

[16] Nabhan S., De Boer S.H., Maiss E., Wydra K. (2013). *Pectobacterium aroidearum* sp. nov., a soft rot pathogen with preference for monocotyledonous plants. Int. J. Syst. Evol. Microbiol..

[17] Khayi S., Cigna J., Chong T.M., Quêtu-Laurent A., Chan K.-G., Hélias V., Faure D. (2016). Transfer of the potato plant isolates of *Pectobacterium wasabiae* to *Pectobacterium parmentieri* sp. nov.. Int. J. Syst. Evol. Microbiol..

[18] Dees M.W., Lysøe E., Rossmann S., Perminow J., Brurberg M.B. (2017). *Pectobacterium polaris* sp. nov., isolated from potato (*Solanum tuberosum*). Int. J. Syst. Evol. Microbiol..

[19] Waleron M., Misztak A., Waleron M., Franczuk M., Wielgomas B., Waleron K. (2018). Transfer of *Pectobacterium carotovorum* subsp. *carotovorum* strains isolated from potatoes grown at high altitudes to *Pectobacterium peruviense* sp. nov.. Syst. Appl. Microbiol..

[20] Shirshikov F.V., Korzhenkov A.A., Miroshnikov K.K., Kabanova A.P., Barannik A.P., Ignatov A.N., Miroshnikov K.A. (2018). Draft genome sequences of new genomospecies “*Candidatus* Pectobacterium maceratum” Strains, Which Cause Soft Rot in Plants. Genome Announc..

[21] Bell K.S., Sebaihia M., Pritchard L., Holden M.T.G., Hyman L.J., Holeva M.C., Thomson N.R., Bentley S.D., Churcher L.J.C., Mungall K. (2004). Genome sequence of the enterobacterial phytopathogen *Erwinia carotovora* subsp. *atroseptica* and characterization of virulence factors. Proc. Natl. Acad. Sci. USA.

[22] Charkowski A.O., Lind J., Rubio-Salazar I., Gross D.C., Lichens-Park A., Kole C. (2014). Genomics of plant-associated bacteria: The soft rot *Enterobacteriaceae*. Genomics of Plant-Associated Bacteria.

[23] Lee D.H., Lim J.-A., Lee J., Roh E., Jung K., Choi M., Oh C., Ryu S., Yun J., Heu S. (2013). Characterization of genes required for the pathogenicity of *Pectobacterium carotovorum* subsp. *carotovorum* Pcc21 in Chinese cabbage. Microbiology.

[24] Huang Y., Liu C., Wang H., Guan T., Liu L., Yu S. (2019). Bioinformatic analysis of the complete genome sequence of *Pectobacterium carotovorum* subsp. *brasiliense* BZA12 and candidate effector screening. J. Plant Pathol..

[25] Waleron M., Waleron K., Lojkowska E. (2014). Characterization of *Pectobacterium carotovorum* subsp. *odoriferum* causing soft rot of stored vegetables. Eur. J. Plant Pathol..

[26] Nykyri J., Niemi O., Koskinen P., Nokso-Koivisto J., Pasanen M., Broberg M., Plyusnin I., Törönen P., Holm L., Pirhonen M. (2012). Revised phylogeny and novel horizontally acquired virulence determinants of the model soft rot phytopathogen *Pectobacterium wasabiae* SCC3193. PLoS Pathog..

[27] Toth I.K., Pritchard L., Birch P.R.J. (2006). Comparative genomics reveals what makes an enterobacterial plant pathogen. Annu. Rev. Phytopathol..

[28] McGowan S.J., Sebaihia M., Porter L.E., Stewart G.S., Williams P., Bycroft B.W., Salmond G.P.C. (1996). Analysis of bacterial carbapenem antibiotic production genes reveals a novel β-lactam biosynthesis pathway. Mol. Microbiol..

[29] Chuang D., Chien Y., Wu H.-P. (2007). Cloning and expression of the *Erwinia carotovora* subsp. *carotovora* gene encoding the low-molecular-weight bacteriocin Carocin S1. J. Bacteriol..

[30] Chan Y., Wu H.-P., Chuang D. (2009). Extracellular secretion of Carocin S1 in *Pectobacterium carotovorum* subsp. *carotovorum* occurs via the type III secretion system integral to the bacterial flagellum. BMC Microbiol..

[31] Nguyen A.H., Tomita T., Hirota M., Sato T., Kamio Y. (1999). A simple purification method and morphology and component analyses for carotovoricin Er, a phage-tail-like bacteriocin from the plant pathogen *Erwinia carotovora* Er. Biosci. Biotechnol. Biochem..

[32] Itoh Y., Izaki K., Takahashi H. (1978). Purification and characterization of a bacteriocin from *Erwinia carotovora*. J. Gen. Appl. Microbiol..

[33] Park T.-H., Choi B.-S., Choi A.-Y., Choi I.-Y., Heu S., Park B.-S. (2012). Genome sequence of *pectobacterium carotovorum* subsp. *carotovorum* strain pcc21, a pathogen causing soft rot in Chinese cabbage. J. Bacteriol..

[34] Niemi O., Laine P., Koskinen P., Pasanen M., Pennanen V., Harjunpää H., Nykyri J., Holm L., Paulin L., Auvinen P. (2017). Genome sequence of the model plant pathogen *Pectobacterium carotovorum* SCC1. Stand. Genom. Sci..

[35] Cai H., Bai Y., Guo C. (2018). Comparative genomics of 151 plant-associated bacteria reveal putative mechanisms underlying specific interactions between bacteria and plant hosts. Genes Genom..

[36] McAdam P.R., Richardson E.J., Fitzgerald J.R. (2014). High-throughput sequencing for the study of bacterial pathogen biology. Curr. Opin. Microbiol..

[37] Xu J., Wang N. (2019). Where are we going with genomics in plant pathogenic bacteria?. Genomics.

[38] Klosterman S.J., Rollins J.R., Sudarshana M.R., Vinatzer B.A. (2016). Disease management in the genomics era—summaries of focus issue papers. Phytopathology.

[39] Bellieny-Rabelo D., Tanui C.K., Miguel N., Kwenda S., Shyntum D.Y., Moleleki L.N. (2018). Transcriptome and comparative genomics analyses reveal new functional insights on key determinants of pathogenesis and interbacterial competition in *Pectobacterium* and *Dickeya* spp.. Appl. Environ. Microbiol..

[40] Richter M., Rosselló-Móra R., Oliver Glöckner F., Peplies J. (2016). JSpeciesWS: A web server for prokaryotic species circumscription based on pairwise genome comparison. Bioinformatics.

[41] Auch A.F., von Jan M., Klenk H.-P., Göker M. (2010). Digital DNA-DNA hybridization for microbial species delineation by means of genome-to-genome sequence comparison. Stand. Genom. Sci..

[42] Meier-Kolthoff J.P., Auch A.F., Klenk H.-P., Göker M. (2013). Genome sequence-based species delimitation with confidence intervals and improved distance functions. BMC Bioinform..

[43] Edgar R.C. (2004). MUSCLE: A multiple sequence alignment method with reduced time and space complexity. BMC Bioinform..

[44] Kumar S., Stecher G., Li M., Knyaz C., Tamura K. (2018). MEGA X: Molecular evolutionary genetics analysis across computing platforms. Mol. Biol. Evol..

[45] Saitou N., Nei M. (1987). The neighbor-joining method: A new method for reconstructing phylogenetic trees. Mol. Biol. Evol..

[46] Tamura K., Nei M., Kumar S. (2004). Prospects for inferring very large phylogenies by using the neighbor-joining method. Proc. Natl. Acad. Sci. USA.

[47] Felsenstein J. (1985). Confidence limits on phylogenies: An approach using the bootstrap. Evolution.

[48] Wattam A.R., Abraham D., Dalay O., Disz T.L., Driscoll T., Gabbard J.L., Gillespie J.J., Gough R., Hix D., Kenyon R. (2014). PATRIC, the bacterial bioinformatics database and analysis resource. Nucleic Acids Res..

[49] Wattam A.R., Davis J.J., Assaf R., Boisvert S., Brettin T., Bun C., Conrad N., Dietrich E.M., Disz T., Gabbard J.L. (2017). Improvements to PATRIC, the all-bacterial bioinformatics database and analysis resource center. Nucleic Acids Res..

[50] Vesth T., Lagesen K., Acar Ö., Ussery D. (2013). CMG-Biotools, a free workbench for basic comparative microbial genomics. PLoS ONE.

[51] Alikhan N.-F., Petty N.K., Ben Zakour N.L., Beatson S.A. (2011). BLAST Ring Image Generator (BRIG): Simple prokaryote genome comparisons. BMC Genom..

[52] Altschul S.F., Gish W., Miller W., Myers E.W., Lipman D.J. (1990). Basic local alignment search tool. J. Mol. Biol..

[53] Ussery D.W., Wassenaar T.M., Borini S. (2009). Computing for Comparative Microbial Genomics: Bioinformatics for Microbiologists.

[54] Bertelli C., Laird M.R., Williams K.P., Lau B.Y., Hoad G., Winsor G.L., Brinkman F.S., Simon Fraser University Research Computing Group (2017). IslandViewer 4: Expanded prediction of genomic islands for larger-scale datasets. Nucleic Acids Res..

[55] Bertelli C., Brinkman F.S.L. (2018). Improved genomic island predictions with IslandPath-DIMOB. Bioinformatics.

[56] Langille M.G., Hsiao W.W., Brinkman F.S. (2008). Evaluation of genomic island predictors using a comparative genomics approach. BMC Bioinform..

[57] Hudson C.M., Lau B.Y., Williams K.P. (2015). Islander: A database of precisely mapped genomic islands in tRNA and tmRNA genes. Nucleic Acids Res..

[58] Blin K., Wolf T., Chevrette M.G., Lu X., Schwalen C.J., Kautsar S.A., Suarez Duran H.G., de los Santos E.L.C., Kim H.U., Nave M. (2017). AntiSMASH 4.0—Improvements in chemistry prediction and gene cluster boundary identification. Nucleic Acids Res..

[59] Van Heel A.J., de Jong A., Song C., Viel J.H., Kok J., Kuipers O.P. (2018). BAGEL4: A user-friendly web server to thoroughly mine RiPPs and bacteriocins. Nucleic Acids Res..

[60] Sullivan M.J., Petty N.K., Beatson S.A. (2011). Easyfig: A genome comparison visualizer. Bioinformatics.

[61] Couvin D., Bernheim A., Toffano-Nioche C., Touchon M., Michalik J., Néron B., Rocha E.P.C., Vergnaud G., Gautheret D., Pourcel C. (2018). CRISPRCasFinder, an update of CRISRFinder, includes a portable version, enhanced performance and integrates search for Cas proteins. Nucleic Acids Res..

[62] Biswas A., Staals R.H.J., Morales S.E., Fineran P.C., Brown C.M. (2016). CRISPRDetect: A flexible algorithm to define CRISPR arrays. BMC Genom..

[63] Uddin A. (2017). Codon Usage Bias: A tool for understanding molecular evolution. J. Proteom. Bioinform..

[64] Faye P., Bertrand C., Pédron J., Barny M.-A. (2018). Draft genomes of “*Pectobacterium peruviense*” strains isolated from fresh water in France. Stand. Genom. Sci..

[65] Charkowski A., Blanco C., Condemine G., Expert D., Franza T., Hayes C., Hugouvieux-Cotte-Pattat N., Solanilla E.L., Low D., Moleleki L. (2012). The role of secretion systems and small molecules in soft-rot *Enterobacteriaceae* pathogenicity. Annu. Rev. Phytopathol..

[66] Itoh Y., Izaki K., Takahashi H. (1980). Simultaneous synthesis of pectin lyase and carotovoricin induced by mitomycin c, nalidixic acid or ultraviolet light irradiation in *Erwinia carotovora*. Agric. Biol. Chem..

[67] Yamada K., Hirota M., Niimi Y., Nguyen H.A., Takahara Y., Kamio Y., Kaneko J. (2006). Nucleotide sequences and organization of the genes for carotovoricin (Ctv) from *Erwinia carotovora* indicate that Ctv evolved from the same ancestor as *Salmonella typhi* prophage. Biosci. Biotechnol. Biochem..

[68] Chan Y.-C., Wu J.-L., Wu H.-P., Tzeng K.-C., Chuang D.-Y. (2011). Cloning, purification, and functional characterization of Carocin S2, a ribonuclease bacteriocin produced by *Pectobacterium carotovorum*. BMC Microbiol..

[69] Roh E., Park T.-H., Kim M.-I., Lee S., Ryu S., Oh C.-S., Rhee S., Kim D.-H., Park B.-S., Heu S. (2010). Characterization of a new bacteriocin, Carocin D, from *Pectobacterium carotovorum* subsp. *carotovorum* Pcc21. Appl. Environ. Microbiol..

[70] Delepelaire P. (2004). Type I secretion in gram-negative bacteria. Biochim. Biophys. Acta Mol. Cell Res..

[71] Pérez-Mendoza D., Coulthurst S.J., Humphris S., Campbell E., Welch M., Toth I.K., Salmond G.P.C. (2011). A multi-repeat adhesin of the phytopathogen, *Pectobacterium atrosepticum*, is secreted by a Type I pathway and is subject to complex regulation involving a non-canonical diguanylate cyclase: Complex regulation of a Pectobacterium type I-secreted adhesin. Mol. Microbiol..

[72] Jha G., Rajeshwari R., Sonti R.V. (2005). Bacterial type two secretion system secreted proteins: Double-edged swords for plant pathogens. Mol. Plant Microbe Interact..

[73] Douzi B., Filloux A., Voulhoux R. (2012). On the path to uncover the bacterial type II secretion system. Phil. Trans. R. Soc. B.

[74] Christie P.J., Whitaker N., González-Rivera C. (2014). Mechanism and structure of the bacterial type IV secretion systems. Biochim. Biophys. Acta Mol. Cell Res..

[75] Trokter M., Waksman G. (2018). Translocation through the conjugative type IV secretion system requires unfolding of its protein substrate. J. Bacteriol..

[76] Van Ulsen P., ur Rahman S., Jong W.S.P., Daleke-Schermerhorn M.H., Luirink J. (2014). Type V secretion: From biogenesis to biotechnology. Biochim. Biophys. Acta Mol. Cell Res..

[77] Bernard C.S., Brunet Y.R., Gavioli M., Lloubes R., Cascales E. (2011). Regulation of type VI secretion gene clusters by 54 and cognate enhancer binding proteins. J. Bacteriol..

[78] Pukatzki S., Ma A.T., Sturtevant D., Krastins B., Sarracino D., Nelson W.C., Heidelberg J.F., Mekalanos J.J. (2006). Identification of a conserved bacterial protein secretion system in *Vibrio cholerae* using the *Dictyostelium* host model system. Proc. Natl. Acad. Sci. USA.

[79] Mattinen L., Nissinen R., Riipi T., Kalkkinen N., Pirhonen M. (2007). Host-extract induced changes in the secretome of the plant pathogenic bacterium *Pectobacterium atrosepticum*. Proteomics.

[80] Cianfanelli F.R., Alcoforado Diniz J., Guo M., De Cesare V., Trost M., Coulthurst S.J. (2016). VgrG and PAAR proteins define distinct versions of a functional type VI secretion system. PLoS Pathog..

[81] Mattinen L., Somervuo P., Nykyri J., Nissinen R., Kouvonen P., Corthals G., Auvinen P., Aittamaa M., Valkonen J.P.T., Pirhonen M. (2008). Microarray profiling of host-extract-induced genes and characterization of the type VI secretion cluster in the potato pathogen *Pectobacterium atrosepticum*. Microbiology.

[82] Maier B., Wong G.C.L. (2015). How bacteria use type IV pili machinery on surfaces. Trends Microbiol..

[83] Ben Fekih I., Zhang C., Li Y.P., Zhao Y., Alwathnani H.A., Saquib Q., Rensing C., Cervantes C. (2018). Distribution of arsenic resistance genes in prokaryotes. Front. Microbiol..

[84] Mosbahi K., Wojnowska M., Albalat A., Walker D. (2018). Bacterial iron acquisition mediated by outer membrane translocation and cleavage of a host protein. Proc. Natl. Acad. Sci. USA.

[85] Ishimaru C.A., Loper J.E. (1992). High-affinity iron uptake systems present in *Erwinia carotovora* subsp. *carotovora* include the hydroxamate siderophore aerobactin. J. Bacteriol..

[86] Corbett M., Virtue S., Bell K., Birch P., Burr T., Hyman L., Lilley K., Poock S., Toth I., Salmond G. (2005). Identification of a new quorum-sensing-controlled virulence factor in *Erwinia carotovora* subsp. *atroseptica* secreted via the type II targeting pathway. Mol. Plant Microbe Interact..

[87] Mattinen L., Tshuikina M., Mäe A., Pirhonen M. (2004). Identification and characterization of Nip, Necrosis-inducing virulence protein of *Erwinia carotovora* subsp. *carotovora*. Mol. Plant Microbe Interact..

[88] Urbany C., Neuhaus H.E. (2008). Citrate uptake into *Pectobacterium atrosepticum* is critical for bacterial virulence. Mol. Plant Microbe Interact..

[89] Shao N., Huang H., Meng K., Luo H., Wang Y., Yang P., Yao B. (2008). Cloning, expression, and characterization of a new phytase from the phytopathogenic bacterium *Pectobacterium wasabiae* DSMZ 18074. J. Microbiol. Biotechnol..

[90] Welte C.U., Rosengarten J.F., de Graaf R.M., Jetten M.S.M. (2016). SaxA-Mediated isothiocyanate metabolism in phytopathogenic pectobacteria. Appl. Environ. Microbiol..

[91] Jiang H., Jiang M., Yang L., Yao P., Ma L., Wang C., Wang H., Qian G., Hu B., Fan J. (2017). The ribosomal protein RplY is required for *Pectobacterium carotovorum* virulence and is induced by *Zantedeschia elliotiana* extract. Phytopathology.

[92] Del Pilar Marquez-Villavicencio M., Weber B., Witherell R.A., Willis D.K., Charkowski A.O. (2011). The 3-hydroxy-2-butanone pathway is required for *Pectobacterium carotovorum* pathogenesis. PLoS ONE.

[93] McGowan S., Sebaihia M., Jones S., Yu B., Bainton N., Chan P.F., Bycroft B., Stewart G.S.A.B., Williams P., Salmond G.P.C. (1995). Carbapenem antibiotic production in *Erwinia carotovora* is regulated by CarR, a homologue of the LuxR transcriptional activator. Microbiology.

[94] Haque M.M., Oliver M.M.H., Nahar K., Alam M.Z., Hirata H., Tsuyumu S. (2017). CytR Homolog of *Pectobacterium carotovorum* subsp. *carotovorum* controls air-liquid biofilm formation by regulating multiple genes involved in cellulose production, c-di-GMP signaling, motility, and type III secretion system in response to nutritional and environmental signals. Front. Microbiol..

[95] Parker W.L., Rathnum M.L., Wells J.J.S., Trejo W.H., Principe P.A., Sykes R.B. (1982). SQ 27, 860, a simple carbapenem produced by species of *Serratia* and *Erwinia*. J. Antibiot..

[96] McGowan S.J., Sebaihia M., O’Leary S., Williams P., Salmond G.P.C. (1997). Analysis of the carbapenem gene cluster of *Erwinia carotovora*: Definition of the antibiotic biosynthetic genes and evidence for a novel β-lactam resistance mechanism. Mol. Microbiol..

[97] Mavrodi D.V., Peever T.L., Mavrodi O.V., Parejko J.A., Raaijmakers J.M., Lemanceau P., Mazurier S., Heide L., Blankenfeldt W., Weller D.M. (2010). Diversity and evolution of the phenazine biosynthesis pathway. Appl. Environ. Microbiol..

[98] Grinter R., Milner J., Walker D. (2012). Ferredoxin containing bacteriocins suggest a novel mechanism of iron uptake in *Pectobacterium* spp.. PLoS ONE.

[99] Makarova K.S., Wolf Y.I., Alkhnbashi O.S., Costa F., Shah S.A., Saunders S.J., Barrangou R., Brouns S.J.J., Charpentier E., Haft D.H. (2015). An updated evolutionary classification of CRISPR-Cas systems. Nat. Rev. Microbiol..

[100] Tambong J.T. (2017). Comparative genomics of *Clavibacter michiganensis* subspecies, pathogens of important agricultural crops. PLoS ONE.

[101] Wu X., Monchy S., Taghavi S., Zhu W., Ramos J., van der Lelie D. (2011). Comparative genomics and functional analysis of niche-specific adaptation in *Pseudomonas putida*. FEMS Microbiol. Rev..

[102] Kozobay-Avraham L. (2006). Involvement of DNA curvature in intergenic regions of prokaryotes. Nucleic Acids Res..

[103] Duan C., Huan Q., Chen X., Wu S., Carey L.B., He X., Qian W. (2018). Reduced intrinsic DNA curvature leads to increased mutation rate. Genome Biol..

[104] Wojcik E.A., Brzostek A., Bacolla A., Mackiewicz P., Vasquez K.M., Korycka-Machala M., Jaworski A., Dziadek J. (2012). Direct and inverted repeats elicit genetic instability by both exploiting and eluding DNA double-strand break repair systems in *Mycobacteria*. PLoS ONE.

[105] Kim H.-S., Ma B., Perna N.T., Charkowski A.O. (2009). Phylogeny and virulence of naturally occurring type III secretion system-deficient *Pectobacterium* strains. Appl. Environ. Microbiol..

[106] Kim H.-S., Thammarat P., Lommel S.A., Hogan C.S., Charkowski A.O. (2011). *Pectobacterium carotovorum* elicits plant cell death with DspE/F but the *P. carotovorum* DspE does not suppress callose or induce expression of plant genes early in plant–microbe interactions. Mol. Plant Microbe Interact..

[107] Rojas C.M., Ham J.H., Deng W.-L., Doyle J.J., Collmer A. (2002). HecA, a member of a class of adhesins produced by diverse pathogenic bacteria, contributes to the attachment, aggregation, epidermal cell killing, and virulence phenotypes of *Erwinia chrysanthemi* EC16 on *Nicotiana clevelandii* seedlings. Proc. Natl. Acad. Sci. USA.

[108] Poole S.J., Diner E.J., Aoki S.K., Braaten B.A., t’Kint de Roodenbeke C., Low D.A., Hayes C.S. (2011). Identification of functional toxin/immunity genes linked to contact-dependent growth inhibition (CDI) and rearrangement hotspot (Rhs) systems. PLoS Genet..

[109] Gallique M., Bouteiller M., Merieau A. (2017). The type VI secretion system: A dynamic system for bacterial communication?. Front. Microbiol..

[110] Liu H., Coulthurst S.J., Pritchard L., Hedley P.E., Ravensdale M., Humphris S., Burr T., Takle G., Brurberg M.-B., Birch P.R.J. (2008). Quorum sensing coordinates brute force and stealth modes of infection in the plant pathogen *Pectobacterium atrosepticum*. PLoS Pathog..

[111] Giltner C.L., Nguyen Y., Burrows L.L. (2012). Type IV pilin proteins: Versatile molecular modules. Microbiol. Mol. Biol. Rev..

[112] Carbonnelle E., Hélaine S., Prouvensier L., Nassif X., Pelicic V. (2004). Type IV pilus biogenesis in *Neisseria meningitidis*: PilW is involved in a step occurring after pilus assembly, essential for fibre stability and function. Mol. Microbiol..

[113] Koo J., Tammam S., Ku S.-Y., Sampaleanu L.M., Burrows L.L., Howell P.L. (2008). PilF is an outer membrane lipoprotein required for multimerization and localization of the *Pseudomonas aeruginosa* type IV pilus secretin. J. Bacteriol..

[114] Sauvonnet N., Gounon P., Pugsley A.P. (2000). PpdD type IV pilin of *Escherichia coli* K-12 can be assembled into pili in *Pseudomonas aeruginosa*. J. Bacteriol..

[115] Giltner C.L., van Schaik E.J., Audette G.F., Kao D., Hodges R.S., Hassett D.J., Irvin R.T. (2006). The *Pseudomonas aeruginosa* type IV pilin receptor binding domain functions as an adhesin for both biotic and abiotic surfaces. Mol. Microbiol..

[116] Nykyri J., Mattinen L., Niemi O., Adhikari S., Kõiv V., Somervuo P., Fang X., Auvinen P., Mäe A., Palva E.T. (2013). Role and regulation of the Flp/Tad pilus in the virulence of *Pectobacterium atrosepticum* SCRI1043 and *Pectobacterium wasabiae* SCC3193. PLoS ONE.

[117] Panda P., Vanga B.R., Lu A., Fiers M., Fineran P.C., Butler R., Armstrong K., Ronson C.W., Pitman A.R. (2016). *Pectobacterium atrosepticum* and *Pectobacterium carotovorum* harbor distinct, independently acquired integrative and conjugative elements encoding coronafacic acid that enhance virulence on potato stems. Front. Microbiol..

[118] Bender C.L., N-Chaidez F.A., Gross D.C. (1999). *Pseudomonas syringae* phytotoxins: Mode of action, regulation, and biosynthesis by peptide and polyketide synthetases. Microbiol. Mol. Biol. Rev..

[119] Evans T.J., Ind A., Komitopoulou E., Salmond G.P.C. (2010). Phage-selected lipopolysaccharide mutants of *Pectobacterium atrosepticum* exhibit different impacts on virulence. J. Appl. Microbiol..

[120] Ragunath C., Shanmugam M., Bendaoud M., Kaplan J.B., Ramasubbu N. (2012). Effect of a biofilm-degrading enzyme from an oral pathogen in transgenic tobacco on the pathogenicity of *Pectobacterium carotovorum* subsp. *carotovorum*. Plant Pathol..

[121] Grinter R., Josts I., Mosbahi K., Roszak A.W., Cogdell R.J., Bonvin A.M.J.J., Milner J.J., Kelly S.M., Byron O., Smith B.O. (2016). Structure of the bacterial plant-ferredoxin receptor FusA. Nat. Commun..

[122] Grinter R., Hay I.D., Song J., Wang J., Teng D., Dhanesakaran V., Wilksch J.J., Davies M.R., Littler D., Beckham S.A. (2018). FusC, a member of the M16 protease family acquired by bacteria for iron piracy against plants. PLoS Biol..

[123] Pirhonen M., Saarilahti H., Karlsson M.-B., Palva E.T. (1991). Identification of pathogenicity determinants of *Erwinia carotovora* subsp. *carotovora* by transposon mutagenesis. Mol. Plant-Microbe Interact..

[124] Pemberton C.L., Whitehead N.A., Sebaihia M., Bell K.S., Hyman L.J., Harris S.J., Matlin A.J., Robson N.D., Birch P.R.J., Carr J.P. (2005). Novel quorum-sensing-controlled genes in *Erwinia carotovora* subsp. *carotovora*: Identification of a fungal elicitor homologue in a soft-rotting bacterium. Mol. Plant Microbe Interact..

[125] Fitzpatrick D.A. (2009). Lines of evidence for horizontal gene transfer of a phenazine producing operon into multiple bacterial species. J. Mol. Evol..

[126] Zhanel G.G., Wiebe R., Dilay L., Thomson K., Rubinstein E., Hoban D.J., Noreddin A.M., Karlowsky J.A. (2007). Comparative review of the carbapenems. Drugs.

[127] Wu M.-C., Chen Y.-C., Lin T.-L., Hsieh P.-F., Wang J.-T. (2012). Cellobiose-specific phosphotransferase system of *Klebsiella pneumoniae* and its importance in biofilm formation and virulence. Infect. Immun..

[128] Liu Y., Yoo B.B., Hwang C.-A., Suo Y., Sheen S., Khosravi P., Huang L. (2017). LMOf2365_0442 encoding for a fructose specific PTS permease IIA may be required for virulence in *L. monocytogenes* Strain F2365. Front. Microbiol..

[129] Koonin E.V., Makarova K.S., Zhang F. (2017). Diversity, classification and evolution of CRISPR-Cas systems. Curr. Opin. Microbiol..

[130] Rath D., Amlinger L., Rath A., Lundgren M. (2015). The CRISPR-Cas immune system: Biology, mechanisms and applications. Biochimie.

[131] Godde J.S., Bickerton A. (2006). The repetitive DNA elements called CRISPRs and their associated genes: Evidence of horizontal transfer among prokaryotes. J. Mol. Evol..

[132] Seed K.D., Lazinski D.W., Calderwood S.B., Camilli A. (2013). A bacteriophage encodes its own CRISPR/Cas adaptive response to evade host innate immunity. Nature.

[133] Watson B.N.J., Staals R.H.J., Fineran P.C. (2018). CRISPR-Cas-Mediated phage resistance enhances horizontal gene transfer by transduction. mBio.

[134] Koonin E.V., Makarova K.S. (2013). CRISPR-Cas: Evolution of an RNA-based adaptive immunity system in prokaryotes. RNA Biol..

[135] Zhang Q., Doak T.G., Ye Y. (2014). Expanding the catalog of cas genes with metagenomes. Nucleic Acids Res..

[136] Labbate M., Orata F.D., Petty N.K., Jayatilleke N.D., King W.L., Kirchberger P.C., Allen C., Mann G., Mutreja A., Thomson N.R. (2016). A genomic island in *Vibrio cholerae* with VPI-1 site-specific recombination characteristics contains CRISPR-Cas and type VI secretion modules. Sci. Rep..

[137] Haft D.H., Selengut J., Mongodin E.F., Nelson K.E. (2005). A guild of 45 CRISPR-associated (cas) protein families and multiple CRISPR/Cas subtypes exist in prokaryotic genomes. PLoS Comp. Biol..

[138] Zhang Q., Ye Y. (2017). Not all predicted CRISPR–Cas systems are equal: Isolated cas genes and classes of CRISPR like elements. BMC Bioinform..

