# Supplementary material for: Genome-Wide Analyses Revealed Remarkable Heterogeneity in Pathogenicity Determinants, Antimicrobial Compounds, and CRISPR-Cas Systems of Complex Phytopathogenic Genus Pectobacterium
Source: Pathogens. 2019 Nov 20;8(4):247. doi: 10.3390/pathogens8040247 (PMC6963963; doi:10.3390/pathogens8040247)
Supplement: Supplementary file 1 [file pathogens-08-00247-s001.zip › pathogens-633436supplementary/pathogens-633436 supplementary titles.pdf]

Supplement Figures

Figure S1. Genome atlases of complete *Pectobacterium* genomes. (**A**) Genome atlas of *P. atrosepticum* SCRI1043, (**B**) *P. carotovorum* subsp. *carotovorum* PCC21, (**C**) *P. carotovorum* subsp. *brasiliense* BC1, (**D**) *P. carotovorum* subsp. *odoriferum* BCS7, (**E**) *P. aroidearum* PC1, (**F**) *P. parmentieri* SCC3193, (**G**) *P. wasabiae* CFBP 3304, and (**H**) *P. polaris* NIBIO1392. The DNA structural atlases were created using the CMG-Biotools package for each of the eight complete genomes within *Pectobacterium* genus**.** DNA, RNA and gene annotation data were retrieved from NCBI GenBank. The complete nucleotide sequence was used as an input data. Atlases displaying a specific DNA feature were depicted in each circle lane. From the innermost layer to the outermost layer: genome size(axis), AT percentagewise (red indicates high AT), GC skew (blue indicates more Guanine content), inverted and direct repeats (where blue and red lines indicate direct and indirect repeats, respectively), position preference (green to pink color scale), stacking energy (green to red color scale) and intrinsic curvature (yellow and blue color scale). Orange arrows are pointing out changes in the GC skew. Light blue arrows pointed out the rRNA regions within each chromosome. Red dark arrows indicate regions where DNA appeared to exhibit distinct structural features from the genome.

Figure S2. Position specific to nucleotide triplet usage among the *Pectobacterium* species. The bar diagrams depict the average number of each nucleotide on each position calculated by total amount of codons. The bias score in the third position is displayed for each *Pectobacterium* species. The frequency of nucleotides in the third position was determined as sum of guanine and cytosine (GC) subtracted by the sum of adenine and thymine (AT). Hence, 100% of GC at third codon position is defined as +1 values; in contrast, -1 values represent 100% of AT. The bar colors corresponded to: Adenine (green), Thymine (red), Guanine (black) and Cytosine (blue).

Figure S3. Rose plots that illustrate the percentage of codon usage in each *Pectobacterium* species. The analysis was computed in the CMG-Biotools package. The range of codon percentagewise is depicted in the right of each rose plot. Each diagram is associated with the name of the corresponding species.

Figure S4. Rose plots that illustrate the percentage of amino acid usage in each *Pectobacterium* species. The analysis was computed with CMG-Biotools package. The percent range of amino acid is depicted in the right of each rose plot. Each diagram was labeled with the corresponding species name.

Figure S5. Linear sequence comparison of type I secretion system (T1SS) essential for secretion of a proteinaceous multi-repeat adhesin (MRP) among the *Pectobacterium* species. The arrow position indicates forward/reverse directionality of gene orientation Arrow color depicts a specific gene composition within the T1SS – MRP cluster. Gene names are provided at the top and bottom of the linear graph; the locus-tag was used for hypothetical or genes with no names. A pairwise alignment between the linear sequences illustrated based on BLAST algorithm with cut-off values from 69% to 100%. Regions with higher nucleotide identity were displayed with a gray shading color. Legend acronyms are as follows: MRP (multi-repeat adhesin protein).

Figure S6. Linear sequence comparison of type II secretion system (T2SS) among the *Pectobacterium* species. Core composition of T2SS is indicated in parenthesis on the top of the figure. The arrow position indicates forward/reverse directionality of gene orientation. Arrow color depicted specific gene composition within the T2SS. Gene names are provided at the top and bottom of the linear; the locus-tag was used for hypothetical or genes with no names. A BLAST generated pairwise alignment between the linear sequences illustrated with cut-off values from 77% to 100%. Regions with higher nucleotide identity were displayed with a shaded. Acronyms used in the legend: PCWDE (Plant Cell Wall-Degrading Enzymes), *pehX* (polygalacturonase).

Figure S7. Linear sequence comparison of the VirB/D4 type IV secretion system (T4SS) of *Agrobacterium tumefaciens* across the *Pectobacterium* genus. The arrow position indicates forward/reverse directionality of gene

orientation. Arrow color depicts specific gene composition within the VirB/D4 T4SS. Gene names are provided at the top and bottom of the linear graph; the locus-tag was used for hypothetical or genes with no names. A BLAST generated pairwise alignment between the linear sequences is illustrated with cut-off values from 65% to 100%. Regions reporting higher nucleotide identity were displayed with a gray shading color. The two copies of the VirB/D4 T4SS in *P. carotovorum* subsp. *brasiliense* strain BC1 were highlighted in a green square.

Figure S8. Linear visualization of the unreported type V secretion system (T5SS) found in *Pectobacterium* genus. Colors in arrows depicted specific gene composition within the T5SS. Gene names are described inside the arrows while their corresponding name are provided at the bottom of the linear graph. Locus tag of each gene within this unreported T5SS is described for each *Pectobactrerium* species in a table beneath the linear graph. (X) means that that gene is not present.

Figure S9. Linear sequence comparison of the type VI secretion system (T6SS) across the *Pectobacterium* genus. The arrows position depicted the orientation of genes (forward and reverse). Colors in arrows depict specific gene composition within the T6SS. Gene names are provided at the top and bottom of the linear graph; the locus-tag was used for hypothetical or genes with no names. A BLAST generated pairwise alignment between the linear sequences is illustrated with cut-off values from 67% to 100%. Regions with higher nucleotide identity are displayed with a gray shading color. Lime-green square in *P. c.* subsp. *odoriferum* is indicating the extra set of genes only present in this species. Acronyms used in the legend: CDS (coding sequences), PAAR (proline-alanine-alanine-arginine repeat protein), Hcp (haemolysin-coregulated proteins), Rhs (rearrangement hot spots), VgrG (valine glycine repeat G protein), Vas (Virulence-associated secretion proteins) and *imp* (nodulation impairment locus).

Figure S10. Linear sequence comparison of type IV pilus biogenesis cluster within the *Pectobacterium* genus. The arrows position represented the orientation of genes (forward and reverse). Gene names are provided at the top and bottom of the linear graph. A BLAST generated pairwise alignment between the linear sequences is illustrated with cut-off values from 86% to 100%. Regions with higher nucleotide identity are displayed with a gray shading color. Acronym used in the legend: *pil* (pili or pilus genes).

Figure S11. Linear sequence comparison of the Flp/Tad (fimbrial low-molecular-weight protein – tight adherence protein) pilus cluster in *Pectobacterium*. Arrow color specific gene composition within the Flp/Tad cluster. Gene names are provided at the top and bottom of the linear graph; the locus-tag was used for hypothetical or genes with no names. A pairwise alignment between the linear sequences illustrated based on BLAST algorithm with cut-off values from 84% to 100%. Regions with higher nucleotide identity are displayed with a gray shading color. Acronyms used in the legend: *flp* (fimbrial low-molecular-weight protein), *rcp* (rough colony protein) and *tad* (tight adherence protein).

Figure S12. Linear sequence comparison of the phytotoxin coronofacic acid (*cfa*) within *Pectobacterium*. Arrow color depicted the specific gene composition within the *cfa* cluster. Gene names are provided at the top and bottom of the linear graph. A pairwise alignment between the linear sequences illustrated based on BLAST algorithm with cut-off values from 67% to 100%. Regions reporting higher nucleotide identity are displayed with a gray shading color. Acronyms used in the legend: *cfa* (coronofacic acid) and *cfl* (coronafacate ligase).

Figure S13. Linear sequence comparison of the arsenic (*ars*) resistance cluster of *P. parmentieri* with other *Pectobacterium* species. Arrow color depicted specific gene composition within the *ars* cluster. Gene names are provided at the top and bottom of the linear graph; the locus-tag was used for hypothetical or genes with no names. A pairwise alignment between the linear sequences illustrated based on BLAST algorithm with cut-off values from 70% to 100%. Regions reporting higher nucleotide identity are displayed with a gray shading color.

Figure S14. Linear sequence comparison of the capsular polysaccharide (*cps*) cluster among the *Pectobacterium* species. Arrow color depicted specific gene composition within the *cps* cluster. Gene names are provided at the top and bottom of the linear graph; the locus-tag was used for hypothetical or genes with no names. A pairwise alignment between the linear sequences illustrated based on BLAST algorithm with cut-off values from 95% to 100%. Regions reporting higher nucleotide identity are displayed with a gray shading color.

Figure S15. Linear sequence comparison of the Enterobacterial Common Antigen (ECA) cluster among the *Pectobacterium* species. Arrow color depicted specific gene composition within the ECA cluster. Gene names are provided at the top and bottom of the linear graph; the locus-tag was used for hypothetical or genes with no names. A pairwise alignment between the linear sequences illustrated based on BLAST algorithm with cut-off values from 89% to 100%. Regions with higher nucleotide identity are displayed with a gray shading color.

Figure S16. Linear sequence comparison of the Exopolysaccharide (EPS) O-antigen cluster among the *Pectobacterium* species. Arrow color depicted specific gene composition within the EPS-O antigen cluster. Gene names are provided at the top and bottom of the linear graph; the locus-tag was used for hypothetical or genes with no names. A pairwise alignment between the linear sequences illustrated based on BLAST algorithm with cut-off values from 63% to 100%. Regions with higher nucleotide identity are displayed with a gray shading color. Yellow rectangle marked in red is highlighting the distinct rearrangements and variable region of the EPS-O antigen cluster across the different *Pectobacterium* species.

Figure S17. Linear sequence comparison of the lipo-oligo/polysaccharide cluster (LOS/LPS) among the *Pectobacterium* species. Arrow color depicted specific gene composition within the LOS/LPS cluster. Gene names are provided at the top and bottom of the linear graph; the locus-tag was used for hypothetical or genes with no names. A pairwise alignment between the linear sequences illustrated based on BLAST algorithm with cut-off values from 65% to 100%. Regions with higher nucleotide identity are displayed with a gray shading color.

Figure S18. Linear sequence comparison of the 3-hydroxy-2-butanone (3H2B) among the *Pectobacterium* species. Arrow color depicted specific gene composition within the 3H2B operon. Gene names are provided at the top and bottom of the linear graph; the locus-tag was used for hypothetical or genes with no names. A pairwise alignment between the linear sequences illustrated based on BLAST algorithm with cut-off values from 89% to 100%. Regions with higher nucleotide identity are displayed with a gray shading color. Acronyms used in the legend: CDS (coding sequences), tRNA (transfer ribonucleic acid), *budR* (transcriptional regulator BudR), *budA* (α-acetolactate decarboxylase), *budB* (α-acetolactate synthase) and *budC* (butanediol dehydrogenase).

Figure S19. Linear sequence comparison of the carotovoricin (*ctv*) biosynthetic cluster among the *Pectobacterium* species. Position of the T1SS (*prtDEF* operon) and carotovoricin cluster are indicated in parenthesis at the top of the figure. Arrow color depicted depict specific gene composition within the *ctv* cluster. Gene names are provided at the top and bottom of the linear graph; the locus-tag was used for hypothetical or genes with no names. A pairwise alignment between the linear sequences illustrated based on BLAST algorithm with cut-off values from 66% to 100%. Yellow rectangles are highlighting the genomic region that were found elsewhere in the chromosomes of *P. atrosepticum* and *P. betavasculorum*. Light blue arrows are indicating the genes unique to *P. betavasculorum* strain NCPPB 2795.

Figure S20. Linear sequence comparison of the carbapenem (*car*) biosynthetic cluster among the *Pectobacterium* species. Arrow color depicted specific gene composition within the *car* operon. Gene names are provided at the top and bottom of the linear graph; the locus-tag was used for hypothetical or genes with no names. A pairwise alignment between the linear sequences illustrated based on BLAST algorithm with cut-off values from 81% to 100%. Regions with higher nucleotide identity are displayed with a gray shading color. Rectangles marked in blue and decorated with light blue indicated the three *car* genes found in some *Pectobacterium* species.

Figure S21. Linear sequence comparison of the phenazine (*ehp*) biosynthetic cluster among the *Pectobacterium* species. Arrow color depicted specific gene composition within the *ehp* operon. Gene names are provided at the top and bottom of the linear graph; the locus-tag was used for hypothetical or genes with no names. A pairwise alignment between the linear sequences illustrated based on BLAST algorithm with cut-off values from 93% to 100%. Acronym used in the legend: *ehp* (phenazine synthesis genes).

Figure S22. Linear visualization of the novel cellobiose-specific phosphotransferase system (PTS) in *Pectobacterium parmentieri*. The blue line over the linear graph is representing the GC content of the 4.54 kb size of the PTS cluster. Arrow color depicted specific gene composition within the PTS. Core composition of the Enzyme permease complex (EII) is indicated in parenthesis on the top of the figure. Names of coding-gene products are provided at the bottom of the linear graph. Locus tag of each gene within the PTS is described for the six *P. parmentieri* strains in a table beneath the linear graph.

Supplement Tables

Provided with attachements
