# Supplementary material for: Genome-Wide Analyses Revealed Remarkable Heterogeneity in Pathogenicity Determinants, Antimicrobial Compounds, and CRISPR-Cas Systems of Complex Phytopathogenic Genus Pectobacterium
Source: Pathogens. 2019 Nov 20;8(4):247. doi: 10.3390/pathogens8040247 (PMC6963963; doi:10.3390/pathogens8040247)
Supplement: Supplementary file 1 [file pathogens-08-00247-s001.zip › pathogens-633436supplementary/Supplementary Files/TABLE S1.docx]

**Table S1.** Detailed description of all *Pectobacterium* genomes used for Phylogenomics analysis.

| **Species Name** | **Strain ID** | **GenBank Accession Numbers** | | **Genome Status** | **Host and/or Isolation Source** | **Location & Isolation Year** | **Sequencing Year** | **Sequencing Platform** | **Genome Assembler** |
| --- | --- | --- | --- | --- | --- | --- | --- | --- | --- |
|  |  | **RefSeq** | **INSDC** |  |  |  |  |  |  |
| ***Pectobacterium atrosepticum*** | SCRI1043* | NC_004547 | BX950851 | Complete | *Solanum tuberosum* | Scotland; 1985 | 2004 | Shotgun Sequencing | PHRAP |
|  | JG10-08 | NZ_CP007744 | CP007744 | Complete | *Solanum tuberosum* | China; 2010 | 2014 | Illumina Hiseq 2000 | SOAPdenovo v. 2.04 |
|  | 21A | NZ_CP009125 | CP009125 | Complete | *Solanum tuberosum* | Belarus; 1978 | 2014 | Illumina MiSeq | SPAdes v. 3.0 |
|  | 36A | NZ_CP024956 | CP024956 | Complete | *Solanum tuberosum* | Belarus; 1978 | 2017 | Illumina MiSeq | SPAdes v. 3.11 |
| ***P. carotovorum* subsp. *carotovorum*** | PCC21* | NC_018525 | CP003776 | Complete | *Brassica rapa* ssp. *pekinensis* | Korea; 2008 | 2012 | Roche 454 GS FLX | Newbler v. 2.3 |
|  | ATCC 39048 | NZ_QHMC00000000 | QHMC00000000 | Contig (40) | ̶ | USA; 1981 | 2017 | Illumina HiSeq | SPAdes v. MAY-2017 |
|  | ICMP 5702 | NZ_AODT00000000 | AODT00000000 | Contig (46) | *Solanum tuberosum* | Denmark; missing year of collection | 2013 | Illumina | SOAPdenovo v. 1.05 |
|  | NCPPB312 | NZ_JQHJ00000000 | JQHJ00000000 | Contig (55) | *Solanum tuberosum* | Denmark; 1952 | 2014 | Illumina MiSeq | SPAdes v. 3.1.0 |
|  | UGC32 | NZ_AODU01000000 | AODU01000000 | Contig (28) | *Solanum tuberosum* | Peru; missing year of collection | 2013 | Illumina | SOAPdenovo v. 1.05 |
|  | B5 | NZ_JUJS01000000 | JUJS01000000 | Contig (6) | *Brassica rapa* ssp. *pekinensis* | China; 2013 | 2014 | Illumina HiSeq | SOAPdenovo v. 2.01 |
|  | B2 | NZ_JUJR01000000 | JUJR01000000 | Contig (12) | *Brassica rapa* ssp. *pekinensis* | China; 2013 | 2014 | Illumina HiSeq | SOAPdenovo v. 2.01 |
|  | Y57 | NZ_JUJG00000000 | JUJG00000000 | Contig (24) | *Brassica rapa* ssp. *chinensis* | China; 2013 | 2014 | Illumina HiSeq | SOAPdenovo v. 2.01 |
|  | Y39 | NZ_JUJQ00000000 | JUJQ00000000 | Contig (12) | *Brassica rapa* ssp. *chinensis* | China; 2013 | 2014 | Illumina HiSeq | SOAPdenovo v. 2.01 |
| ***P. carotovorum*** | SCC1 | NZ_CP021894 | CP021894 | Complete | *Solanum tuberosum* | Finland; 1982 | 2009 | Roche 454; SOLiD3 | Newbler v. 1.1 |
|  | 3-2 | NZ_CP024842 | CP024842 | Complete | *Solanum tuberosum* | Belarus; 1979 | 2017 | Illumina MiSeq | SPAdes v. 3.5 |
| ***P. carotovorum* subsp. *brasiliense*** | BC1* | NZ_CP009769 | CP009769 | Complete | *Brassica rapa* ssp. *pekinensis* | China; 2002 | 2014 | Illumina | SOAPdenovo v. 2.0 |
|  | BZA12 | NZ_CP024780 | CP024780 | Complete | *Cucumis sativus* | China; 2015 | 2017 | Illumina HiSeq 2500; PacBio RSII | SOAPdenovo v. 2.04; HGAP v. 3.0 |
|  | SX309 | NZ_CP020350 | CP020350 | Complete | *Cucumis sativus* | China; 2015 | 2017 | PacBio | HGAP v. 3.0 |
|  | CFIA 1033 | NZ_JPSO00000000 | JPSO00000000 | Contig (75) | *Solanum tuberosum* | Canada; 2009 | 2014 | Illumina HiSeq | Velvet v. 1.2.10 |
|  | S2 | NZ_JUJF00000000 | JUJF01000000 | Contig (37) | *Brassica rapa* ssp. *pekinensis* | China; 2007 | 2014 | Illumina HiSeq | SOAPdenovo v. 2.01 |
|  | Y60 | NZ_JUJP00000000 | JUJP01000000 | Contig (16) | *Brassica rapa* ssp. *chinensis* | China; 2013 | 2014 | Illumina HiSeq | SOAPdenovo v. 2.01 |
|  | Y65 | NZ_JUJN00000000 | JUJN00000000 | Co ntig (31) | *Brassica rapa* ssp. *chinensis* | China; 2013 | 2014 | Illumina HiSeq | SOAPdenovo v. 2.01 |
| ***P. carotovorum* subsp. *odoriferum*** | BC S7* | ̶ | CP009678 | Complete | *Brassica rapa* ssp. *pekinensis* | China; 2007 | 2014 | PacBio | HGAP v. 2.0 |
|  | S6 | NZ_MTAQ00000000 | MTAQ00000000 | Contig (48) | *Brassica rapa* ssp. *pekinensis* | China; 2010 | 2017 | MPS Illumina | SOAPdenovo v. 2.04 |
|  | T4 | NZ_MTAN00000000 | MTAN00000000 | Contig (78) | *Brassica rapa* ssp. *pekinensis* | China; 2010 | 2017 | MPS Illumina | SOAPdenovo v. 2.04 |
|  | Q47 | NZ_MTAJ00000000 | MTAJ00000000 | Contig (49) | *Apium graveolens* | China; 2014 | 2017 | MPS Illumina | SOAPdenovo v. 2.04 |
|  | NCPPB3839 | NZ_JQOG00000000 | JQOG00000000 | Contig (156) | *Cichorium intybus* | France; 1978 | 2014 | Illumina MiSeq | SPAdes v. 3.1.0 |
|  | NCPPB3841 | NZ_JQOF00000000 | JQOF00000000 | Contig (223) | *Cichorium intybus* | France; 1979 | 2014 | Illumina MiSeq | SPAdes v. 3.1.0 |
|  | Q142 | NZ_MTAO00000000 | MTAO00000000 | Contig (69) | *Apium graveolens* | China; 2014 | 2017 | MPS Illumina | SOAPdenovo v. 2.04 |
| ***P. carotovorum* subsp. *actinidiae*** | KKH3* | NZ_JRMH00000000 | JRMH00000000 | Contig (3) | *Actinidia deliciosa* | South Korea; 2006 | 2014 | Roche 454, Illumina HiSeq, PacBio | gsAssembler 2.6, CLC Genomics v. 6.5.1 |
|  | ICMP 19971 | NZ_MPUI00000000 | MPUI00000000 | Contig (50) | *Actinidia deliciosa* | South Korea; 2016 | 2016 | Illumina HiSeq | SOAPdenovo v. 2.04 |
|  | ICMP 19972 | NZ_MPUJ01000000 | MPUJ01000000 | Contig (49) | *Actinidia deliciosa* | South Korea; 2016 | 2016 | Illumina HiSeq | SOAPdenovo v. 2.04 |
| ***P. aroidearum*** | PC1* | NC_012917 | CP001657 | Complete | *Ornithogalum dubium* | Israel; 2004 | 2009 | Roche 454 GS FLX | Not available |
| ***P. parmentieri*** | SCC3193* | NC_017845 | CP003415 | Complete | *Solanum tuberosum* | Finland; 1980 | 2012 | Roche 454 GS20; SOLiD2 | Newbler v. 2.3 |
|  | WPP 163 | NC_013421 | CP001790 | Complete | *Solanum tuberosum* | USA; 2004 | 2009 | Roche 454 GS FLX | Not available |
|  | RNS08-42-1A | NZ_CP015749 | CP015749 | Complete | *Solanum tuberosum* | France; 2008 | 2016 | PacBio; Illumina HiSeq | HGAP v. 2.0 |
|  | SS90 | NZ_QESW00000000 | QESW00000000 | Contig (50) | *Solanum tuberosum* | Pakistan; 2017 | 2018 | Illumina MiSeq | CLC Genomics v. 10.1.1 |
|  | PB20 | NZ_PDDJ00000000 | PDDJ00000000 | Contig (47) | Sewage water at washing potato station | Russia; 2014 | 2017 | Illumina MiSeq | SPAdes v. 3.6.1 |
|  | CFIA 1002 | NZ_JENG01000000 | JENG01000000 | Contig (42) | *Solanum tuberosum* | Canada; 2007 | 2014 | Illumina HiSeq | ABySS v. MAY-2013 |
| ***P. wasabiae*** | CFBP 3304* | NZ_CP015750 | CP015750 | Complete | *Eutrema wasabi* | Japan; 1987 | 2016 | PacBio | HGAP v. 2.0 |
|  | NCPPB3701 | NZ_JQHP00000000 | JQHP00000000 | Contig (89) | *Eutrema wasabi* | Japan; 1990 | 2014 | Illumina MiSeq | SPAdes v. 3.1.0 |
|  | NCPPB3702 | NZ_JQOH00000000 | JQOH00000000 | Contig (94) | *Eutrema wasabi* | Japan; 1987 | 2014 | Illumina MiSeq | SPAdes v. 3.1.0 |
| ***P. betavasculorum*** | NCPPB2795* | NZ_JQHM00000000 | JQHM00000000 | Contig (93) | *Beta vulgaris* | USA; 1972 | 2014 | Illumina MiSeq | SPAdes v. 3.1.0 |
|  | NCPPB2793 | NZ_JQHL00000000 | JQHL00000000 | Contig (115) | *Beta vulgaris* | USA; 1975 | 2014 | Illumina MiSeq | SPAdes v. 3.1.0 |
| ***P. polaris*** | NIBIO1392* | NZ_CP017482 | CP017482 | Complete | *Solanum tuberosum* | Norway; 2013 | 2016 | PacBio | HGAP v. 3.0 |
|  | NIBIO 1006 | NZ_CP017481 | CP017481 | Complete | *Solanum tuberosum* | Norway; 2010 | 2016 | PacBio | HGAP v. 3.0 |
|  | NCPPB3395 | NZ_JQHN00000000 | JQHN00000000 | Contig (118) | *Solanum tuberosum* | Netherlands; 1985 | 2014 | Illumina MiSeq | SPAdes v. 3.1.0 |
|  | SS28 | NZ_QESX00000000 | QESX00000000 | Contig (37) | *Solanum tuberosum* | Pakistan; 2017 | 2018 | Illumina MiSeq | CLC Genomics v. 10.1.1 |
| ***P. peruviense*** | IFB 5232* | NZ_LXFV00000000 | LXFV00000000 | Contig (60) | *Solanum tuberosum* | Peru; 1979 | 2017 | Roche 454 | Newbler v. 2.6 |
|  | IFB 5229 | NZ_LUBB00000000 | LUBB00000000 | Contig (23) | *Solanum tuberosum* | Peru; 1979 | 2017 | Roche 454 | MIRA v. 1.0.1; SPAdes v. 3.6.2; Velvet v. 1.2.10 |
|  | A350-S18-N16 | NZ_PYUP00000000 | PYUP00000000 | Contig (73) | Fresh water from alpine river | France; 2016 | 2018 | Illumina NextSeq 500 | CLC Genomics v. 9.5.2 |
|  | A97-S13-F16 | NZ_PYUO00000000 | PYUO00000000 | Contig (61) | Fresh water from alpine river | France; 2016 | 2018 | Illumina NextSeq 500 | CLC Genomics v. 9.5.2 |
| ***Candidatus* Pectobacterium maceratum** | PB69* | NZ_PDVY00000000 | PDVY00000000 | Contig (21) | *Solanum tuberosum* | Russia; 2012 | 2016 | Illumina MiSeq | SPAdes v. 3.8.0 |
|  | PB70 | NZ_PDVZ00000000 | PDVZ00000000 | Contig (28) | *Solanum tuberosum* | Russia; 2012 | 2016 | Illumina MiSeq | SPAdes v. 3.8.0 |
|  | F018 | NZ_PDVV00000000 | PDVV00000000 | Contig (27) | *Brassica oleracea* | Russia; 1947 | 2016 | Illumina MiSeq | SPAdes v. 3.8.0 |
|  | F131 | NZ_PDVW00000000 | PDVW00000000 | Contig (87) | *Solanum tuberosum* | Russia; 1993 | 2016 | Illumina MiSeq | SPAdes v. 3.8.0 |
|  | F135 | NZ_PDVX00000000 | PDVX00000000 | Contig (75) | *Solanum tuberosum* | Russia; 2012 | 2016 | Illumina MiSeq | SPAdes v. 3.8.0 |

Phrap, phragment assembly program; SOAPdenovo, Short Oligonucleotide Alignment Program de novo; ABySS, Assembly By Short Sequences; SPAdes, SPAdes, St. Petersburg genome assembler, HGAP, Hierarchical Genome Assembly Process; Newbler, GS De Novo Assembler; MIRA, Mimicking Intelligent Read Assembly.

For those incomplete genomes it is provided the number of contigs within a parenthesis.

**^*^**Representative strains that were selected per species for carrying out all the comparative genomics analysis conducted throughout this study.
