# Supplementary material for: Genome-Wide Analyses Revealed Remarkable Heterogeneity in Pathogenicity Determinants, Antimicrobial Compounds, and CRISPR-Cas Systems of Complex Phytopathogenic Genus Pectobacterium
Source: Pathogens. 2019 Nov 20;8(4):247. doi: 10.3390/pathogens8040247 (PMC6963963; doi:10.3390/pathogens8040247)
Supplement: Supplementary file 1 [file pathogens-08-00247-s001.zip › pathogens-633436supplementary/Supplementary Files/TABLE S40.docx]

**Table S40.** Detail description of main features of the Clustered Regularly Interspaced Short Palindromic Repeats (CRISPR) arrays across the twelve species within *Pectobacterium* genus.

| **Species name** | **Strain** | **Clustered Regularly Interspaced Short Palindromic Repeats (CRISPR) arrays features** | | | | | | | | | |
| --- | --- | --- | --- | --- | --- | --- | --- | --- | --- | --- | --- |
|  |  | **CRISPR-Cas Type** | **RefSeq-Accession Number ᵃ** | **No. of CRISPRs** | **Position** | | **Strand** | **Size (bp)** | **Repeats Length (bp)** | **Sp.** | **Direct Repeat Consensus** |
|  |  |  |  |  | Start | End |  |  |  |  |  |
| ***Pectobacterium atrosepticum*** | SCRI1043 | Cas-Type IF | NC_004547 | *CRISPR-1* | 4124152 | 4125860 | - | 1709 | 28 | 28 | TTTCTAAGCTGCCTATACGGCAGTGAAC |
|  |  |  |  | *CRISPR-2* | 4135961 | 4136589 | + | 629 | 28 | 10 | GTTCACTGCCGTACAGGCAGCTTAGAAA |
|  |  |  |  | *CRISPR-3* | 4134776 | 4134984 | + | 209 | 28 | 3 | GTTCACTGCCGTACAGGCAGCTTAGAAA |
|  | JG10-08 | Cas-Type IF | NZ_CP007744 | *CRISPR-1* | 4078168 | 4078917 | - | 750 | 28 | 12 | TTTCTAAGCTGCCTATACGGCAGTGAAC |
|  |  |  |  | *CRISPR-2* | 4089018 | 4089586 | + | 569 | 28 | 9 | GTTCACTGCCGTACAGGCAGCTTAGAAA |
|  |  |  |  | *CRISPR-3* | 4087833 | 4088041 | + | 209 | 28 | 3 | GTTCACTGCCGTACAGGCAGCTTAGAAA |
|  | 21A | Cas-Type IF | NZ_CP009125 | *CRISPR-1* | 4032570 | 4033319 | - | 750 | 28 | 12 | TTTCTAAGCTGCCTATACGGCAGTGAAC |
|  |  |  |  | *CRISPR-2* | 4042235 | 4042443 | + | 569 | 28 | 9 | GTTCACTGCCGTACAGGCAGCTTAGAAA |
|  |  |  |  | *CRISPR-3* | 4043420 | 4043988 | + | 209 | 28 | 3 | GTTCACTGCCGTACAGGCAGCTTAGAAA |
|  | 36A | Cas-Type IF | NZ_CP024956 | *CRISPR-1* | 4065842 | 4068030 | - | 2189 | 28 | 36 | TTTCTAAGCTGCCTGTACGGCAGTGAAC |
|  |  |  |  | *CRISPR-2* | 4078072 | 4078579 | + | 508 | 28 | 8 | GTTCACTGCCGTATAGGCAGCTTAGAAA |
| ***Pectobacterium carotovorum* subsp. *carotovorum*** | PCC21 | Cas-Type IE | NC_018525 | *CRISPR-1* | 419087 | 420938 | + | 1851 | 29 | 30 | GTGTTCCCCGCGCCAGCGGGGATAAACCG |
|  |  | Cas-Type IF |  | *CRISPR-2* | 3972661 | 3974968 | - | 2308 | 28 | 38 | TTTCTAAGCTGCCTGTACGGCAGTGAAC |
|  |  |  |  | *CRISPR-3* | 3983542 | 3983750 | + | 209 | 28 | 3 | GTTCACTGCCGCATAGGCAGCTTAGAAA |
|  | BC T5 | Cas-Type IE | NZ_JUJS01000002 | *CRISPR-1* | 155372 | 156986 | + | 1614 | 29 | 26 | GTGTTCCCCGCGCCAGCGGGGATAAACCG |
|  |  |  |  | *CRISPR-2* | 159748 | 160250 | + | 502 | 29 | 7 | GTGTTCCCCGCGCCAGCGGGGATAAACCG |
|  |  | Cas-Type IF | NZ_JUJS01000003 | *CRISPR-3* | 165876 | 166384 | + | 509 | 28 | 8 | GTTCACTGCCGCATAGGCAGCTTAGAAA |
|  |  |  |  | *CRISPR-4* | 172806 | 174333 | - | 1528 | 28 | 25 | TTTCTAAGCTGCCTGTACGGCAGTGAAC |
|  |  |  |  | *CRISPR-5* | 183309 | 183697 | + | 389 | 28 | 6 | GTTCACTGCCGTATAGGCAGCTTAGAAA |
|  | BC T2 | Cas-Type IE | NZ_JUJR01000007 | *CRISPR-1* | 46795 | 47861 | + | 1066 | 29 | 17 | GTGTTCCCCGCGCCAGCGGGGATAAACCG |
|  |  |  |  | *CRISPR-2* | 48661 | 49485 | + | 824 | 29 | 13 | GTGTTCCCCGCGCCAGCGGGGATAAACCG |
|  |  | Orphan CRISPR | NZ_JUJR01000003 | *CRISPR-3* | 168890 | 169218 | + | 329 | 28 | 5 | GTTCACTGCCGTGTAGGCAGCTTAGAAA |
| ***Pectobacterium carotovorum* subsp. *brasiliense*** | BC1 | Cas-Type IF | NZ_CP009769 | *CRISPR-1* | 4011683 | 4012251 | + | 1705 | 28 | 9 | GTTCACTGCCGTATAGGCAGCTTAGAAA |
|  |  |  |  | *CRISPR-2* | 4018179 | 4020113 | - | 1934 | 28 | 31 | TTTCTAAGCTGCCTATACGGCAGTGAAC |
|  |  |  |  | *CRISPR-3* | 4028750 | 4029197 | + | 448 | 28 | 7 | GTTCACTGCCGTATAGGCAGCTTAGAAA |
|  | BZA12 | Cas-Type IE | NZ_CP024780 | *CRISPR-1* | 2853179 | 2855589 | - | 2411 | 29 | 39 | CGGTTTATCCCCGCTGGCGCGGGGAACAC |
|  |  | Cas-Type IF |  | *CRISPR-2* | 4210799 | 4212450 | + | 1652 | 28 | 27 | GTTCACTGCCGGATAGGCAGCTTAGAAA |
|  |  |  |  | *CRISPR-3* | 4218519 | 4219448 | - | 930 | 28 | 15 | TTTCTAAGCTGCCTATGCGGCAGTGAAC |
|  | SX309 | Cas-Type IF | NZ_CP020350 | *CRISPR-1* | 869781 | 870288 | - | 508 | 28 | 8 | TTTCTAAGCTGCCTATGCGGCAGTGAAC |
|  |  |  |  | *CRISPR-2* | 914829 | 915397 | + | 569 | 28 | 9 | GTTCACTGCCGTATAGGCAGCTTAGAAA |
|  |  |  |  | *CRISPR-3* | 921324 | 921892 | - | 569 | 28 | 9 | TTTCTAAGCTGCCTATACGGCAGTGAAC |
|  |  | Cas-Type IE |  | *CRISPR-4* | 4456981 | 4459145 | - | 2165 | 29 | 35 | CGGTTTATCCCCGCTGGCGCGGGGAACAC |
| ***Pectobacterium carotovorum* subsp. *odoriferum*** | BC S7 | Cas-Type IE | CP009678 | *CRISPR-1* | 414248 | 416166 | + | 1917 | 29 | 31 | GTGTTCCCCGCGCCAGCGGGGATAAACCG |
|  |  | Orphan CRISPRs |  | *CRISPR-2* | 3970131 | 3970698 | + | 568 | 28 | 9 | GTTCACTGCCGTATAGGCAGCTTAGAAA |
|  |  |  |  | *CRISPR-3* | 3977565 | 3978134 | - | 569 | 28 | 9 | TTTCTAAGCTGCCTATACGGCAGTGAAC |
|  | S6 | Cas-Type IE | NZ_MTAQ01000002 | *CRISPR-1* | 159215 | 161135 | + | 1921 | 29 | 31 | GTGTTCCCCGCGCCAGCGGGGATAAACCG |
|  |  | Orphan CRISPRs | NZ_MTAQ01000011 | *CRISPR-2* | 18864 | 19433 | + | 569 | 28 | 9 | GTTCACTGCCGTATAGGCAGCTTAGAAA |
|  |  |  |  | *CRISPR-3* | 26300 | 26867 | - | 568 | 28 | 9 | TTTCTAAGCTGCCTATACGGCAGTGAAC |
|  | T4 | Cas-Type IF | NZ_MTAN01000001 | *CRISPR-1* | 247376 | 247703 | - | 328 | 28 | 5 | TTTCTAAGCTGCCTATACGGCAGTGAAC |
|  |  |  |  | *CRISPR-2* | 256281 | 257328 | + | 1048 | 28 | 17 | GTTCACTGCCGTATAGGCAGCTTAGAAA |
|  |  |  |  | *CRISPR-3* | 265732 | 266719 | + | 987 | 28 | 16 | GTTCACTGCCGTATAGGCAGCTTAGAAA |
| ***Pectobacterium carotovorum* subsp. *actinidiae*** | KKH3 | Cas-Type IF | NZ_JRMH01000001 | *CRISPR-1* | 3739138 | 3741268 | - | 2131 | 28 | 35 | TTTCTAAGCTGCCTATACGGCAGTGAAC |
|  |  |  |  | *CRISPR-2* | 3749841 | 3751369 | + | 1528 | 28 | 25 | GTTCACTGCCGCATAGGCAGCTTAGAAA |
|  | ICMP 19971 | Cas-Type IF | NZ_MPUI01000002 | *CRISPR-1* | 317229 | 318757 | - | 1528 | 28 | 25 | TTTCTAAGCTGCCTATGCGGCAGTGAAC |
|  |  |  |  | *CRISPR-2* | 327330 | 329460 | + | 2131 | 28 | 35 | GTTCACTGCCGTATAGGCAGCTTAGAAA |
|  | ICMP 19972 | Cas-Type IF | NZ_MPUJ01000005 | *CRISPR-1* | 176733 | 178863 | - | 2131 | 28 | 35 | TTTCTAAGCTGCCTATACGGCAGTGAAC |
|  |  |  |  | *CRISPR-2* | 187436 | 188964 | + | 1528 | 28 | 25 | GTTCACTGCCGCATAGGCAGCTTAGAAA |
| ***Pectobacterium aroidearum*** | PC1 | Cas-Type IIIA | NC_012917 | *CRISPR-1* | 3681990 | 3682376 | + | 386 | 37 | 5 | GTCCTTACGGACGCTCCCTGACTGAAGGGATTAAGAC |
| ***Pectobacterium parmentieri*** | SCC3193 | Cas-Type IE | NC_017845 | *CRISPR-1* | 451274 | 452587 | + | 1314 | 29 | 21 | GTGTTCCCCGCGCCAGCGGGGATAAACCG |
|  |  |  |  | *CRISPR-2* | 454456 | 455277 | + | 821 | 29 | 13 | GTGTTCCCCGCGCCAGCGGGGATAAACCG |
|  |  | Cas-Type IF |  | *CRISPR-3* | 4068747 | 4069614 | + | 868 | 28 | 14 | GTTCACTGCCGTATAGGCAGCTTAGAAA |
|  |  |  |  | *CRISPR-4* | 4087266 | 4089154 | + | 1889 | 28 | 31 | GTTCACTGCCGTATAGGCAGCTTAGAAA |
|  | WPP163 | Cas-Type IE | NC_013421 | *CRISPR-1* | 436573 | 437578 | + | 1006 | 29 | 16 | GTGTTCCCCGCGCCAGCGGGGATAAACCG |
|  |  |  |  | *CRISPR-2* | 439447 | 439841 | + | 394 | 29 | 6 | GTGTTCCCCGCGCCAGCGGGGATAAACCG |
|  |  | Cas-Type IF |  | *CRISPR-3* | 4009625 | 4010672 | + | 1048 | 28 | 17 | GTTCACTGCCGTATAGGCAGCTTAGAAA |
|  |  |  |  | *CRISPR-4* | 4027943 | 4029473 | + | 1532 | 28 | 25 | GTTCACTGCCGTATAGGCAGCTTAGAAA |
|  | RNS 08-42-1A | Cas-Type IF | NZ_CP015749 | *CRISPR-1* | 85425 | 87018 | - | 1594 | 28 | 26 | TTTCTAAGCTGCCTATACGGCAGTGAAC |
|  |  |  |  | *CRISPR-2* | 104235 | 106296 | - | 2062 | 28 | 34 | TTTCTAAGCTGCCTATACGGCAGTGAAC |
|  |  | Cas-Type IE |  | *CRISPR-3* | 3624488 | 3625309 | - | 821 | 29 | 13 | CGGTTTATCCCCGCTGGCGCGGGGAACAC |
|  |  |  |  | *CRISPR-4* | 3627179 | 3628306 | - | 1128 | 29 | 18 | CGGTTTATCCCCGCTGGCGCGGGGAACAC |
| ***Pectobacterium wasabiae*** | CFBP 3304 | Cas-Type IF | NZ_CP015750 | *CRISPR-1* | 737392 | 737599 | + | 208 | 28 | 3 | GTTCACTGCCGCATAGGCAGCTTAGAAA |
|  |  |  |  | *CRISPR-2* | 753523 | 754632 | + | 1110 | 28 | 18 | GTTCACTGCCGTACAGGCAGCTTAGAAA |
|  |  | Cas-Type IE |  | *CRISPR-3* | 2215995 | 2216694 | + | 700 | 29 | 11 | GTGTTCCCCGCGCCAGCGGGGATAAACCG |
|  |  |  |  | *CRISPR-4* | 2218689 | 2219267 | + | 579 | 29 | 9 | GTGTTCCCCGCGCCAGCGGGGATAAACCG |
|  | NCPPB 3701 | Cas-Type IF | NZ_JQHP01000005 | *CRISPR-1* | 167732 | 167939 | + | 208 | 28 | 3 | GTTCACTGCCGCATAGGCAGCTTAGAAA |
|  |  |  |  | *CRISPR-2* | 183864 | 184852 | + | 989 | 28 | 16 | GTTCACTGCCGTACAGGCAGCTTAGAAA |
|  |  | Cas-Type IE | NZ_JQHP01000016 | *CRISPR-3* | 34220 | 34919 | + | 700 | 29 | 11 | GTGTTCCCCGCGCCAGCGGGGATAAACCG |
|  |  |  |  | *CRISPR-4* | 36914 | 37492 | + | 579 | 29 | 9 | GTGTTCCCCGCGCCAGCGGGGATAAACCG |
|  | NCPPB 3702 | Cas-Type IF | NZ_JQOH01000005 | *CRISPR-1* | 167732 | 167939 | + | 208 | 28 | 3 | GTTCACTGCCGCATAGGCAGCTTAGAAA |
|  |  |  |  | *CRISPR-2* | 183864 | 184852 | + | 989 | 28 | 16 | GTTCACTGCCGTACAGGCAGCTTAGAAA |
|  |  | Cas-Type IE | NZ_JQOH01000014 | *CRISPR-3* | 34220 | 34919 | + | 700 | 29 | 11 | GTGTTCCCCGCGCCAGCGGGGATAAACCG |
|  |  |  |  | *CRISPR-4* | 36914 | 37492 | + | 579 | 29 | 9 | GTGTTCCCCGCGCCAGCGGGGATAAACCG |
| ***Pectobacterium betavasculorum*** | NCPPB 2795 | Cas-Type IF | NZ_JQHM01000003 | *CRISPR-1* | 296886 | 297334 | - | 450 | 28 | 7 | TTTCTAAGCTGCCTATGCGGCAGTGAAC |
|  |  |  |  | *CRISPR-2* | 307425 | 308832 | + | 1408 | 28 | 23 | GTTCACTGCCGCACAGGCAGCTTAGAAA |
|  |  |  |  | *CRISPR-3* | 316816 | 317443 | - | 628 | 28 | 10 | TTTCTAAGCTGCCTATGCGGCAGTGAAC |
|  | NCPPB 2793 | Cas-Type IF | NZ_JQHL01000006 | *CRISPR-1* | 268498 | 270747 | - | 2250 | 28 | 37 | TTTCTAAGCTGCCTATGCGGCAGTGAAC |
|  |  |  |  | *CRISPR-2* | 280780 | 281289 | + | 510 | 28 | 8 | GTTCACTGCCGTACAGGCAGCTTAGAAA |
|  |  | Orphan CRISPRs | NZ_JQHL01000008 | *CRISPR-3* | 5526 | 6333 | - | 807 | 28 | 13 | TTTCTAAGCTGCCTATGCGGCAGTGAAC |
|  |  |  | NZ_JQHL01000044 | *CRISPR-4* | 2484 | 2991 | - | 508 | 28 | 8 | TTTCTAAGCTGCCTATGCGGCAGTGAAC |
|  |  |  | NZ_JQHL01000079 | *CRISPR-5* | 5 | 453 | - | 448 | 28 | 7 | TTTCTAAGCTGCCTATGCGGCAGTGAAC |
|  |  |  | NZ_JQHL01000085 | *CRISPR-6* | 36 | 363 | + | 328 | 28 | 5 | GTTCACTGCCGTACAGGCAGCTTAGAAA |
| ***Pectobacterium polaris*** | NIBIO1392 | Cas-Type IF | NZ_CP017482 | *CRISPR-1* | 2836771 | 2837338 | + | 568 | 28 | 9 | GTTCACTGCCGTATAGGCAGCTTAGAAA |
|  |  |  |  | *CRISPR-2* | 2843237 | 2844824 | - | 1588 | 28 | 26 | TTTCTAAGCTGCCTATACGGCAGTGAAC |
|  |  |  |  | *CRISPR-3* | 2853405 | 2853733 | + | 329 | 28 | 5 | GTTCACTGCCGCATAGGCAGCTTAGAAA |
|  |  |  |  | *CRISPR-4* | 2854723 | 2855532 | + | 810 | 28 | 13 | TTTCACTGCCGTATAGGCAGCTTAGAAA |
|  |  | Cas-Type IE |  | *CRISPR-5* | 4225587 | 4227397 | + | 1811 | 29 | 29 | GTGTTCCCCGCGCCAGCGGGGATAAACCG |
|  |  |  |  | *CRISPR-6* | 4229609 | 4231676 | + | 2069 | 29 | 32 | GAGTTCCCCGCGCCAGCGGGGATAAACCG |
|  | NCPPB 3395 | Cas-Type IE | NZ_JQHN01000002 | *CRISPR-1* | 228234 | 228872 | - | 639 | 29 | 10 | CGGTTTATCCCCGCTGGCGCGGGGAACAC |
|  |  | Orphan CRISPR | NZ_JQHN01000005 | *CRISPR-2* | 171621 | 171888 | - | 268 | 28 | 4 | TTTCTAAGCTGCCTATACGGCAGTGAAC |
|  | SS28 | Cas-Type IE | NZ_QESX01000001 | *CRISPR-1* | 152598 | 153602 | + | 1005 | 29 | 16 | GTGTTCCCCGCGCCAGCGGGGATAAACCG |
|  |  |  |  | *CRISPR-2* | 155792 | 157040 | + | 1249 | 29 | 20 | GTGTTCCCCGCGCCAGCGGGGATAAACCG |
|  |  | Orphan CRISPR | NZ_QESX01000005 | *CRISPR-3* | 326415 | 326623 | - | 209 | 28 | 3 | TTTCTAAGCTGCCTATACGGCAGTGAAC |
| ***Pectobacterium peruviense*** | IFB5232 | Cas-Type IF | NZ_LXFV01000032 | *CRISPR-1* | 45748 | 46377 | - | 629 | 30 | 10 | TTTCTAAGCTGCCTATACGGCAGTGAACGT |
|  |  |  |  | *CRISPR-2* | 56063 | 56870 | + | 808 | 28 | 13 | GTTCACTGCCGCACAGGCAGCTTAGAAA |
|  | A350-S18-N16 | Cas-Type IF | NZ_PYUP01000024 | *CRISPR-1* | 218359 | 218688 | - | 329 | 28 | 5 | TTTCTAAGCTGCCTATACGGCAGTGAAC |
|  |  |  |  | *CRISPR-2* | 228380 | 229967 | + | 1588 | 28 | 26 | GTTCACTGCCGTATAGGCAGCTTAGAAA |
|  |  |  |  | *CRISPR-3* | 235966 | 236833 | - | 868 | 28 | 14 | TTTCTAAGCTGCCTATACGGCAGTGAAC |
|  | A97-S13-F16 | Cas-Type IF | NZ_PYUO01000010 | *CRISPR-1* | 9718 | 10106 | + | 389 | 28 | 6 | GTTCACTGCCGTACAGGCAGCTTAGAAA |
| ***Candidatus* Pectobacterium maceratum** | PB69 | Cas-Type IF | NZ_PDVY01000003 | *CRISPR-1* | 170472 | 172721 | - | 2250 | 28 | 37 | TTTCTAAGCTGCCTATACGGCAGTGAAC |
|  |  |  |  | *CRISPR-2* | 181673 | 182240 | + | 568 | 28 | 9 | GTTCACTGCCGTATAGGCAGCTTAGAAA |
|  |  | Cas-Type IE | NZ_PDVY01000002 | *CRISPR-3* | 839277 | 840525 | - | 1249 | 29 | 20 | CGGTTTATCCCCGCTAGCGCGGGGAACAC |
|  |  |  |  | *CRISPR-4* | 842722 | 844397 | - | 1676 | 29 | 27 | CGGTTTATCCCCGCTGGCGCGGGGAACAC |
|  | PB70 | Cas-Type IF | NZ_PDVZ01000004 | *CRISPR-1* | 170472 | 172721 | - | 2250 | 28 | 37 | TTTCTAAGCTGCCTATACGGCAGTGAAC |
|  |  |  |  | *CRISPR-2* | 181673 | 182240 | + | 568 | 28 | 9 | GTTCACTGCCGTATAGGCAGCTTAGAAA |
|  |  | Cas-Type IE | NZ_PDVZ01000002 | *CRISPR-3* | 462372 | 463620 | - | 1249 | 29 | 20 | CGGTTTATCCCCGCTAGCGCGGGGAACAC |
|  |  |  |  | *CRISPR-4* | 465817 | 467492 | - | 1676 | 29 | 27 | CGGTTTATCCCCGCTGGCGCGGGGAACAC |
|  | F135 | Cas-Type IF | NZ_PDVX01000002 | *CRISPR-1* | 281599 | 282166 | - | 568 | 28 | 9 | TTTCTAAGCTGCCTATGCGGCAGTGAAC |
|  |  | Orphan CRISPRs | NZ_PDVX01000056 | *CRISPR-2* | 1649 | 4136 | - | 2488 | 28 | 41 | TTTCTAAGCTGCCTGTACGGCAGTGAAC |
|  |  |  | NZ_PDVX01000069 | *CRISPR-3* | 100 | 848 | + | 749 | 28 | 12 | GTTCACTGCCGTACAGGCAGCTTAGAAA |

Sp., Spacers count

ᵃRefSeq accession number is provided for each species with complete genome as well as for the contigs of draft genomes where the CRISPRs were predicted
