# Supplementary figures and images for: Genome-Wide Analyses Revealed Remarkable Heterogeneity in Pathogenicity Determinants, Antimicrobial Compounds, and CRISPR-Cas Systems of Complex Phytopathogenic Genus Pectobacterium
Source: Pathogens. 2019 Nov 20;8(4):247. doi: 10.3390/pathogens8040247 (PMC6963963; doi:10.3390/pathogens8040247)

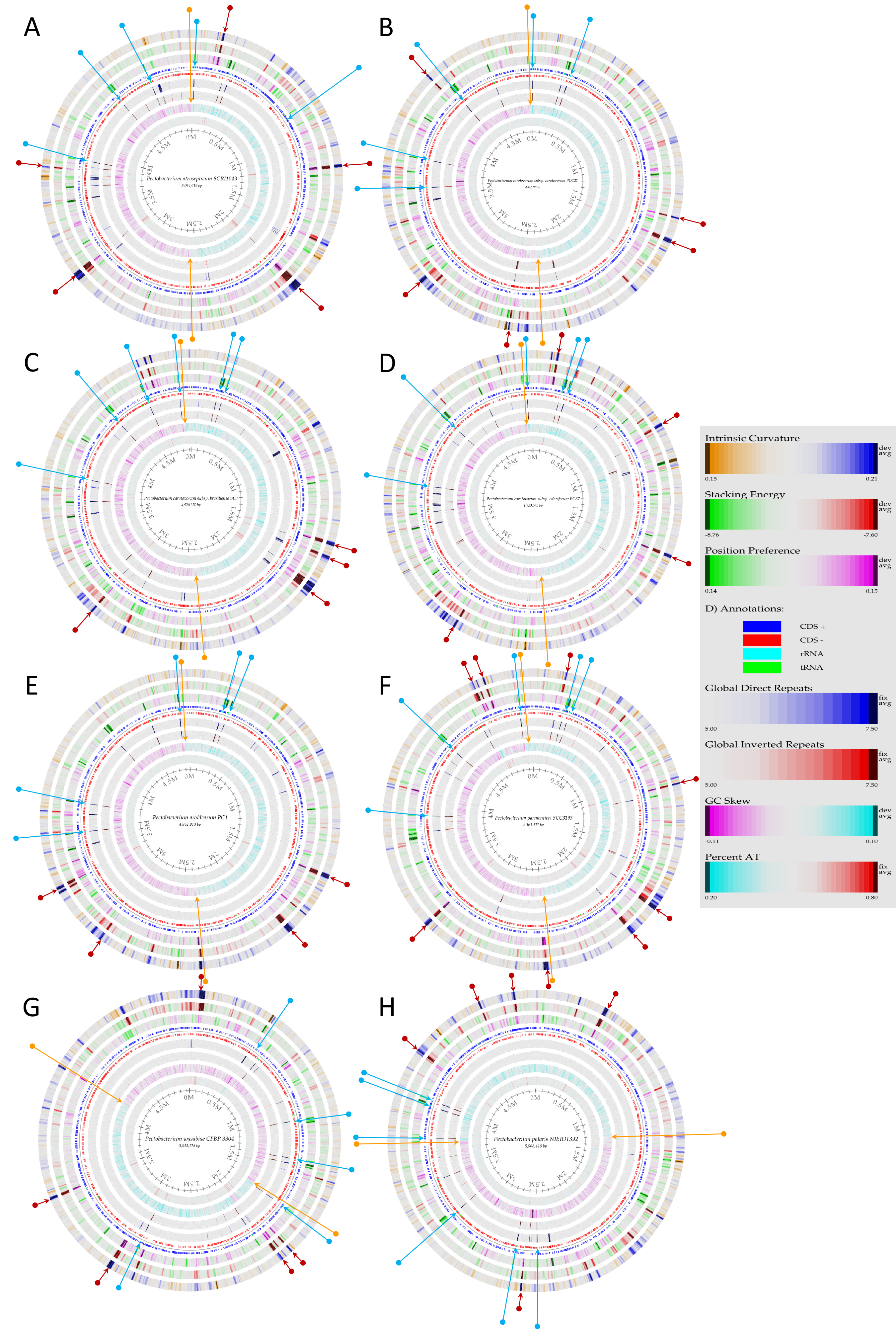

Supplement: Supplementary file 1 [file pathogens-08-00247-s001.zip › pathogens-633436supplementary/Supplementary Files/Figure S1.tif]

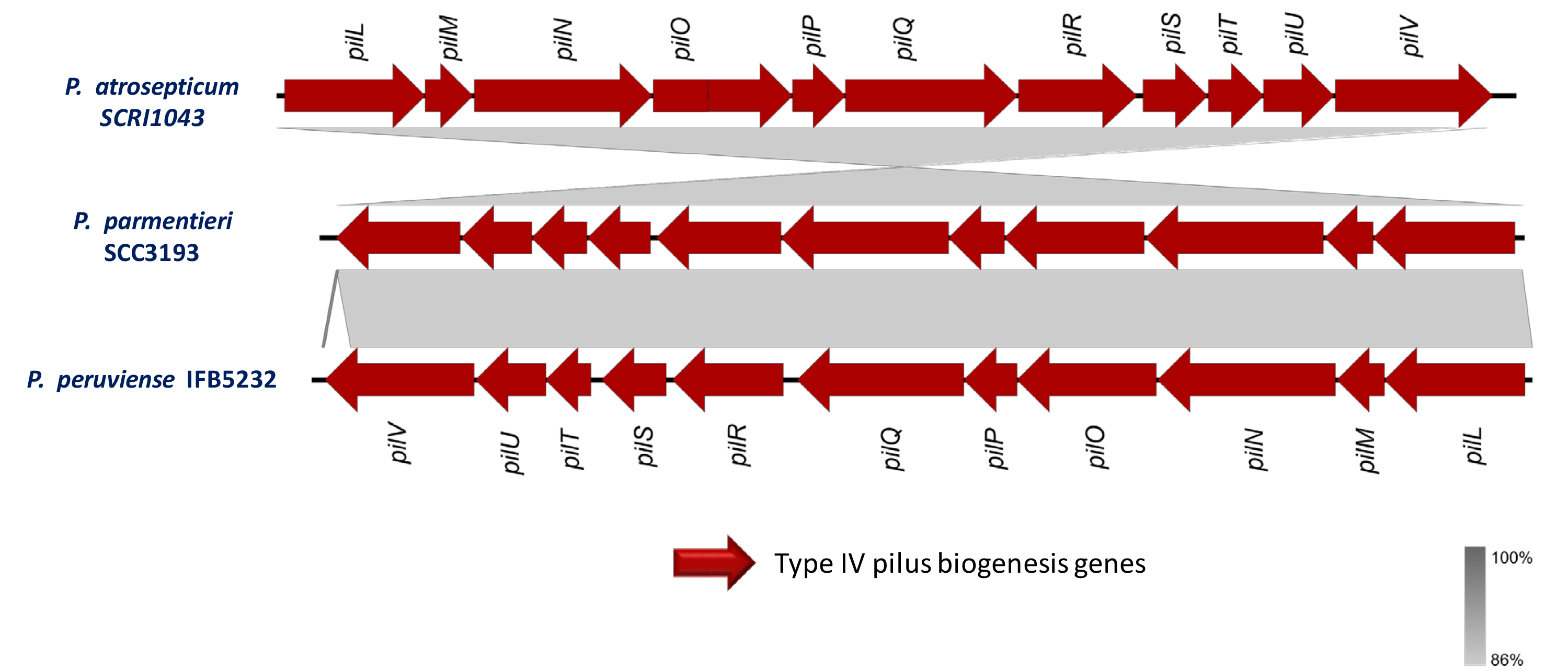

Supplement: Supplementary file 1 [file pathogens-08-00247-s001.zip › pathogens-633436supplementary/Supplementary Files/Figure S10.tif]

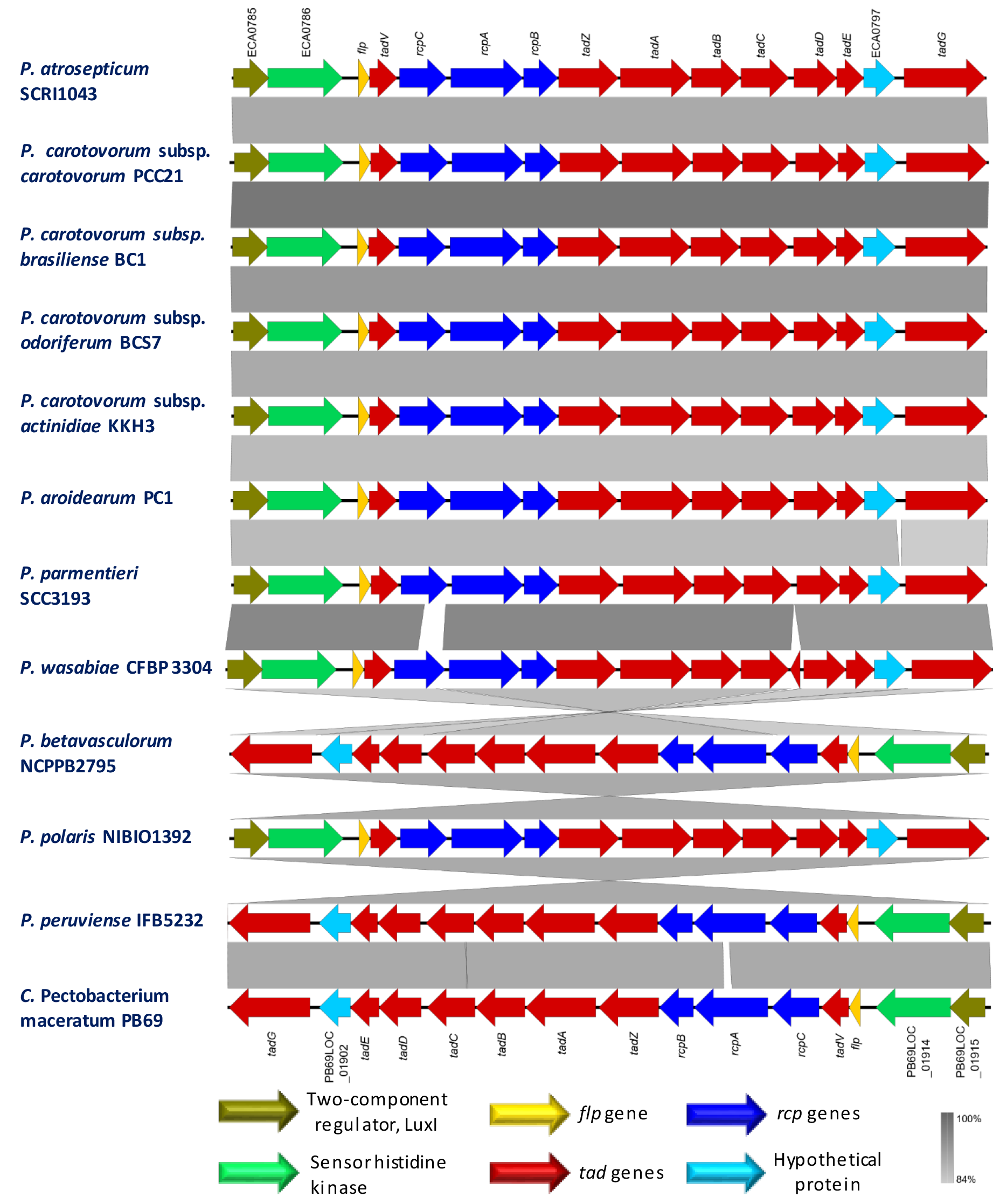

Supplement: Supplementary file 1 [file pathogens-08-00247-s001.zip › pathogens-633436supplementary/Supplementary Files/Figure S11.tif]

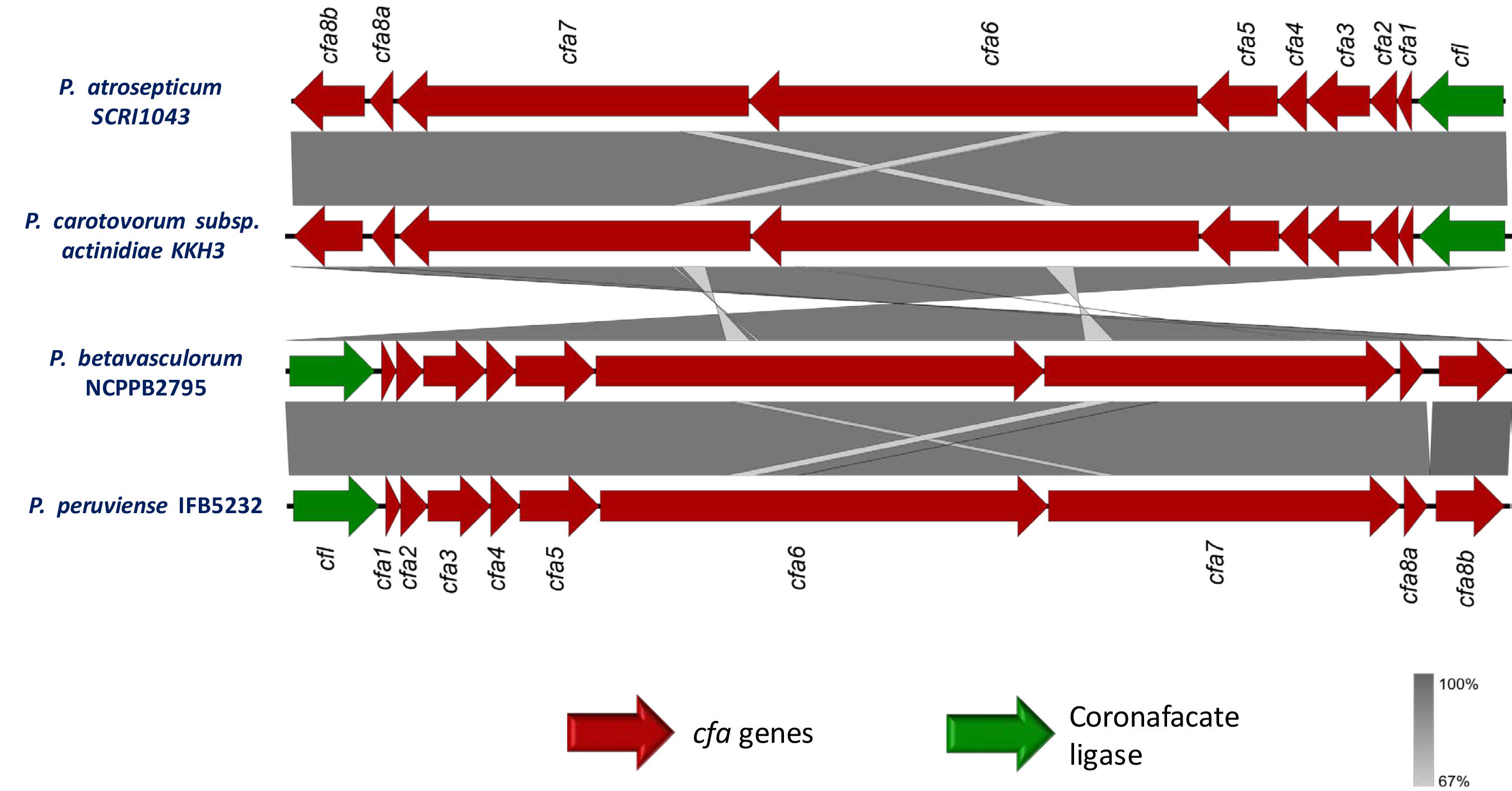

Supplement: Supplementary file 1 [file pathogens-08-00247-s001.zip › pathogens-633436supplementary/Supplementary Files/Figure S12.tif]

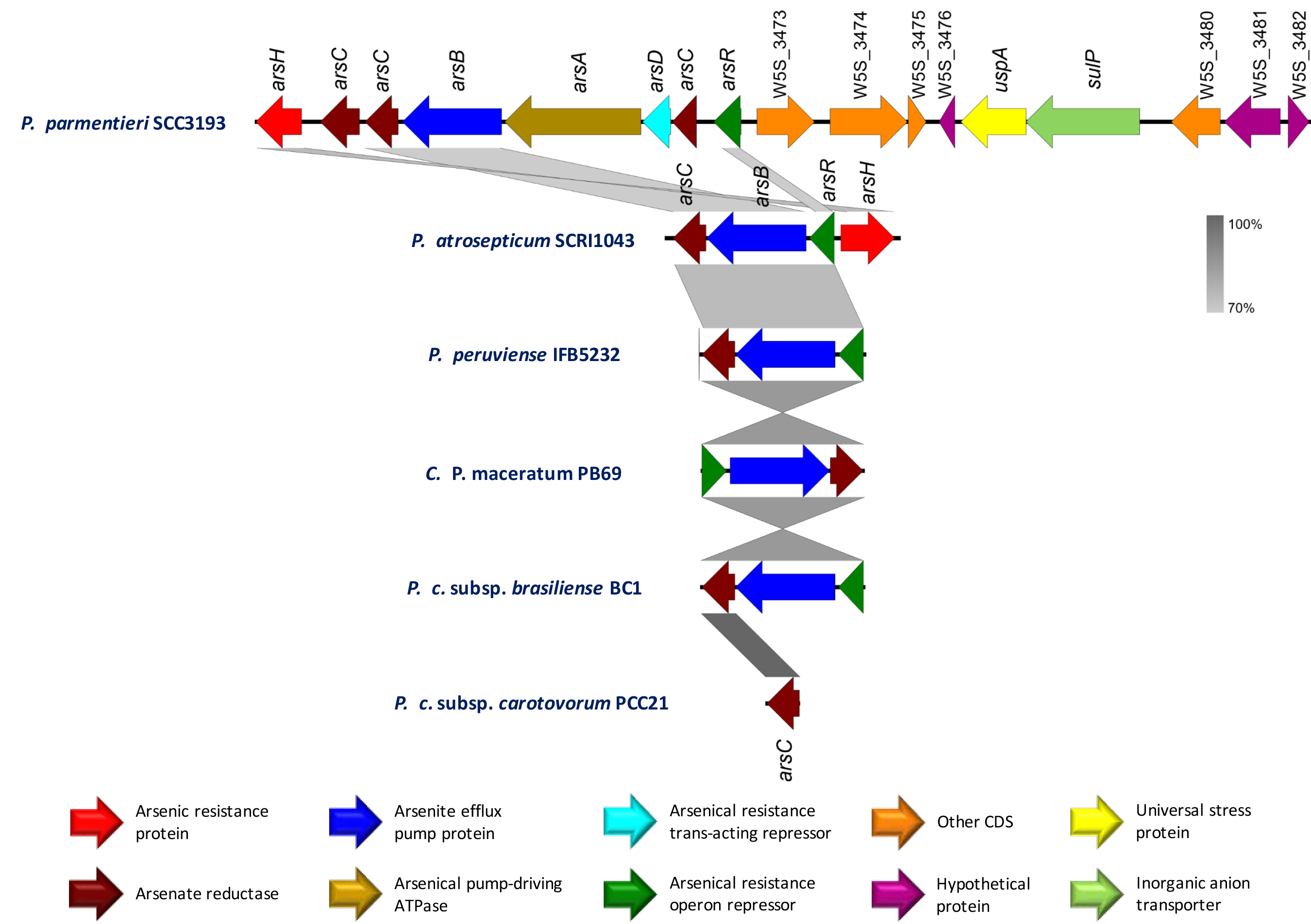

Supplement: Supplementary file 1 [file pathogens-08-00247-s001.zip › pathogens-633436supplementary/Supplementary Files/Figure S13.tif]

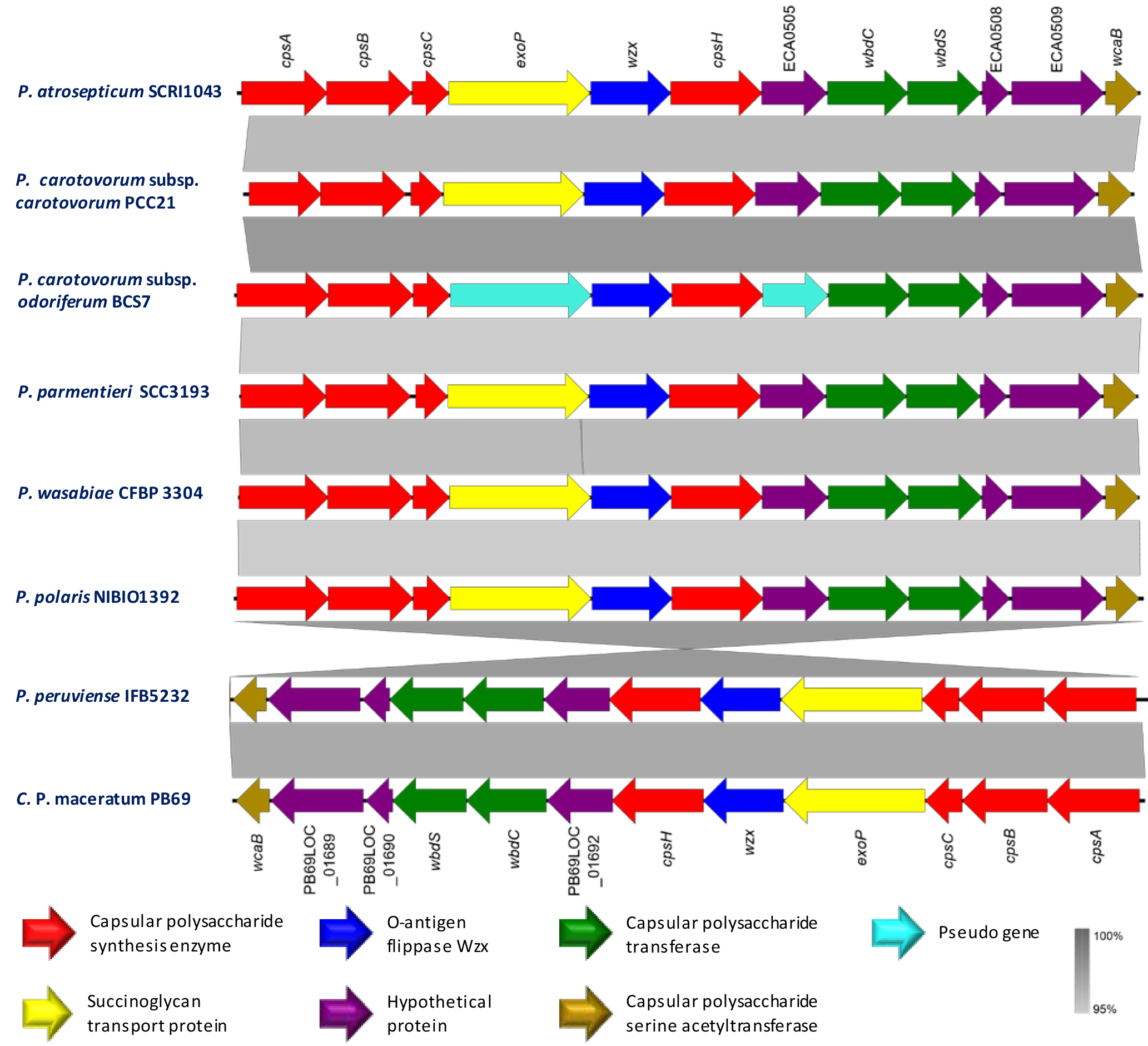

Supplement: Supplementary file 1 [file pathogens-08-00247-s001.zip › pathogens-633436supplementary/Supplementary Files/Figure S14.tif]

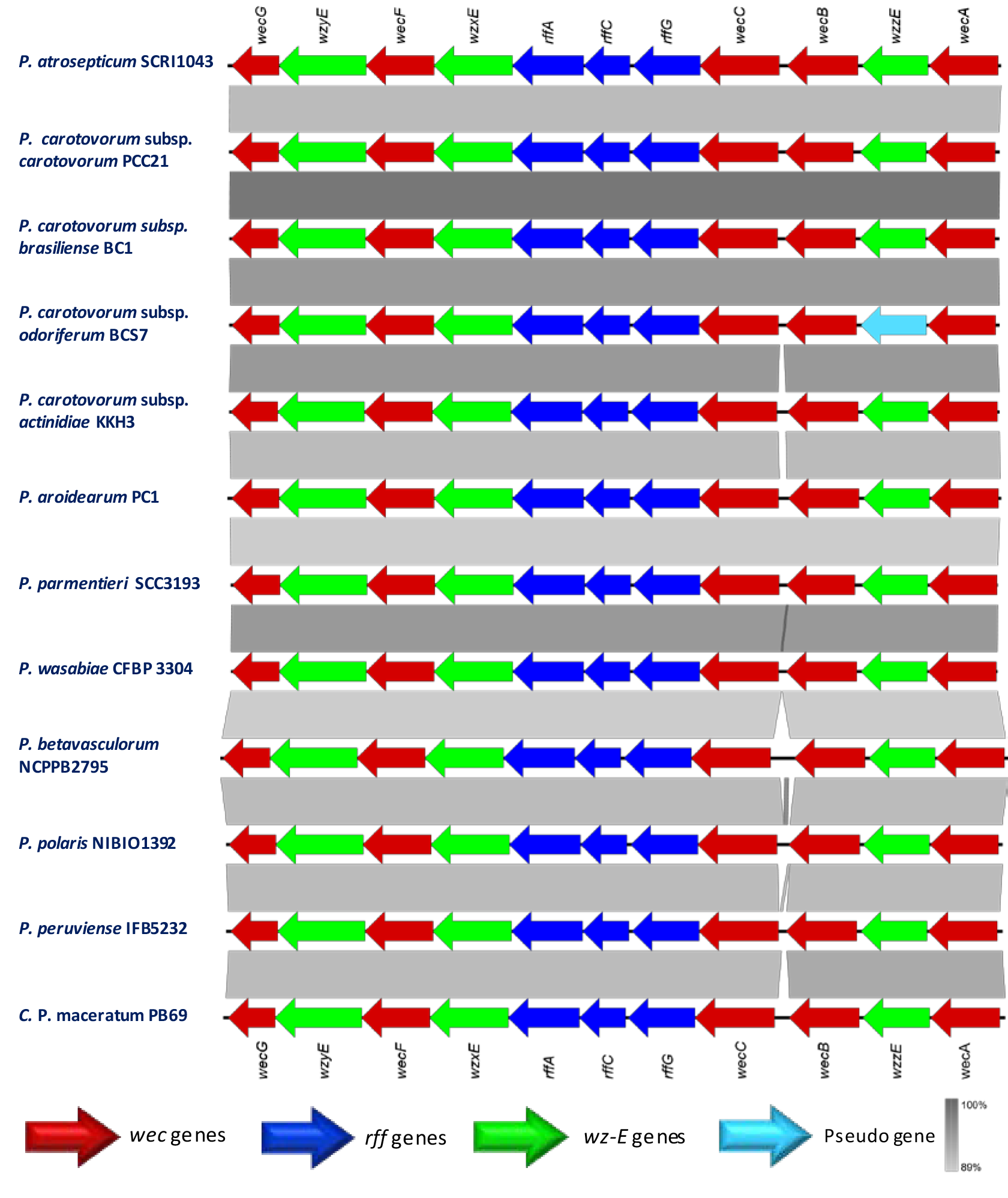

Supplement: Supplementary file 1 [file pathogens-08-00247-s001.zip › pathogens-633436supplementary/Supplementary Files/Figure S15.tif]

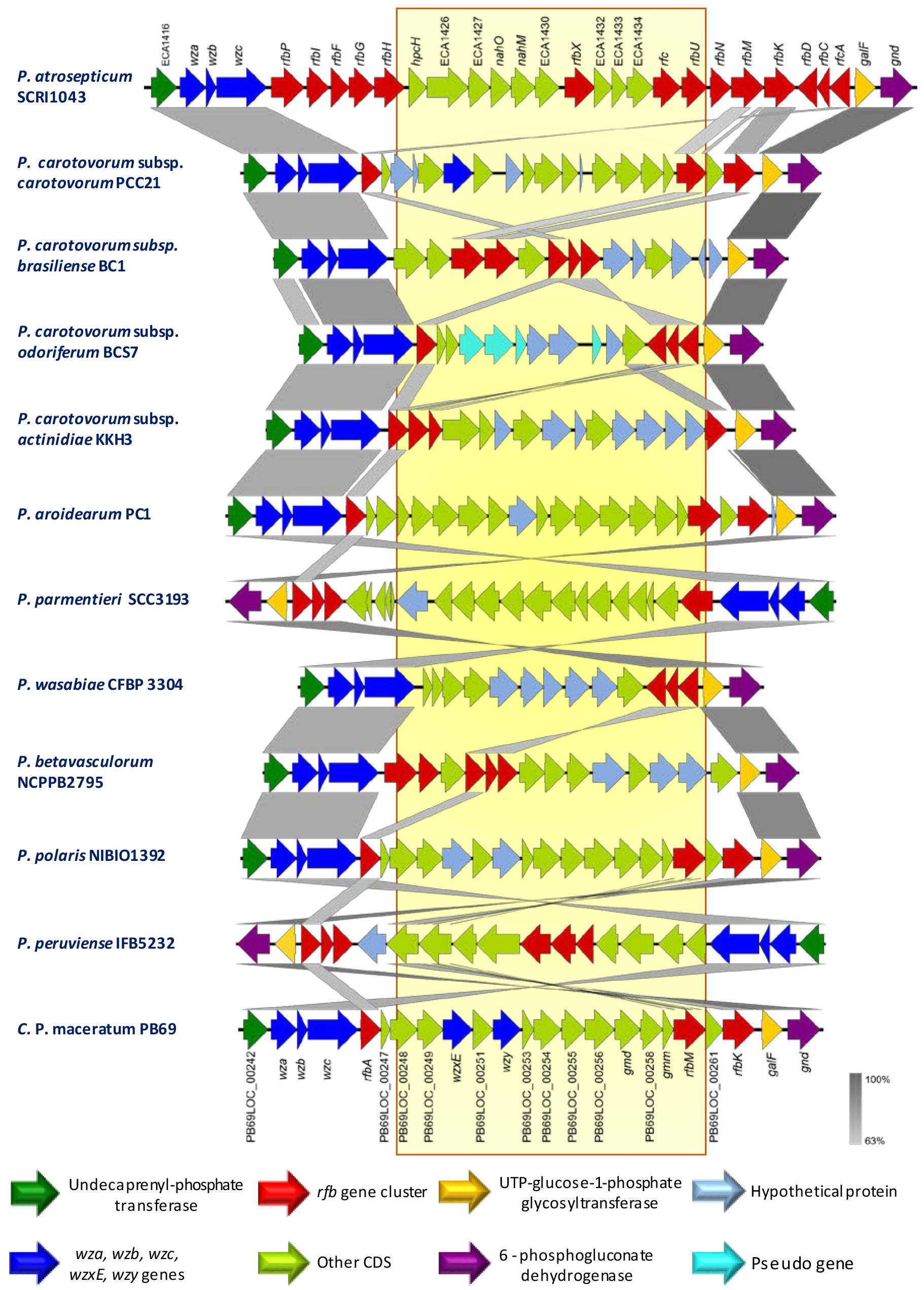

Supplement: Supplementary file 1 [file pathogens-08-00247-s001.zip › pathogens-633436supplementary/Supplementary Files/Figure S16.tif]

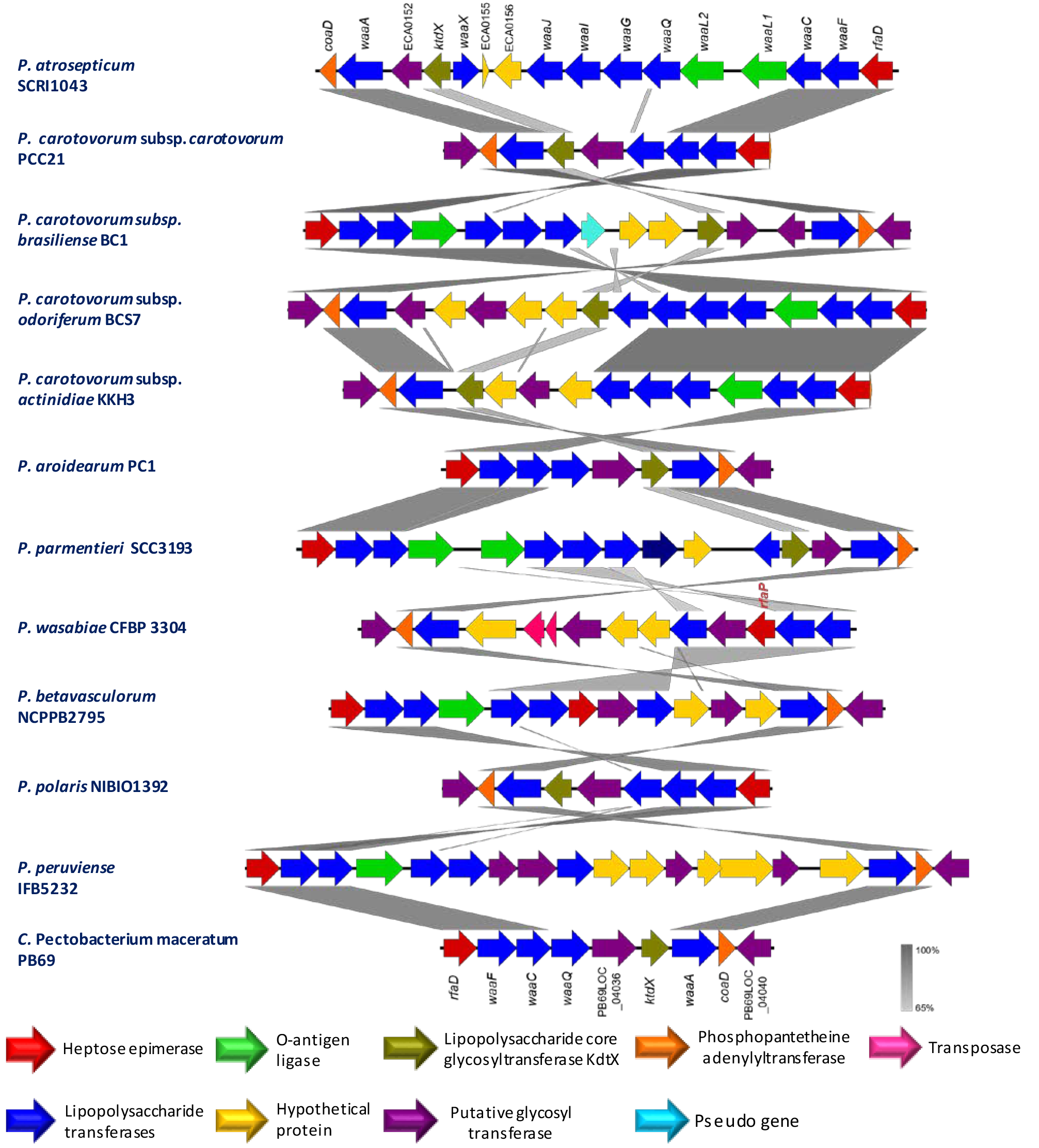

Supplement: Supplementary file 1 [file pathogens-08-00247-s001.zip › pathogens-633436supplementary/Supplementary Files/Figure S17.tif]

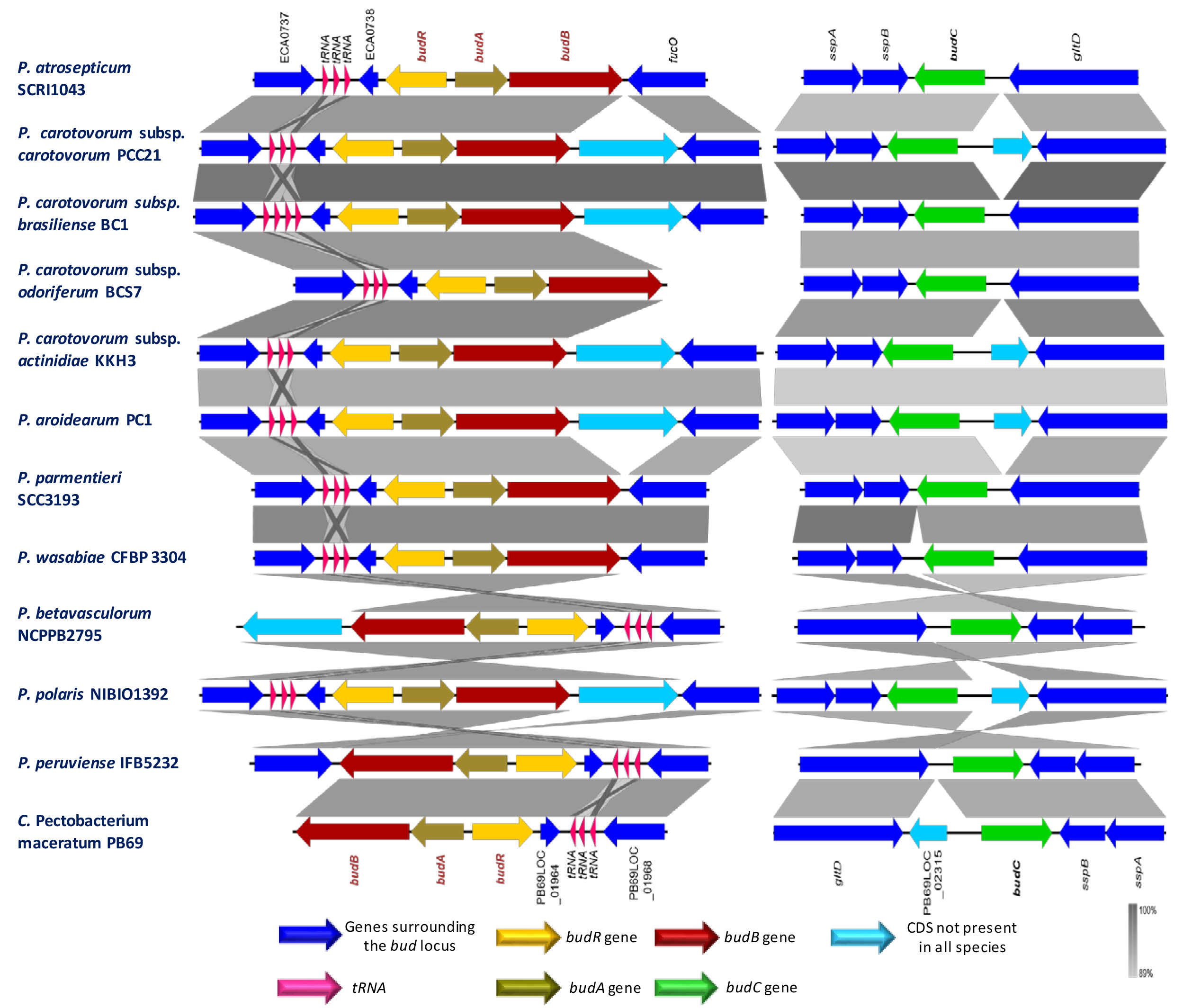

Supplement: Supplementary file 1 [file pathogens-08-00247-s001.zip › pathogens-633436supplementary/Supplementary Files/Figure S18.tif]

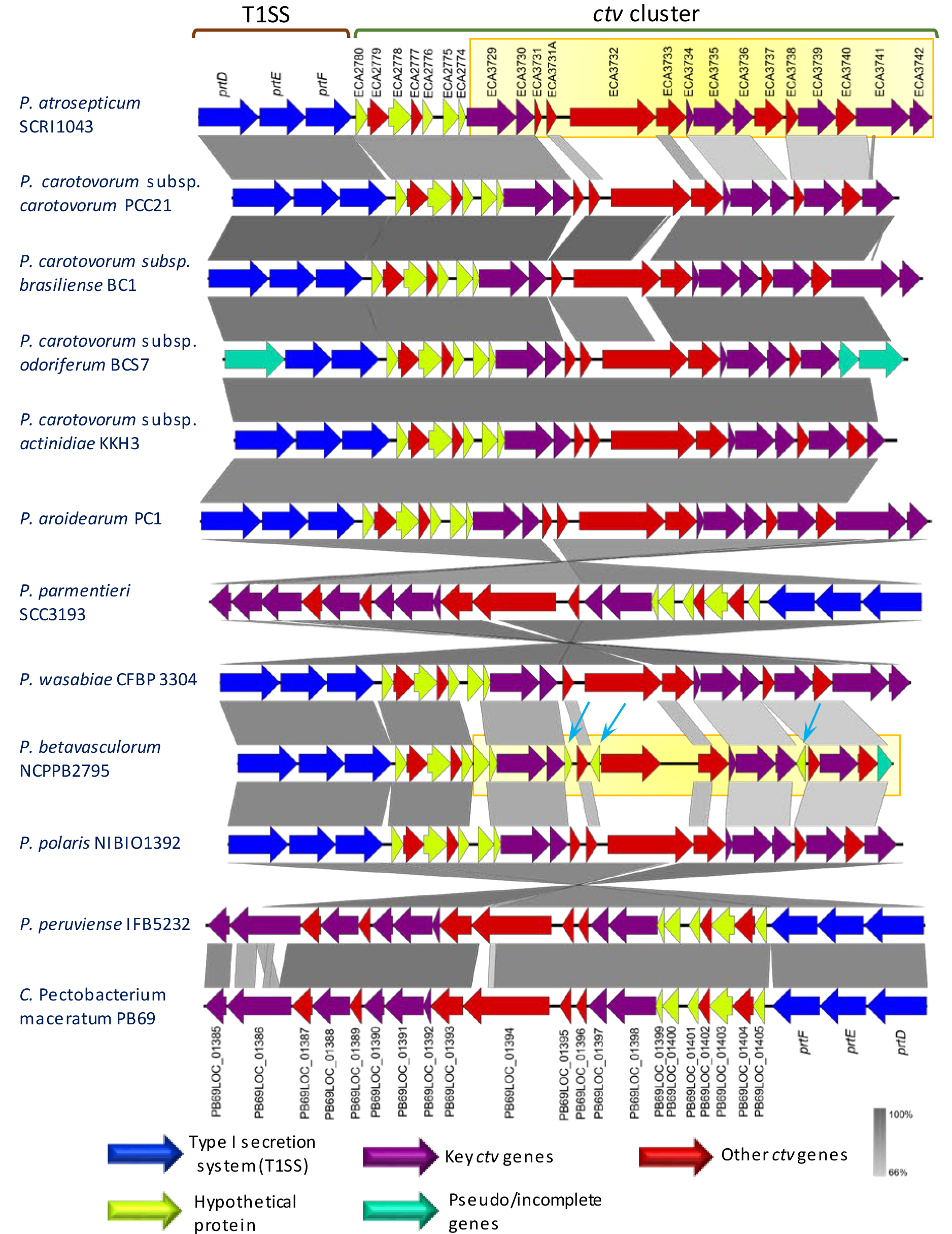

Supplement: Supplementary file 1 [file pathogens-08-00247-s001.zip › pathogens-633436supplementary/Supplementary Files/Figure S19.tif]

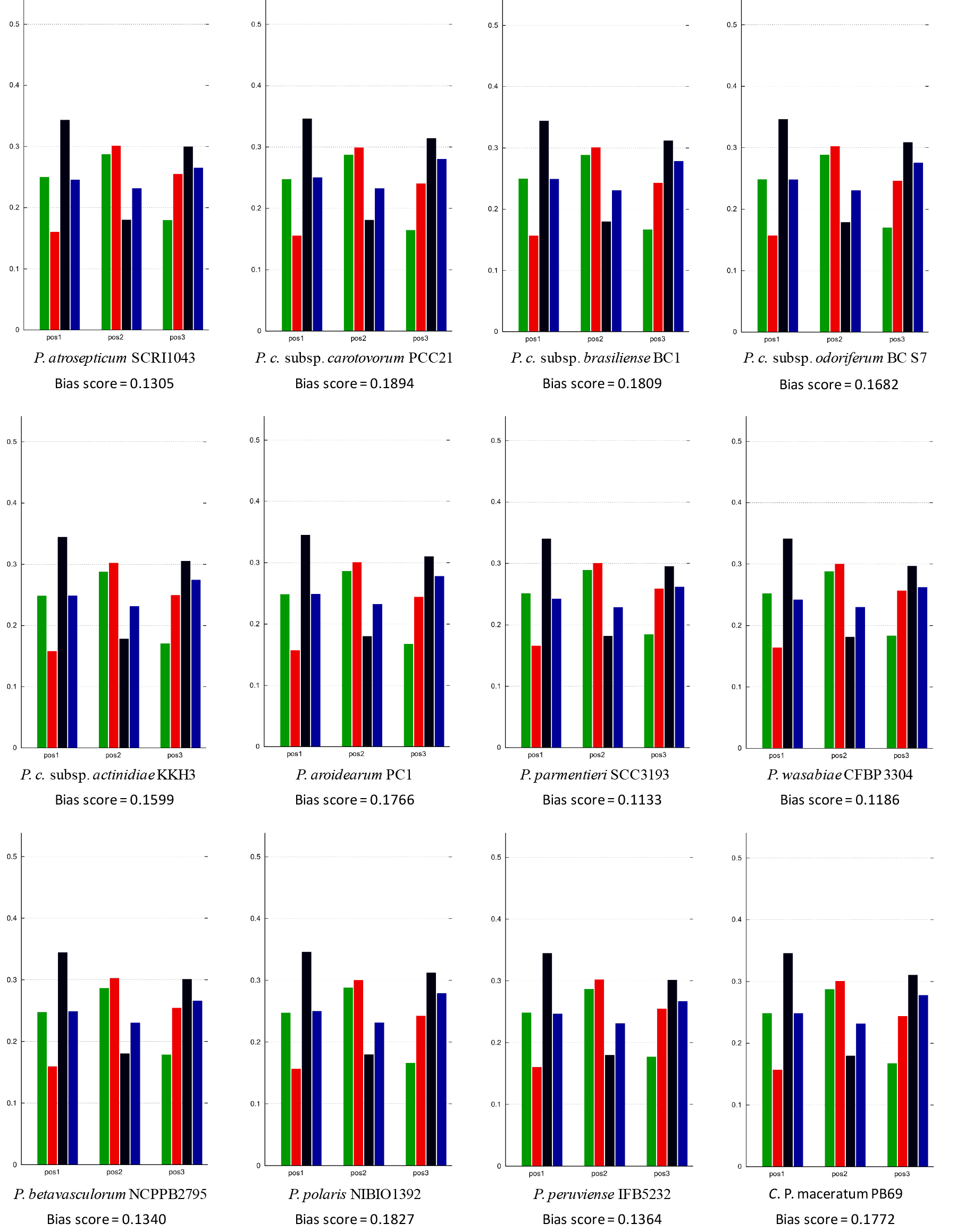

Supplement: Supplementary file 1 [file pathogens-08-00247-s001.zip › pathogens-633436supplementary/Supplementary Files/Figure S2.tif]

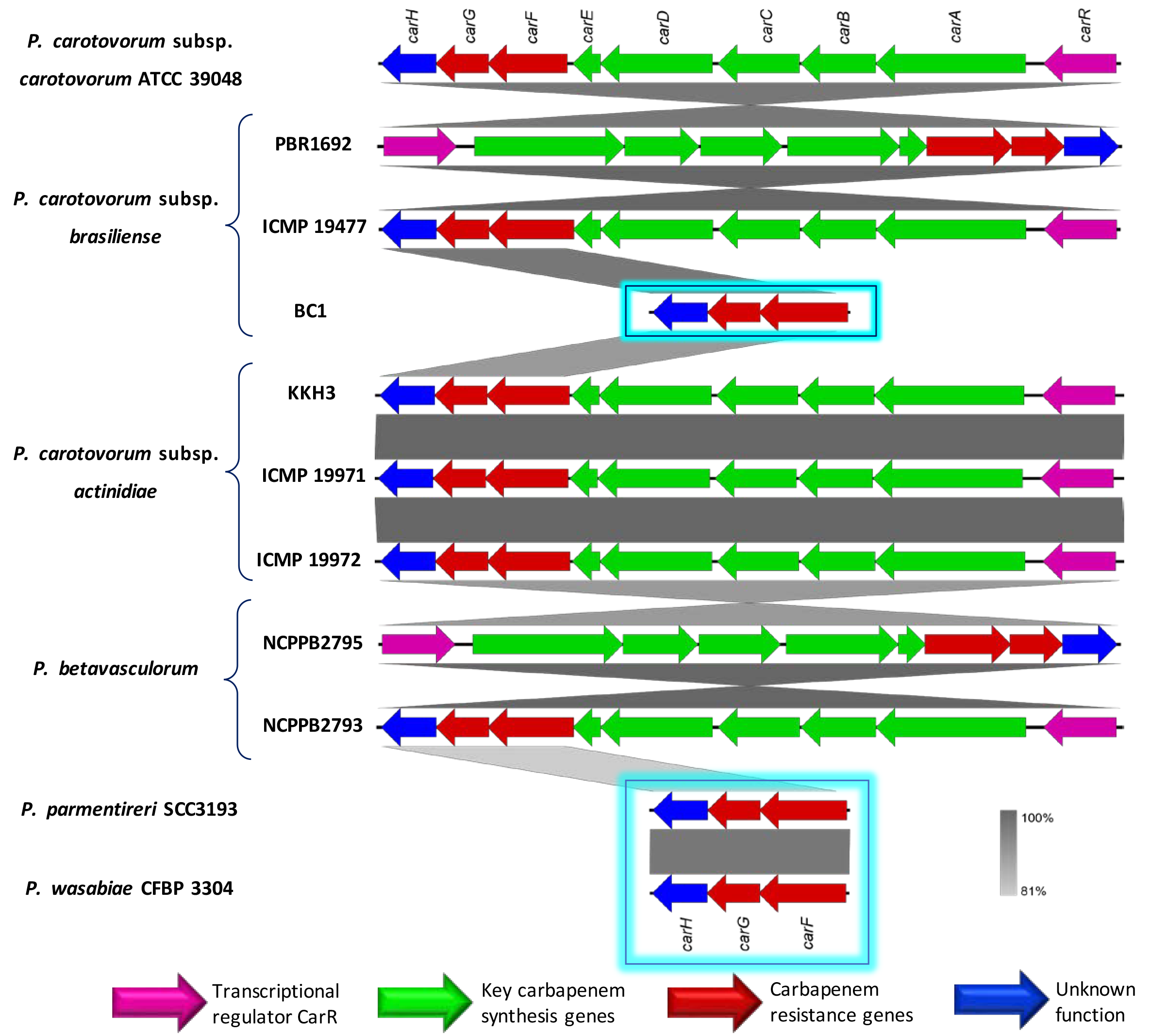

Supplement: Supplementary file 1 [file pathogens-08-00247-s001.zip › pathogens-633436supplementary/Supplementary Files/Figure S20.tif]

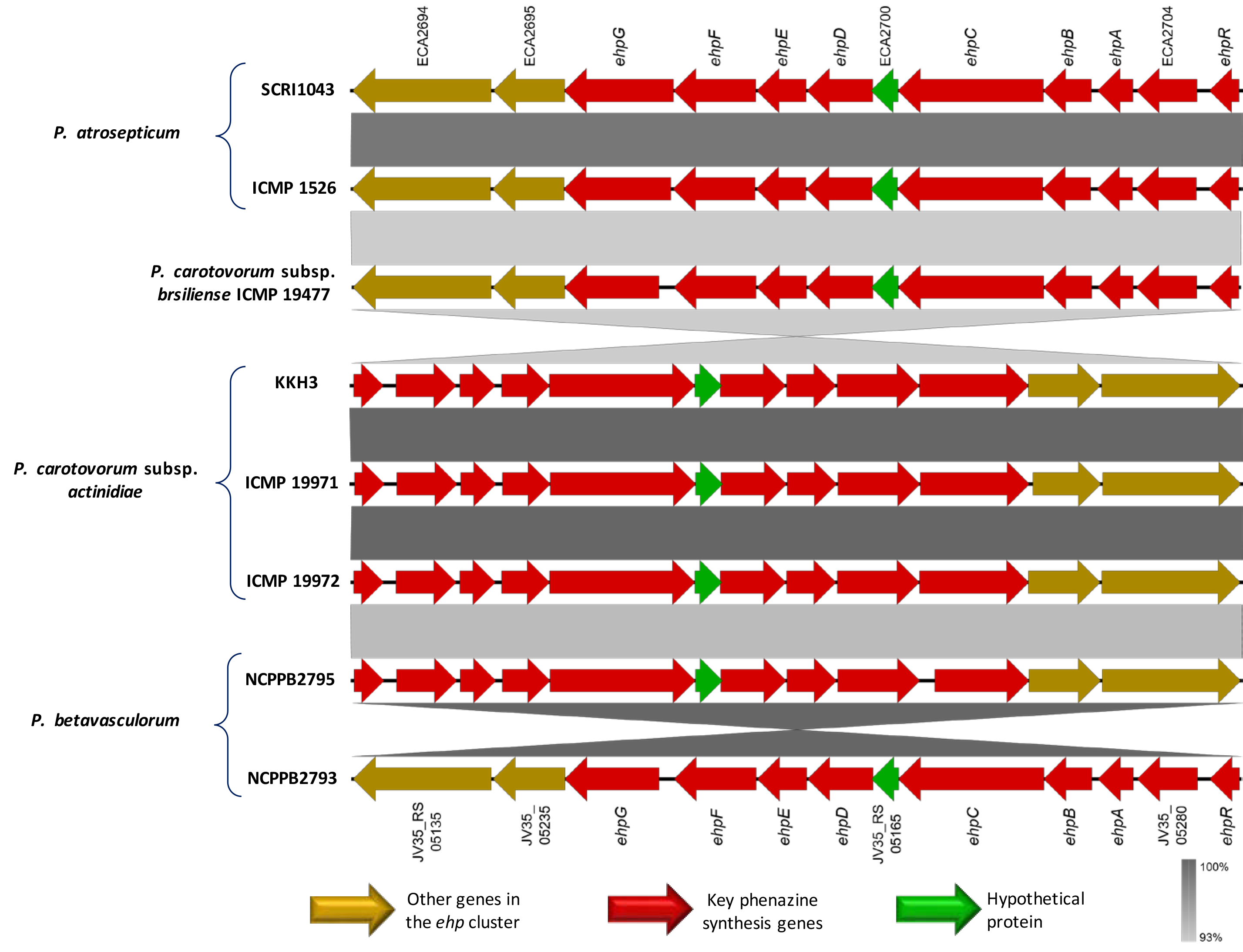

Supplement: Supplementary file 1 [file pathogens-08-00247-s001.zip › pathogens-633436supplementary/Supplementary Files/Figure S21.tif]

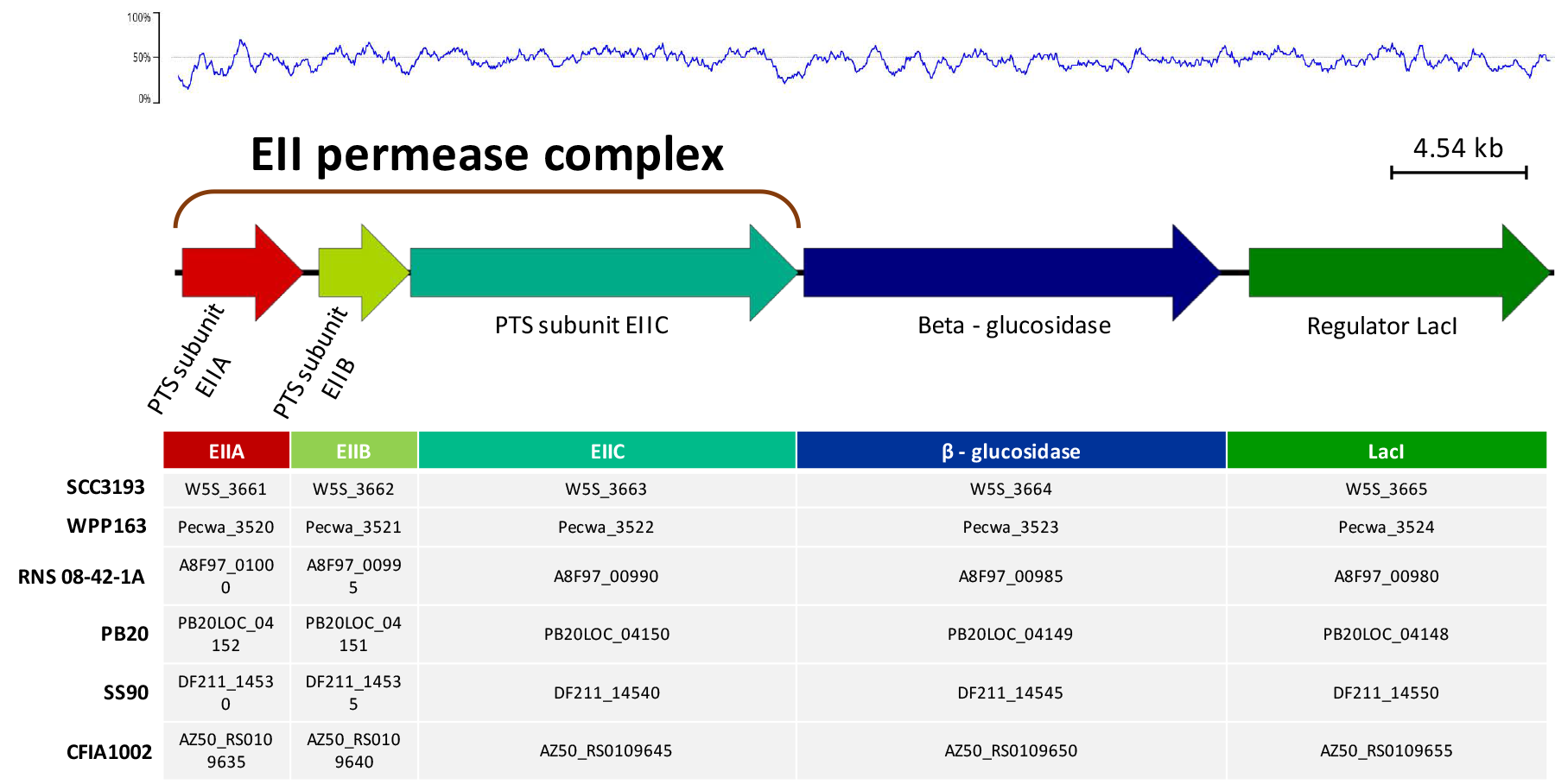

Supplement: Supplementary file 1 [file pathogens-08-00247-s001.zip › pathogens-633436supplementary/Supplementary Files/Figure S22.tif]

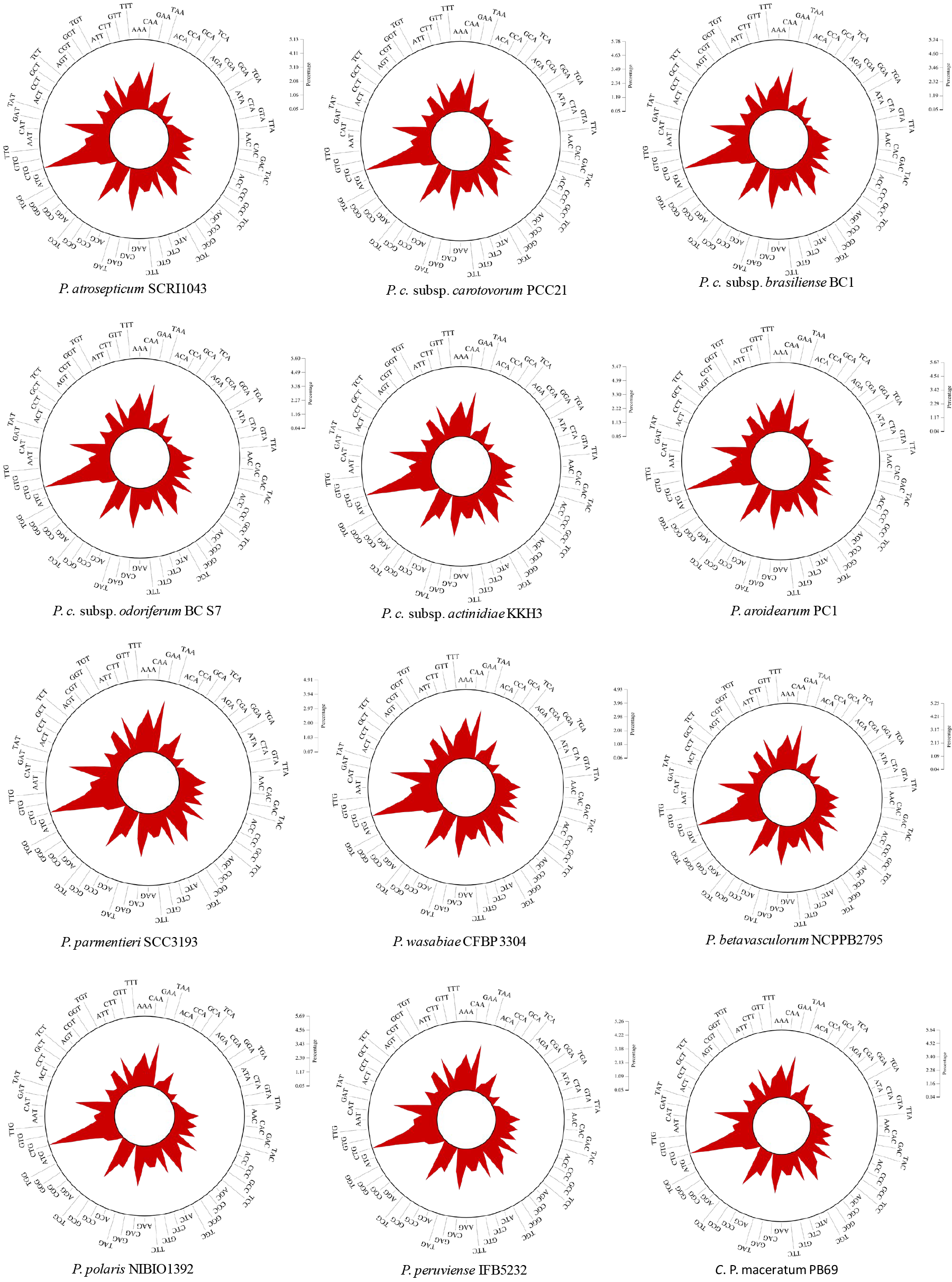

Supplement: Supplementary file 1 [file pathogens-08-00247-s001.zip › pathogens-633436supplementary/Supplementary Files/Figure S3.tif]

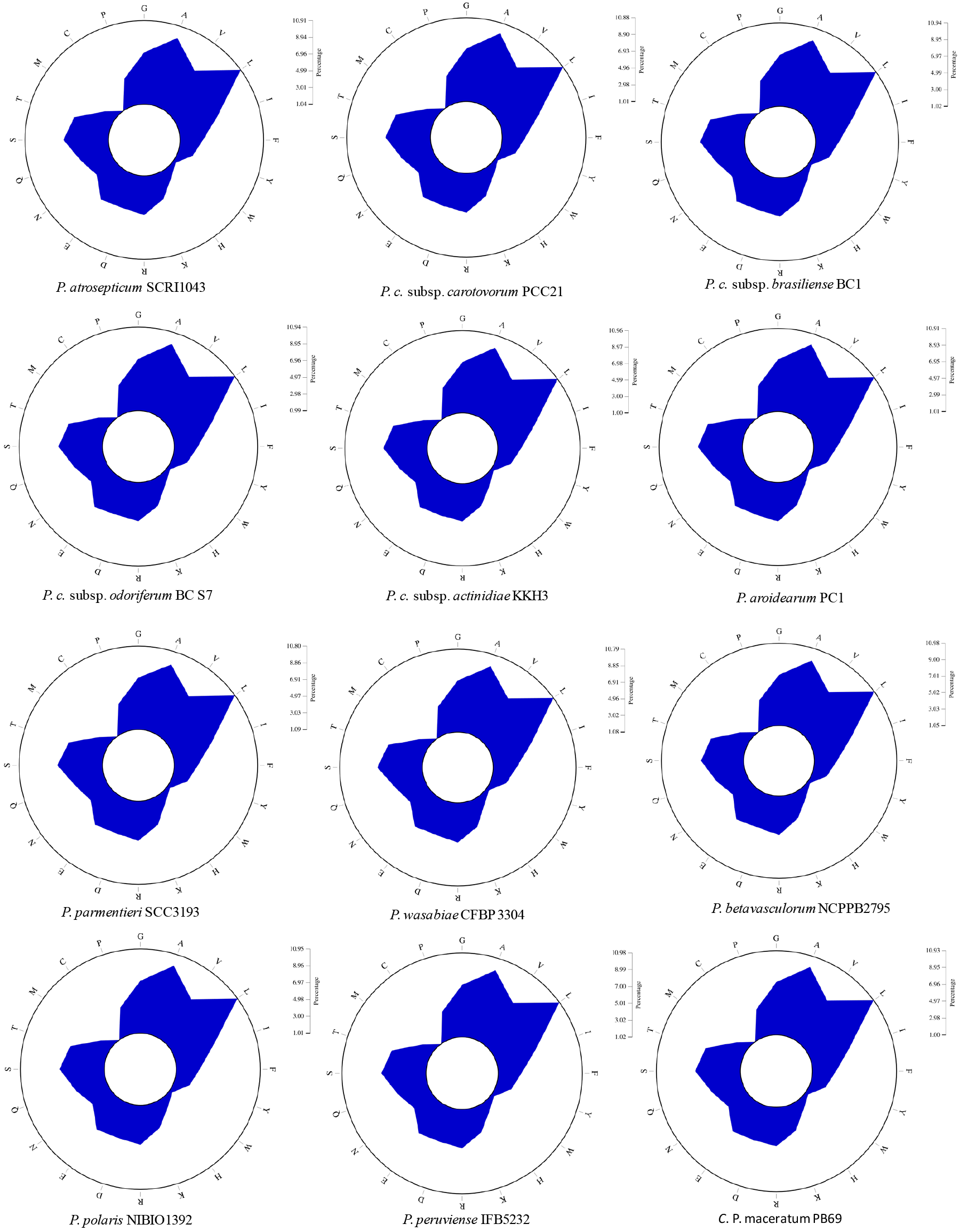

Supplement: Supplementary file 1 [file pathogens-08-00247-s001.zip › pathogens-633436supplementary/Supplementary Files/Figure S4.tif]

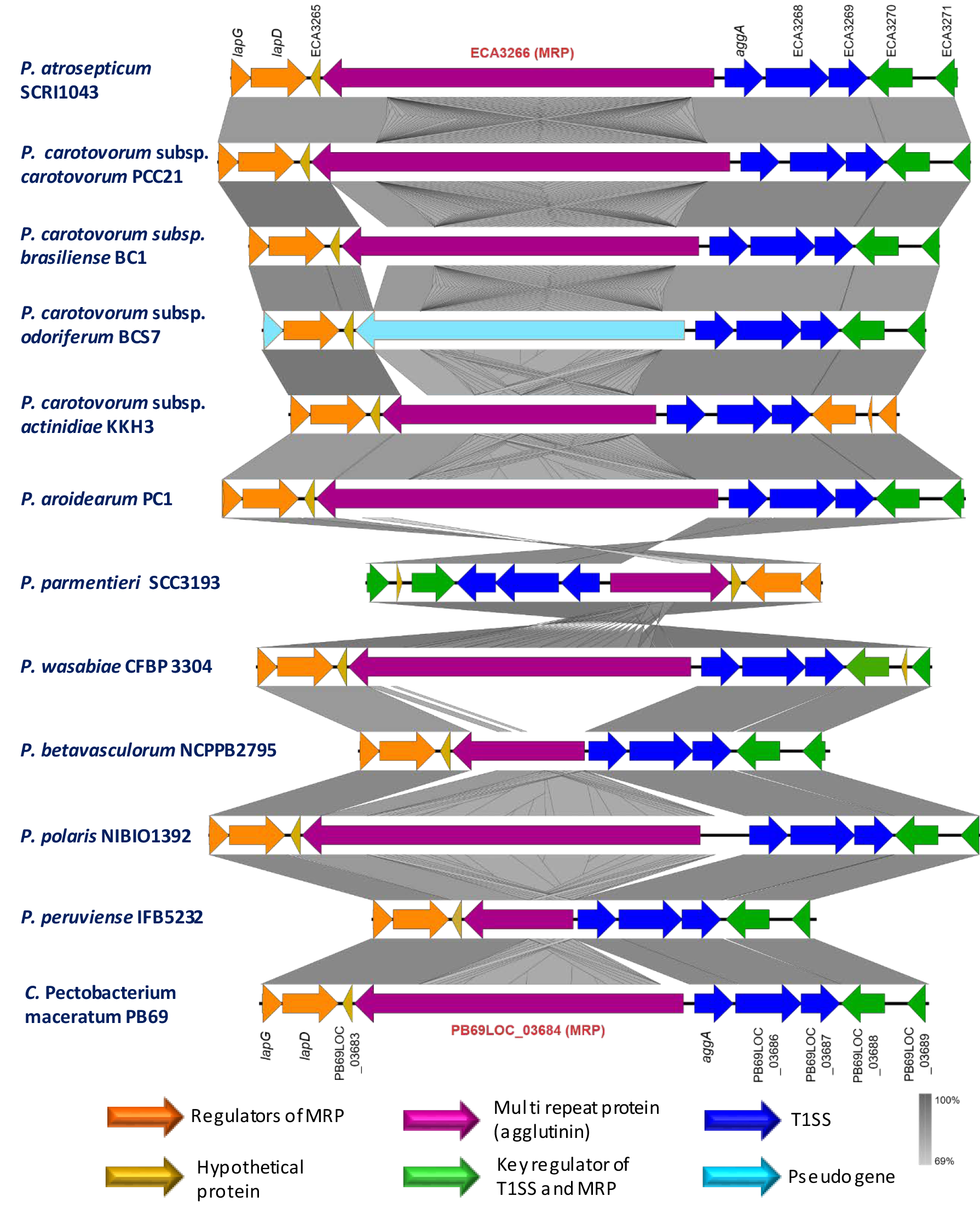

Supplement: Supplementary file 1 [file pathogens-08-00247-s001.zip › pathogens-633436supplementary/Supplementary Files/Figure S5.tif]

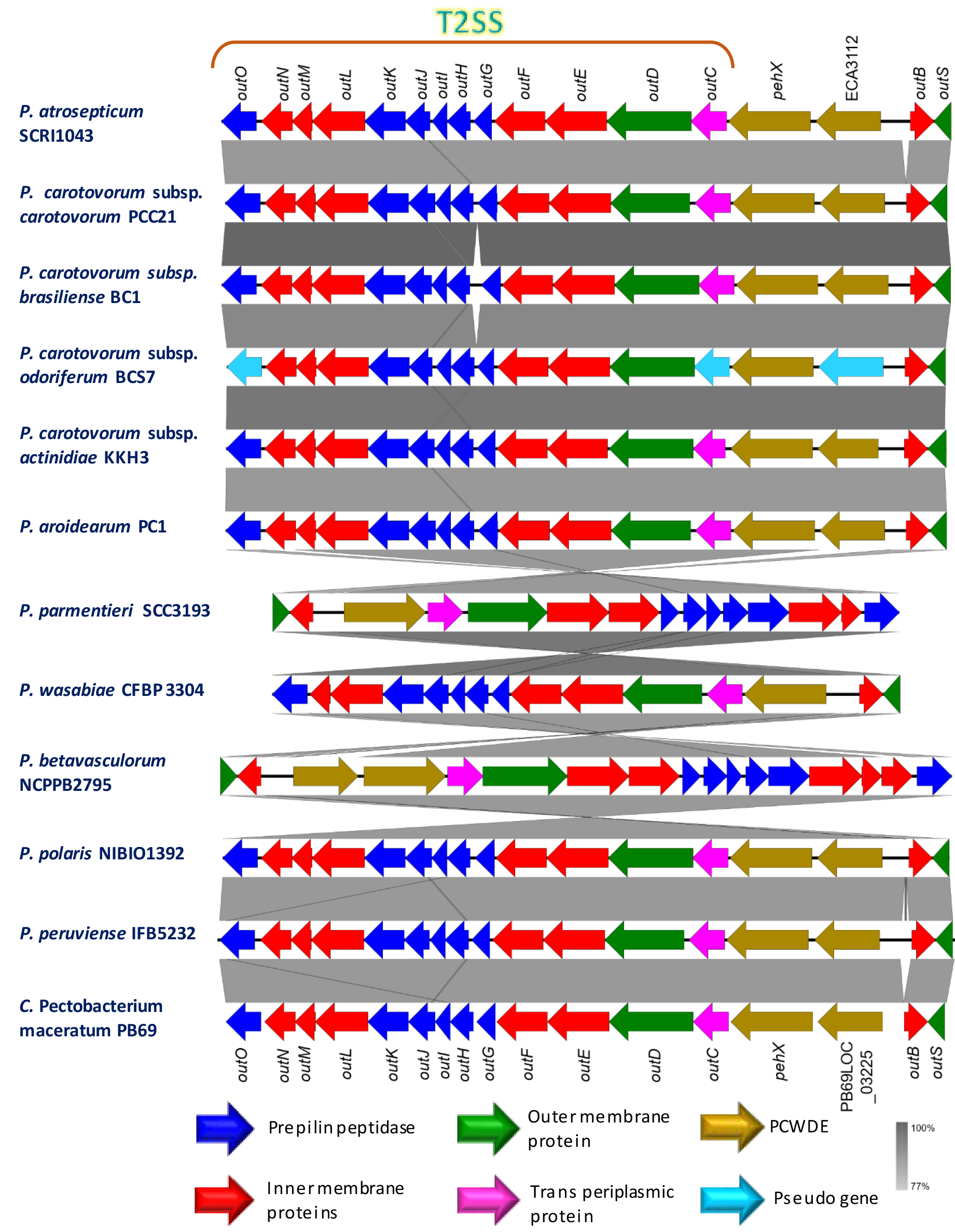

Supplement: Supplementary file 1 [file pathogens-08-00247-s001.zip › pathogens-633436supplementary/Supplementary Files/Figure S6.tif]

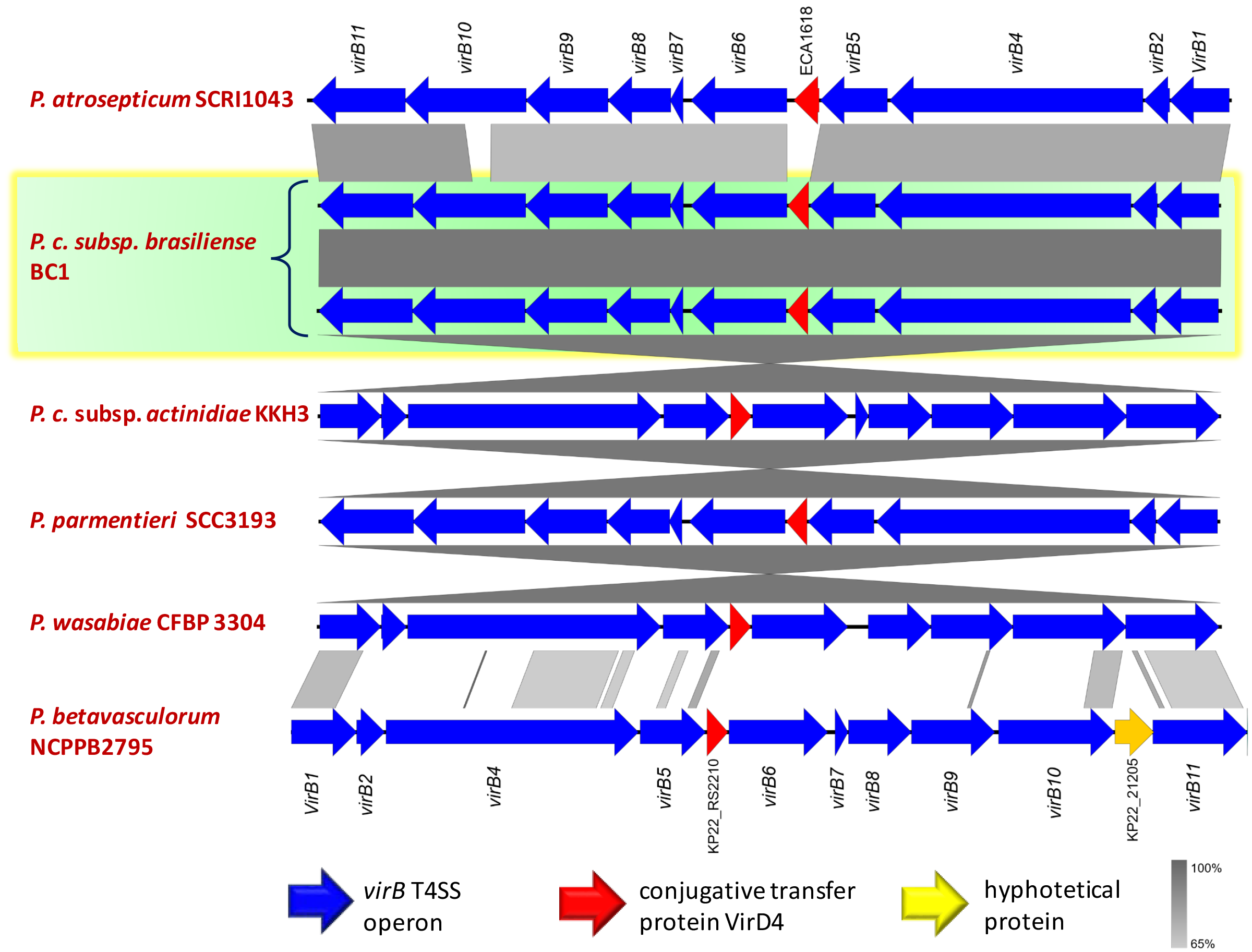

Supplement: Supplementary file 1 [file pathogens-08-00247-s001.zip › pathogens-633436supplementary/Supplementary Files/Figure S7.tif]

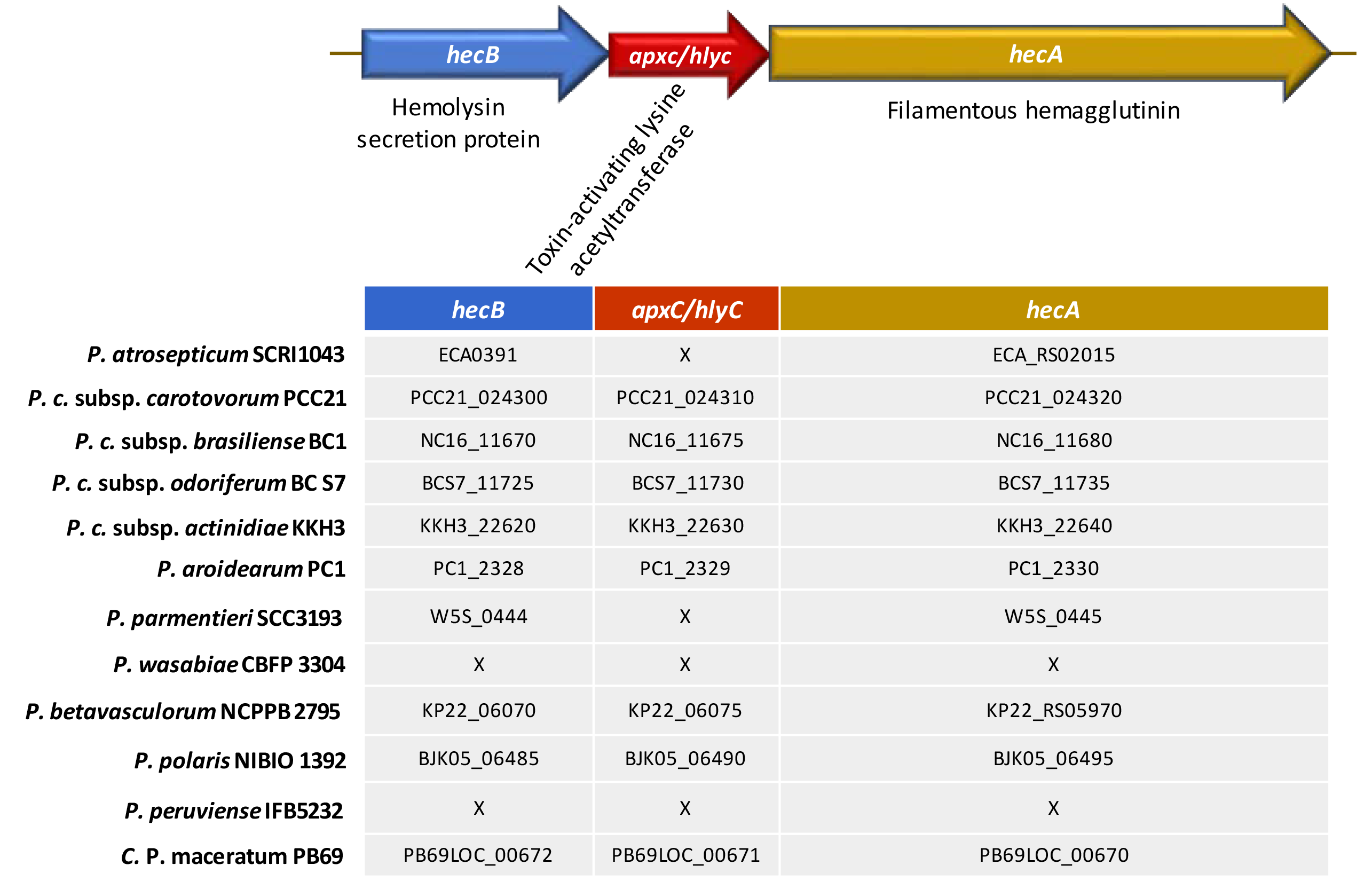

Supplement: Supplementary file 1 [file pathogens-08-00247-s001.zip › pathogens-633436supplementary/Supplementary Files/Figure S8.tif]

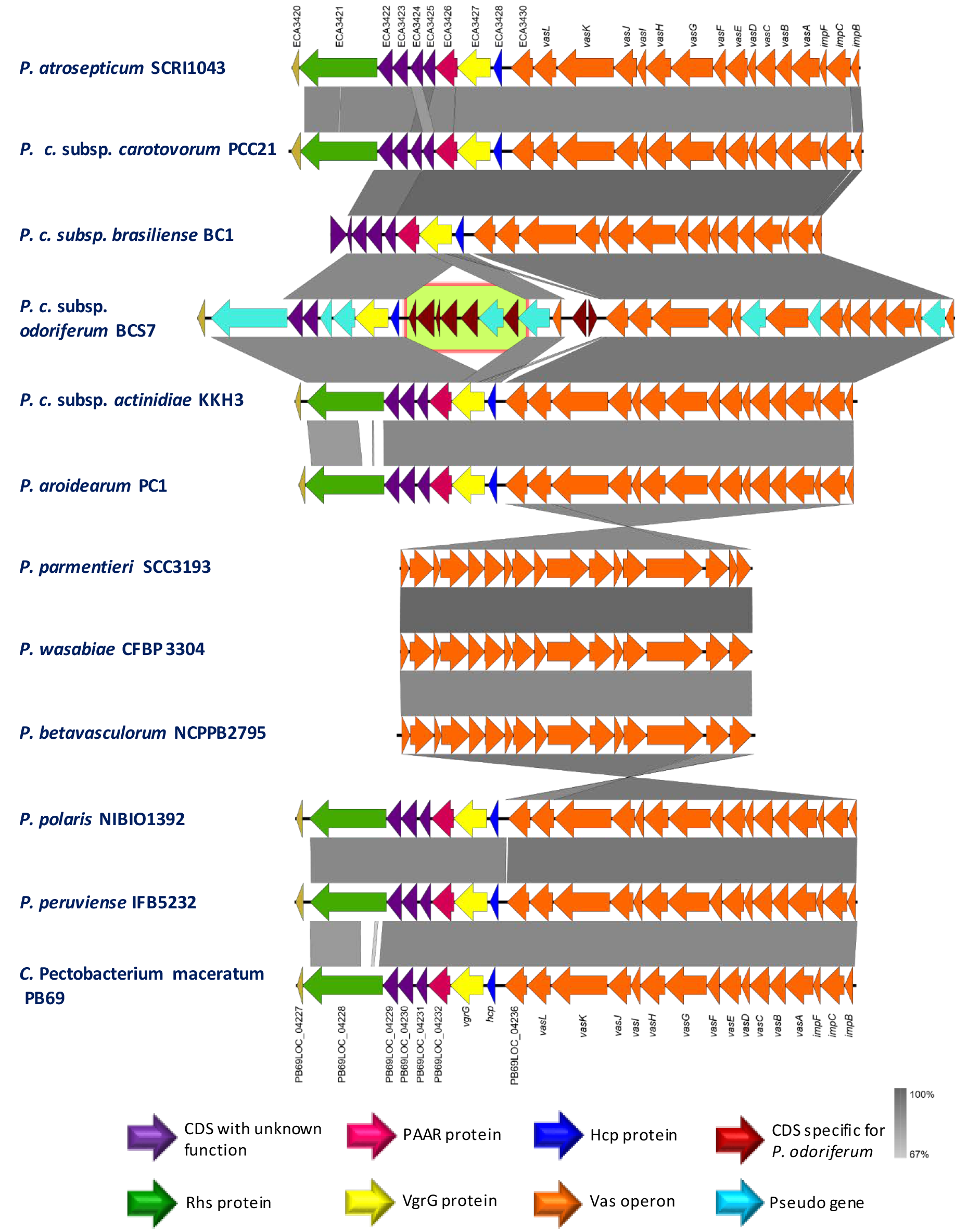

Supplement: Supplementary file 1 [file pathogens-08-00247-s001.zip › pathogens-633436supplementary/Supplementary Files/Figure S9.tif]
